# Uptake and toxicity of polystyrene micro/nanoplastics in gastric cells: Effects of particle size and surface functionalization

**DOI:** 10.1371/journal.pone.0260803

**Published:** 2021-12-31

**Authors:** Amrita Banerjee, Lloyd O. Billey, Weilin L. Shelver

**Affiliations:** USDA-Agricultural Research Service, Edward T. Schafer Agricultural Research Center, Biosciences Research Laboratory, Fargo, ND, United States of America; Xiangtan University, CHINA

## Abstract

Toxicity of micro or nanoplastics (MP/NP) in aquatic life is well-documented, however, information about the consequences of exposure to these particles in terrestrial species is scarce. This study was used to evaluate the uptake and/or toxicity of polystyrene MP/NP in human gastric cells, comparing doses, particle sizes (50, 100, 200, 500, 1000 or 5000 nm) and surface functionalization (aminated, carboxylated or non-functionalized). In general, the uptake of 50 nm particles was significantly higher than 1000 nm particles. Among the 50 nm particles, the aminated particles were more avidly taken up by the cells and were cytotoxic at a lower concentration (≥ 7.5 μg/mL) compared to same sized carboxylated or non-functionalized particles (≥ 50 μg/mL). High toxicity of 50 nm aminated particles corresponded well with significantly high rates of apoptosis-necrosis induced by these particles in 4 h (29.2% of total cells) compared to all other particles (≤ 16.8%). The trend of apoptosis-necrosis induction by aminated particles in 4 h was 50 > 5000 > 1000 > 500 > 200 > 100 nm. The 50 nm carboxylated or non-functionalized particles also induced higher levels of apoptosis-necrosis in the cells compared to 100, 1000 and 5000 nm particles with same surface functionalization but longer exposure (24 h) to 50 nm carboxylated or non-functionalized particles significantly (p<0.0001) increased apoptosis-necrosis in the cells. The study demonstrated that the toxicity of MP/NP to gastric cells was dependent on particle size, dose surface functionalization and exposure period.

## Introduction

Plastic is ubiquitous in our daily lives and its production has steadily increased from 1.5 million metric tons in 1950 to 368 million tons in 2019 [1]. Single use products form the bulk of the plastic produced in many parts of the world [2]. Consequently, plastic wastes that originate primarily from single use plastics have become an emerging environmental pollution issue globally. The novel coronavirus pandemic has aggravated plastic pollution due to sharp surge in the use of single-use plastics such as gloves and N95 or surgical masks [3]. If current trends in plastic production and waste management continue, landfills and water bodies will accumulate about 12,000 million metric tons of plastic waste by 2050, threatening marine and terrestrial ecosystems [4].

Discarded plastic can fragment into microplastics (MP; 100 nm—5 mm size) and/or nanoplastics (NP < 100 nm) due to natural weathering processes [5]. Aquatic or terrestrial biota exposed to MP/NP through ingestion or inhalation can accumulate the particles and suffer adverse consequences [6]. The detrimental effects of MP/NP exposure in aquatic species include embryotoxicity in zebrafish and sea urchins [7, 8], hepatotoxicity in zebrafish, goldfish and tadpoles [9–11], growth inhibition of microalgae and fish larvae [12–14], decreased life spans of shrimp and plankton upon long-term exposure [12, 15], deterioration of intestinal tissues in sea bass [16], and alterations in feeding behavior, metabolism and innate immunity in fish [17–19]. In terrestrial animals such as rodents, ingestion of MP/NP altered intestinal barrier function [20, 21], gut microbiome [21–23], metabolism [20, 24], behavior [25, 26], innate immunity [23], reproduction [27, 28] and cardiovascular function [29, 30].

Human exposure to MP/NP occurs through ingestion of contaminated food or water and inhalation of airborne plastic dust [31, 32]. Indeed, MP have been detected in seafood, drinking water, beer, condiments (salt, honey, sugar), as well as in the air around urban or remote areas [33–39]. Direct assessment of the effects of MP/NP exposure in humans have not been conducted but MP have been detected in human feces (20 particles per 10 g feces) [40]. Recently, MP were found in human placenta, both in the maternal and fetal portions as well as in the amniochorial membranes [41]. Moreover, plastic monomers were detected in several human organs such as lungs, liver, kidney, spleen and adipose tissue [42].

Currently, MP/NP are not regulated as food contaminants in the United States or the European Union. However, several reports on the trophic transfer of MP in the food chain, and toxicities in aquatic and terrestrial species, raise food safety concerns [6, 43]. Given that gastric cells are one of the cell types that MP/NP would encounter upon ingestion, we investigated the toxicity of polystyrene (PS) particles as model MP/NP in SNU-1 human gastric epithelial cells. Several sizes (50, 100, 200, 500, 1000 and 5000 nm) of MP/NP, encompassing nano- and microplastic designations, were studied. Additionally, aminated, carboxylated, and non-functionalized particles were studied to determine the effect of particle surface charge on cellular endpoints. Furthermore, toxicities of the particles were evaluated over large concentration (0.1–100 μg/mL) and time ranges (1–24 h) to cover different exposure scenarios. To the best of our knowledge, this is the first study to comprehensively evaluate the role of MP/NP properties such as particle size and surface functionalization in influencing uptake and cellular viability of human gastric epithelial cells.

## Materials and methods

### Materials

Aminated, carboxylated and non-functionalized (NF) polystyrene particles were purchased from Magsphere Inc. (Pasadena, CA, USA), Polysciences Inc. (Warrington, PA, USA), and Bangs Laboratories (Fishers, IN, USA), respectively. Yellow-green fluorescent (50, 100 and 1000 nm) aminated particles were bought from Magsphere Inc., while same sized yellow-green fluorescent carboxylated and NF particles were purchased from Polysciences Inc. The particles were either >99% pure or manufactured using highest purity materials. Disposable cuvettes (Cat # DTS0012 and ZEN0118) and folded capillary zeta cells (Cat # DTS1070) were obtained from Malvern Panalytical (Westborough, MA, USA). Human gastric carcinoma cell line, SNU-1 (CRL-5971), Roswell Park Memorial Institute (RPMI) 1640 culture media, and phosphate buffered saline (PBS) were purchased from ATCC (Manassas, VA, USA). Hank’s balanced salt solution (HBSS), fetal bovine serum (FBS), penicillin, streptomycin and amphotericin B solution (PSA) (100X, 10,000U/mL, 10 mg/mL, and 25 μg/mL respectively), Hoescht 33258 pentahydrate (bis-Benzimide), and Pierce^TM^ Coomassie (Bradford) protein assay kit were bought from Thermo Fisher Scientific (Waltham, MA, USA). T-150cm^2^ flasks, 96-well ultra-low attachment plates, and tissue culture plates (12 & 24 wells) were purchased from Corning Life Sciences (Tewksbury, MA, USA). Wheat germ agglutinin (WGA) Alexa Fluor 633 plasma membrane dye was obtained from Fisher Scientific, (Pittsburgh, PA, USA). Tween-20 and alamarBlue^®^ reagent were purchased from Bio-Rad (Hercules, CA, USA) while Triton X-100 and lipopolysaccharide (LPS) were obtained from Sigma Millipore (St. Louis, MO, USA). Fluorescein isothiocyanate (FITC) Annexin V and propidium iodide (PI) dyes were bought from BD Biosciences (San Jose, CA, USA). Caspase-3, 8, B-cell lymphoma 2 (Bcl-2) and Bcl-2 associated X (BAX) assay kits, and Caspase-3, 8 proteins were purchased from Abcam (Cambridge, MA, USA). Tumor necrosis factor-α (TNF-α) assay kit was obtained from Cayman Chemical (Ann arbor, MI, USA).

### Determination of size, homogeneity in size, and zeta potential of particles

Polystyrene beads were sterilized by heating the particles in silanized glass vials at 70°C for 24 h [44]. The mean hydrodynamic particle size, homogeneity in size distribution or polydispersity index (PDI) and zeta potential (a measure of net charge at particle-liquid interface) of all particles (100 μg/mL) were measured in water immediately after preparation (0 h) at room temperature (RT) or in RPMI containing 1% PSA with or without 0.0025% v/v Tween 20 at 0 h at RT and after 24 h incubation in a humidified CO_2_ incubator at 37 ˚C. The 50–200 nm particles were measured in a Zetasizer Ultra (Malvern Panalytical, Malvern, UK), the 500 and 1000 nm particles were measured in Zetasizer ZS90 (Malvern Panalytical, Malvern, UK), while the 5000 nm particles were measured in a Single Particle Optical Sizing instrument (SPOS, Particle Sizing Systems Inc., Santa Barbara, CA, USA). The zeta potential of 5000 nm particles was measured in the Zetasizer Ultra. Separate particle size measurement instruments were employed due to the inability of any single instrument to accurately measure sizes of all the particles utilized in this study. Particle size and PDI were measured simultaneously, followed by zeta potential measurements. Triplicate samples were prepared and measured for each particle type and condition.

### Scanning electron microscopy imaging of particles

The 50–5000 nm aminated, carboxylated and NF particles were imaged using a scanning electron microscope to determine their sizes in the absence of an aqueous dispersion media. Round glass coverslips 12 mm diameter (Assistent, Germany) were cleaned with ethanol and attached to cylindrical aluminum mounts (Ted Pella Inc., Redding, California, USA) with silver paint (SPI Supplies, West Chester, Pennsylvania, USA). Using a glass pipette, a drop of the sterilized particles was deposited on each mounted coverslip, placed in a covered container, and allowed to air-dry at RT. Mounted dried samples were coated with a conductive layer of carbon in a high-vacuum evaporative coater (Cressington 208c, Ted Pella Inc., Redding, California, USA). Images were obtained with a JEOL JSM-7600F scanning electron microscope (JEOL USA Inc., Peabody, Massachusetts) operating at 2 kV.

### Cell culture

SNU-1 cells were maintained at 37°C, with 5% CO_2_ under saturating humidity in RPMI supplemented with 10% FBS and 1% PSA. These suspension cells were propagated in T-150 flasks with the addition of fresh media every 2–3 days. Cells were counted using a Luna Brightfield automated cell counter (Logos Biosystems, Annandale, VA, USA). For all cell experiments described below, RPMI containing 1% PSA was used. Since gastric emptying occurs within 2–6 h of food ingestion, the cell based experiments were primarily conducted within this timeframe [45]. However, to determine long-term fate of the cells treated with the particles, toxicity (cell viability and apoptosis-necrosis) studies were extended to 24 h.

### Quantitation of cellular particle uptake

The ability of SNU-1 cells to internalize treatment particles was determined using flow cytometry. Yellow-green fluorescent dye labeled aminated, carboxylated, or NF PS particles (50, 100 or 1000 nm) were used. These particle sizes were selected based on high toxicity of 50 and 100 nm particles and to compare size dependent particle uptake of nanoplastics (50 nm particles), smaller microplastics (100 nm particles) and relatively larger microplastics (1000 nm particles). On the day of the study, each well of a 12-well plate was seeded with 1 mL of 1 x 10^6^ cells/mL cell suspension followed by the addition of 0.8 mL of RPMI containing 225 μg/mL particles and 0.0056% v/v Tween 20 to obtain a final particle concentration of 100 μg/mL particles and 0.0025% v/v Tween 20 in each well. In blank (untreated control) wells, 0.8 mL RPMI containing 0.0056% v/v Tween 20 was added to obtain a final concentration of 0.0025% v/v Tween 20 in the wells. Thereafter, the plates were placed in an incubator at 37 ˚C with 5% CO_2_ for 4 h under constant rotation at 100 rpm on a lateral shaker to simulate gastric motility. After 4 h, the cells were harvested through repeated pipetting (6–10 times) on an ice bath, the culture media containing any unabsorbed particles was discarded after centrifugation (200 *g*, 5 mins, 4 ˚C) and the cells were washed two times with 1 mL of ice-cold PBS by repeating the centrifugation and resuspension steps, to remove any surface adsorbed particles. The washed cells were resuspended in 0.3 mL ice-cold PBS and the particle uptake in the live-cell population was determined in a BD Accuri C6 flow cytometer (BD Biosciences, San Jose, CA, USA). For each run, 100,000 events were recorded. The scatter plot of forward scan and FITC channel obtained in the flow cytometer was gated to exclude cell debris. The FITC fluorescence from the gated population was quantified after blank subtraction using respective standard curves of each particle type. Percent cellular uptake of each type of particle was determined based on the amount of particles (180 μg) added to the wells at the start of the study.

### Qualitative assessment of cellular particle uptake

A qualitative assessment of particle uptake employed the same protocol as described in section 2.4. At the end of the 4 h treatment period, the culture media was removed, and the cells were washed using ice-cold PBS two times as described in the previous section. Cell pellets were then resuspended in 1 mL of neutral buffered formalin and incubated for 15 minutes at RT. Thereafter, formalin was removed, and the formalin fixed cells were washed in HBSS by centrifugation (200 *g*, 5 mins, 4 ˚C). To the washed cell pellet, 1 mL HBSS containing 5 μg/mL WGA Alexa 633 plasma membrane dye was added and the samples were incubated in the dark for 10 mins at RT. The cells were then washed with HBSS two times to remove the labeling solution and resuspended in 0.25 mL HBSS. To the plasma membrane labeled cells, Hoescht 33258 (5 μg/mL) was added and the cell samples were left in the dark for 30 mins. These samples were stored at 4 ˚C and the cells were imaged over subsequent days at 40 X magnification on a Zeiss Axio Observer Z1 laser scanning microscope 700 (Zeiss, Jena, Germany). Z-stack images were taken at every 0.3 μ increment for a total of about 20 μ distance. Images were analyzed using Imaris cell imaging software, version 9.5 (Oxford Instruments, Zurich, Switzerland).

### Effects of particles on cellular viability

Particle toxicity to SNU-1 cells was determined using the alamar Blue^®^ assay. On the day of the study, 100 μL of a cell suspension containing 5 x 10^4^ cells/mL were seeded in each well of ultra-low attachment clear 96 well plates. Immediately after cell plating, 80 μL of particle stocks (50–5000 nm aminated, carboxylated, or NF particles) in RPMI containing 0.225, 2.25, 22.5 or 225 μg/mL particles and 0.0056% v/v Tween 20 were added to each well to obtain final particle concentrations of 0.1, 1, 10, 100 μg/mL, respectively & 0.0025% v/v Tween 20. When toxicity was observed at a specific concentration, additional doses between the toxic and non-toxic concentrations were studied to determine the toxic concentration more precisely. For instance, additional doses of 2.5, 5 and 7.5 μg/mL were studied when toxicity was observed at 10 μg/mL while 25, 50, 75 μg/mL were used when toxicity was obtained at 100 μg/mL. Blank (untreated cells) comprising RPMI with 0.0025% v/v Tween 20 (final concentration in the wells) or positive control consisting of 0.25% v/v Triton X-100 (final concentration in the wells) were also used in the study. For each type of particle, experiments were conducted in triplicate (three plates) with 6 observation wells per replicate. After particle addition, the plates were incubated at 37°C for 1, 2, 4, 6 or 24 h, under constant lateral rotation at 100 rpm in humidified 5% CO_2_ atmosphere. At the end of treatment period, 20 μL of alamarBlue^®^ was added to each well and incubated for an additional 3 h. AlamarBlue^®^ reduction was quantified by measuring fluorescence at 545/590 nm (excitation/emission wavelengths) using a Wallac Victor X3 Multimode plate reader (Perkin Elmer, Waltham, MA, USA). Percentage cell viability was determined from fluorescence data normalized to blank values. A given treatment was considered toxic when a significant (p < 0.05) decrease in cell viability was observed compared to blank.

### Inflammatory response in SNU-1 cells

The ability of SNU-1 cells to secrete pro-inflammatory cytokines in response to inflammation stimulation was determined. Briefly, 500 μL of 5 x 10^5^ cells were seeded in 24 well plates and 400 μL of LPS was added to obtain final concentrations 0, 10, 100 or 1000 ng/mL in the wells. The plates were then incubated at 37 ˚C under constant rotation for 4, 6 or 12 h. At the end of the incubation periods, cells were collected after repeated pipetting and centrifuged at 200 *g* for 5 minutes at 4 ˚C. The supernatant was collected, bovine serum albumin (BSA) was added to the samples (2 mg/mL) and stored at -80 ˚C until further analysis. Cell pellets were lysed for 10 minutes on an ice bath using Milliplex MAP lysis buffer (Sigma Millipore, St. Louis, MO, USA) and the lysates were used to determine protein content. Concentration of TNF-α in the supernatant was determined following manufacturer instructions in the assay kit. Protein concentrations in the lysates was measured using the Bradford protein assay, employing BSA as the calibration standard and measuring absorbance at 595 nm in a Bio-Rad 550 microplate reader (Bio-Rad, Hercules, CA, USA).

### Effects of particles on early-stage cellular apoptosis

The potential of MP/NP in inducing apoptosis in SNU-1 cells was evaluated by determining Caspase-8, BAX and Bcl-2 production after 4 h of exposure. On the day of the study, each well of a 24 well plate was seeded with 0.5 mL of 2 x 10^6^ cells/mL cell suspension, followed by addition of 0.4 mL of RPMI containing 225 μg/mL aminated, carboxylated or NF particles of 50–5000 nm size and 0.0056% v/v Tween 20 to obtain a final concentration of 100 μg/mL particles and 0.0025% v/v Tween 20 in each well. To the blank wells (untreated cells), 0.4 mL of RPMI with 0.0056% v/v Tween 20 was added. The plates were then placed inside an incubator at 37 ˚C for 4 h in 5% CO_2_ and humidified atmosphere under constant lateral rotation at 100 rpm. At the end of treatment period, the plates were placed on an ice-bath, the cells were collected after repeated pipetting (6–10 times), and the culture media was removed by centrifugation (200 *g*, 10 mins, 4 ˚C). Cells were washed twice with 1 mL of ice-cold PBS by repeating the centrifugation and resuspension processes.

#### Caspase-8 assay

To the washed cell pellet, 50 μL of ice-cold lysis buffer (Abcam, Cambridge, MA, USA) was added and allowed to sit on ice for 10 mins. Thereafter, the lysed cells were centrifuged at 10,000 *g* for 10 mins at 4 ˚C. The supernatant was collected, and the protein content in the supernatant was measured using the Bradford protein assay as aforementioned. For caspase-8 quantitation, 50 μg protein from each sample was added to a 96 well plate and manufacturer instructions were followed. Samples were analyzed at 405 nm in Tecan Ultra 384 multi-detection microplate reader (Tecan, Männedorf, Switzerland). As an assay control, Caspase-8 protein (1 unit) was added to 50 μg lysate of blank treatment and analyzed.

#### BAX/Bcl-1 assays

Cells washed with PBS were lysed for 20 minutes in an ice-bath using 60 μL of ice-cold 1X cell extraction buffer PTR (provided in the BAX/Bcl-2 assay kits). The cell lysates were then centrifuged for 20 minutes at 18,000 *g* at 4 ˚C and the supernatants were collected for BAX/Bcl-2 and protein assays. BAX/Bcl-2 assays were performed as per manufacturer protocols and the concentration of BAX/Bcl-2 in the samples were measured at 450 nm in a Tecan Ultra 384 multi-detection microplate reader. Protein content was measured using Bradford protein assay as aforementioned.

### Late-stage cellular apoptosis and necrosis induced by particles

Late-stage apoptosis and necrosis in the cells were analyzed using Caspase-3 assay and FITC Annexin V-PI assay following the manufacturer’s protocol. Briefly, each well of a 24-well plate was seeded with 0.5 mL of 2 x 10^6^ cells/mL and 0.4 mL of RPMI containing 225 μg/mL aminated, carboxylated or NF particles (50–5000 nm) and 0.0056% v/v Tween 20 to obtain a final concentration of 100 μg/mL particles and 0.0025% v/v Tween 20 in the wells. To the blank wells (untreated cells), 0.4 mL of RPMI with 0.0056% v/v Tween 20 was added. The plates were then incubated at 37 ˚C under 5% CO_2_ atmosphere with constant lateral rotation at 100 rpm for 4 h (for Caspase-3 assay) or 4 and 24 h (for Annexin V-PI assay). At the end of treatment period, the cells were harvested by repeated pipetting (6–10 times), the culture media was removed by centrifugation (200 *g*, 5 mins, 4 ˚C), and the cell pellet was washed two times using 1 mL ice-cold PBS with repeated resuspension and centrifugation.

#### Caspase-3 assay

The washed cells were lysed in 50 μL ice-cold lysis buffer for 10 minutes. The samples were thereafter centrifuged at 10,000 *g* at 4 ˚C and the supernatants were collected. Protein concentration in the supernatant was measured using Bradford protein assay and Caspase-3 was quantitated using 50 μg protein from each sample following manufacturer protocol. Samples were analyzed at 405 nm in Tecan Ultra 384 multi-detection microplate reader. For assay control, Caspase-3 protein (1 unit) was added to 50 μg blank lysate and analyzed.

#### Annexin V-PI assay

To the washed cells, 1 mL of the binding buffer (provided in the Annexin V-PI kit) was added. Thereafter, to 100 μL of this cell suspension, 5 μL of FITC Annexin V and PI was added, the samples were gently vortexed, and then left in the dark for 15 mins at RT. The live cell population was analyzed in a flow cytometer using a plot of PI *vs* FITC fluorescence within 1 h of dye addition. For each run in the flow cytometer, 20,000 events were recorded. The cell population was gated to exclude debris and the percentages of apoptotic-necrotic cells were determined by identifying the population demonstrating both high FITC and PI fluorescence.

### Data analysis

Data are represented as means ± standard deviations (S.D.). The statistical analyses were conducted using two-way analysis of variance followed by Tukey’s honestly significant difference (HSD) post-hoc test (JMP^®^ 15.1, copyright 2020, SAS Institute Inc., Cary, NC, USA or GraphPad Prism 9.1.2, San Diego, CA, USA). A p value < 0.05 was considered statistically significant. All graphs were plotted using Graphpad Prism software, version 9.1.2.

## Results and discussion

### Characterization of polystyrene particles

The hydrodynamic particle size, PDI, and zeta potential of PS particles across various sizes and surface functionalization were measured in water and RPMI cell culture media. Particle size measurement in water helped us compare our results with respect to size in the manufacturer’s certificate of analysis (CoA), while measurements in RPMI provided insights about particle characteristics in culture media. Size stability of particles under experimental conditions, i.e., after incubation in RPMI for 24 h at 37 ˚C in a CO_2_ atmosphere, was also assessed.

As shown in Table 1, particle size in water correlated fairly well with the manufacturer’s CoA. However, when particles were placed in RPMI, some types of particles aggregated and this persisted or even increased upon incubation at 37 ˚C for 24 h. Specifically, 100 and 200 nm aminated particles, 50 and 500 nm carboxylated particles and 50 nm NF particles measured larger sizes in RPMI compared to water. Upon incubation at 37 ˚C for 24 h, the size measured for 100 nm particles almost tripled, while the size of other aggregated particles remained similar to size measured at 0 h. Interestingly, 500 nm aminated particles did not aggregate in RPMI at the onset (0 h) but the size increased 6-fold upon incubation at 37 ˚C for 24 h. To mitigate the aggregation propensity of the particles, Tween 20 (a nonionic surfactant) was used at a concentration of 0.0025% v/v. This concentration did not adversely affect viability of SNU-1 cells for at least 6 h (S1 Fig). Incorporation of Tween 20 prevented aggregation of 100 and 200 nm aminated particles, 50 and 500 nm carboxylated particles and 50 nm NF particles in RPMI at 0 h as well as upon incubation at 37 ˚C for 24 h. The size of all the other particles in RPMI were also stable in presence of Tween 20 after 24 h incubation at 37 ˚C and were comparable to their respective sizes in water. Scanning electron microscopy images of the particles confirmed the results obtained from size measurements using dynamic light scattering (S2A and S2B Fig).

**Table 1:** Effect of matrix and storage conditions on particle size of polystyrene micro/nanoplastics (mean ± S.D.; n = 3).

Size (nm)						
Sterilized particle solutions (100 μg/mL)	CoA^1^	Water (0 h at RT^2^)	RPMI (0 h at RT)	RPMI + 0.0025% v/v Tween 20 (0 h at RT)	RPMI (stored at 37 ˚C for 24 h)	RPMI + 0.0025% v/v Tween 20 (stored at 37 ˚C for 24 h)
**50 nm Amine**	53	56.8 ± 1.4	59.7 ± 0.5	63.5 ± 1.0	59.6 ± 0.7	56.3 ± 0.4
**100 nm Amine**	97	103. 2 ± 0.7	1087.6 ± 293.7	110.5 ± 1.1	3854.3 ± 1364.5	99.0 ± 0.7
**200 nm Amine**	200	244.8 ± 49.0	3082.3 ±1255.8	222.1 ± 2.7	3335.0 ± 335.9	213.3 ± 0.5
**500 nm Amine**	540	656.8 ± 16.9	746.6 ± 32.5	709.4 ± 15.4	5,050.5 ± 497.3	687.3 ± 19.7
**1000 nm Amine**	950	970.2 ± 4.3	1070.7 ± 0.5	1081.2 ± 20.8	972.7 ± 24.2	987.5 ± 57.7
**5000 nm Amine**	5300	5750.0 ± 167.0	5500.0 ± 190.5	5663.3 ± 285.9	6033.3 ± 117.2	5940.0 ± 10.0
**50 nm Carboxyl**	49	58.3 ± 0.5	1375.3 ± 102.6	62.3 ± 0.2	1661.0 ± 180.7	59.7 ± 0.5
**100 nm Carboxyl**	104	105.2 ± 0.3	119.5 ± 2.0	111.4 ± 0.2	131.3 ± 8.5	108.4 ± 0.6
**200 nm Carboxyl**	200	212.2 ± 1.1	222.5 ± 1.3	218.7 ± 6.8	220.7 ± 4.0	219.6 ± 1.3
**500 nm Carboxyl**	520	524.6 ± 1.9	1,160.7 ± 56.5	559.1 ± 8.5	969.8 ± 71.3	555.1 ± 13.1
**1000 nm Carboxyl**	988	962.1 ± 10.8	1444.9 ± 36.0	1033.2 ± 36.6	1401.8 ± 65.6	951.0 ± 26.9
**5000 nm Carboxyl**	4700	3593.3 ± 45.1	3680.0 ± 98.5	3610.0 ± 20.0	3993.3 ± 303.5	3746.7 ± 51.3
**50 nm NF** ^**3**^	44	45.1 ± 0.6	2945.3 ± 2498.9	51.4 ± 0.9	3364.3 ± 1329.4	58.5 ± 1.1
**100 nm NF**	88	95.5 ± 0.7	101.6 ± 0.5	106.3 ± 2.0	125.6 ± 0.6	105.6 ± 1.2
**200 nm NF**	195	202.0 ± 3.3	220.3 ± 15.4	211.9 ± 6.2	210.8 ± 0.5	215.6 ± 1.2
**500 nm NF**	503	521.2 ± 11.6	552.8 ± 6.4	542.5 ± 7.2	549.9 ± 11.5	541.0 ± 3.4
**1000 nm NF**	1040	1017.9 ± 56.9	1351.0 ± 42.1	1122.7 ± 39.3	1237.9 ± 108.2	958.2 ± 25.0
**5000 nm NF**	4610	3470.0 ± 20.0	3433.3 ± 58.6	3450.0 ± 26.5	3636.7 ± 151.8	3490.0 ± 104.4

Another important particle characteristic is PDI, which describes the uniformity of the particle size distribution. A PDI value of 0.0 represents a perfectly homogenous particle size distribution (monodispersed population) while values ≥ 1.0 represent highly heterogenous particle size distributions (polydispersed population) [46]. Values ≤ 0.2 are considered acceptable for polymer-based particle formulations [46]. The 50–1000 nm particles in water demonstrated PDI mostly ≤ 0.2, except for 1000 nm NF particles which had a PDI of 0.3 (Table 2). The PDI of 5000 nm particles could not be measured with SPOS. When the particles were dispersed in RPMI, the PDI at 0 h increased to > 0.2 for 100, 200, and 1000 nm aminated, 50 and 1000 nm carboxylated and NF particles. After 24 h incubation at 37 ˚C, the PDI remained > 0.2 for the 100, 200 nm aminated, 50, 1000 nm carboxylated and 50 nm NF particles. Moreover, the PDI of 500 nm aminated particles more than tripled at 24 h compared to 0 h, which corresponded to aggregation of these particles observed at 24 h. Addition of 0.0025% v/v Tween 20 helped maintain PDI ≤ 0.2 for most particles at both 0 h and 24 h measurement time points. The exceptions were the 1000 nm carboxylated and NF particles, which exhibited a PDI 0.3–0.4 at 24 h.

**Table 2:** Polydispersity indices of polystyrene micro/nanoplastics (mean ± S.D.; n = 3).

Polydispersity index					
Particle solutions (100 μg/mL)	Water (0 h at RT)	RPMI (0 h at RT)	RPMI + 0.0025% v/v Tween 20 (0 h at RT)	RPMI (stored at 37 ˚C for 24 h)	RPMI + 0.0025% v/v Tween 20 (stored at 37 ˚C for 24 h)
**50 nm Amine**	0.03 ± 0.00	0.02 ± 0.03	0.03 ± 0.01	0.02 ± 0.02	0.04 ± 0.02
**100 nm Amine**	0.04 ± 0.01	0.56 ± 0.04	0.02 ± 0.00	0.29 ± 0.24	0.02 ± 0.01
**200 nm Amine**	0.08 ± 0.10	0.27 ± 0.04	0.02 ± 0.02	1.09 ± 0.53	0.06 ± 0.02
**500 nm Amine**	0.21 ± 0.03	0.11 ± 0.02	0.11 ± 0.06	0.39 ± 0.12	0.18 ± 0.05
**1000 nm Amine**	0.15 ± 0.03	0.34 ± 0.15	0.24 ± 0.09	0.13 ± 0.03	0.19 ± 0.08
**5000 nm Amine**	n/a^1^	n/a	n/a	n/a	n/a
**50 nm Carboxyl**	0.04 ± 0.00	0.25 ± 0.03	0.02 ± 0.03	0.36 ± 0.04	0.04 ± 0.01
**100 nm Carboxyl**	0.01 ± 0.00	0.03 ± 0.00	0.02 ± 0.01	0.05 ± 0.01	0.01 ± 0.01
**200 nm Carboxyl**	0.02 ± 0.00	0.04 ± 0.03	0.03 ± 0.04	0.06 ± 0.02	0.02 ± 0.01
**500 nm Carboxyl**	0.07 ± 0.02	0.16 ± 0.01	0.08 ± 0.01	0.12 ± 0.05	0.06 ± 0.00
**1000 nm Carboxyl**	0.18 ± 0.03	0.41 ± 0.17	0.17 ± 0.14	0.27 ± 0.06	0.34 ± 0.15
**5000 nm Carboxyl**	n/a	n/a	n/a	n/a	n/a
**50 nm NF**	0.02 ± 0.02	2.60 ± 2.88	0.02 ± 0.02	0.93 ± 0.35	0.10 ± 0.02
**100 nm NF**	0.01 ± 0.02	0.01 ± 0.01	0.02 ± 0.01	0.07 ± 0.01	0.00 ± 0.00
**200 nm NF**	0.02 ± 0.01	0.06 ± 0.04	0.05 ± 0.03	0.05 ± 0.00	0.03 ± 0.04
**500 nm NF**	0.09 ±0.05	0.11 ± 0.02	0.09 ± 0.01	0.09 ± 0.04	0.10 ± 0.01
**1000 nm NF**	0.30 ± 0.11	0.36 ± 0.02	0.18 ± 0.04	0.19 ± 0.08	0.37 ± 0.03
**5000 nm NF**	n/a	n/a	n/a	n/a	n/a

^1^Not available.

Zeta potential of the particles is shown in Table 3. The 50–500 nm aminated particles demonstrated positive zeta potential in the range of +31 to 61 mV in water but the larger 1000 and 5000 nm particles had a net negative charge of about -15 mV. Polystyrene particles are generally prepared by polymerization of styrene monomers induced by an initiator carrying terminal sulfate groups, which populate the particle surface [47]. At neutral pH, the sulfate groups of the initiator covering the particle surface generate negative charges in both functionalized and NF particles [47]. We speculate that the negatively charged sulfate groups on the surface of aminated particles with larger surface areas, *viz* 1000 and 5000 nm particles, may have overwhelmed the positive charges from the amines, giving rise to a net negative charge in the particles. The carboxylated or NF particles exhibited negative zeta potential in the range of -17 to -57 mV in water, which was expected. In RPMI, the surface charges of the aminated particles were attenuated. Salts influence zeta potential due to adsorption of counter ions and formation of a compressed interfacial electric double layer around the particles [48]. Therefore, the change in zeta potential of the particles in RPMI (formulated with HEPES, sodium pyruvate and sodium bicarbonate), was expected. After 24 h incubation at 37 ˚C, 200 and 500 nm aminated particles underwent charge inversion when stored in RPMI and Tween. Similar observation was made by Garg et al., who noticed ‘complex behavior and charge inversion’ of positively charged amidine PS particles in salt solutions [49]. However, the zeta potential of carboxylated and NF particles did not change substantially in RPMI compared to water, except for the strongly charged 5000 nm carboxylated and NF particles. The zeta potential of particles covered with Tween 20, was further lowered. Similar to our observations, Sis and Birinci noticed that non-ionic surfactants (nonylphenol ethoxylates) decreased the ‘absolute magnitude of zeta potential’ in carbon powders [50]. Upon incubation at 37 ˚C for 24 h, no drastic change in zeta potential was noted for most of our particles with, or without, Tween 20. Most aminated particles dispersed in RPMI with or without Tween 20, demonstrated zeta potential between +30 to -30 mV. Formulations with zeta potential values more than 30 mV or lower than -30 mV are considered monodispersed and have high colloidal stability, while those with zeta potential between +5 to -5 mV have a propensity to aggregate/flocculate [51, 52]. Although zeta potential of some particles in RPMI were near neutral in our study, incorporation of Tween 20 most likely prevented particle aggregation through steric repulsion.

**Table 3:** Zeta potential of polystyrene micro/nanoplastics under various conditions (mean ± S.D.; n = 3).

Zeta potential (mV)					
Particle solutions (100 μg/mL)	Water (0 h at RT)	RPMI (0 h at RT)	RPMI + 0.0025% v/v Tween 20 (0 h at RT)	RPMI (stored at 37 ˚C for 24 h)	RPMI + 0.0025% v/v Tween 20 (stored at 37 ˚C for 24 h)
**50 nm Amine**	41.9 ± 3.7	25.8 ± 0.9	7.6 ± 3.4	14.5 ± 5.9	6.3 ± 2.3
**100 nm Amine**	40.3 ± 2.3	14.2 ± 1.2	4.0 ± 2.3	3.3 ± 1.9	1.3 ± 1.1
**200 nm Amine**	31.1 ± 0.7	7.7 ± 0.7	4.1 ± 1.5	-5.2 ± 1.7	-0.64 ± 1.1
**500 nm Amine**	60.5 ± 0.4	11.7 ± 0.3	-1.1 ± 1.0	9.5 ± 0.6	-0.1 ± 0.4
**1000 nm Amine**	-15.1 ± 0.4	-4.5 ± 0.3	-3.3 ± 0.3	-4.9 ± 1.0	-3.9 ± 0.2
**5000 nm Amine**	-14.8 ± 8.6	-5.7 ± 1.6	-3.4 ± 1.4	-5.0 ± 1.0	-3.7 ± 1.0
**50 nm Carboxyl**	-25.1 ± 1.8	-19.9 ± 1.9	-14.3 ± 0.7	-21.0 ± 1.1	-14.8 ± 0.4
**100 nm Carboxyl**	-21.5 ± 1.3	-24.4 ± 2.4	-13.4 ± 1.6	-24.5 ± 4.0	-14.4 ± 1.2
**200 nm Carboxyl**	-28.1 ± 0.2	-27.7 ± 3.5	-20.7 ± 2.7	-30.5 ± 2.5	-20.3 ± 2.4
**500 nm Carboxyl**	-16.9 ± 1.5	-21.2 ± 1.2	-16.8 ± 0.7	-19.9 ± 0.3	-15.6 ± 0.3
**1000 nm Carboxyl**	-23.0 ± 4.5	-17.5 ± 0.7	-13.5 ± 0.1	-20.3 ± 0.3	-12.5 ± 0.1
**5000 nm Carboxyl**	-57.5 ± 3.2	-12.6 ± 2.0	-4.3 ± 1.3	-11.3 ± 0.8	-4.6 ± 1.7
**50 nm NF**	-17.2 ± 5.8	-32.7 ± 2.0	-14.7 ± 3.0	-32.1 ± 2.9	-18.9 ± 1.9
**100 nm NF**	-24.3 ± 0.9	-31.4 ± 2.8	-11.8 ± 4.7	-33.4 ± 1.1	-14.4 ± 2.5
**200 nm NF**	-26.3 ± 0.6	-35.6 ± 1.8	-5.3 ± 1.6	-35.3 ± 3.2	-7.4 ± 3.8
**500 nm NF**	-22.8 ± 2.4	-31.4 ± 2.2	-10.1 ± 0.2	-30.7 ± 0.9	-9.4 ± 0.2
**1000 nm NF**	-30.1 ± 0.3	-29.1 ± 0.8	-10.9 ± 0.3	-33.1 ± 0.1	-10.6 ± 0.4
**5000 nm NF**	-38.3 ± 4.7	-3.5 ± 0.6	-4.9 ± 1.2	-5.5 ± 0.2	-4.6 ± 0.5

### Particle uptake by SNU-1 cells

**Fig 1:**
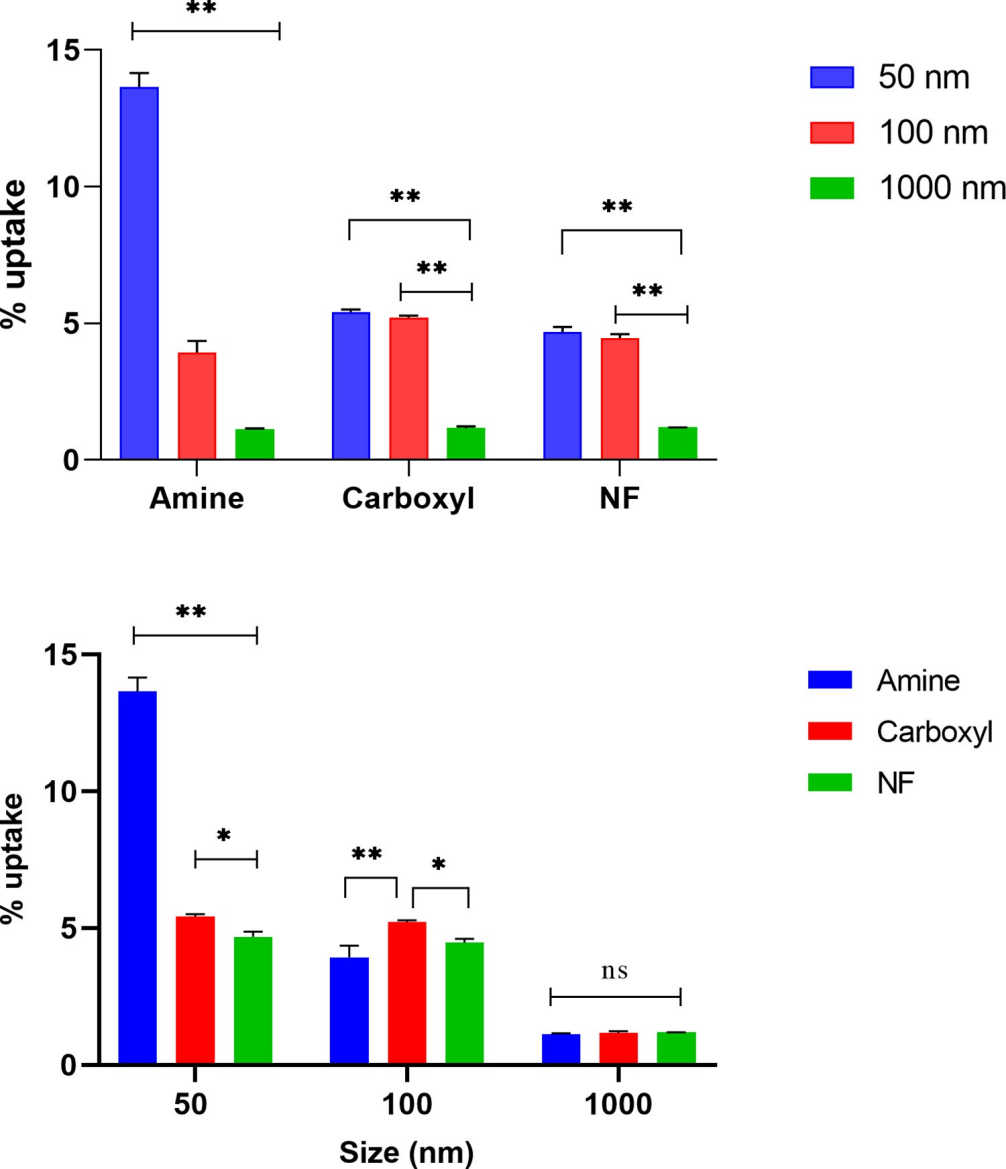
Percent cellular uptake of different sized and surface functionalized polystyrene particles. The uptake of 50, 100 and 1000 nm aminated, carboxylated and NF polystyrene particles by SNU-1 cells were quantitated after 4 h treatment and compared with respect to surface functionalization (A) or particle size (B). All data represented as mean ± S.D. (n = 3). Statistically significant differences are represented by * and ** at p < 0.05, and 0.0001, respectively; ns represents no significant difference.

Size and surface functionalization dependent uptake of PS particles are shown in Fig 1A and 1B. Uptake was dependent on both bead size and surface functionalization (S1 Table). Specifically, cellular uptake of 50 nm aminated particles in 4 h was ~14% and significantly higher than 100 or 1000 nm aminated, carboxylated or NF particles (p<0.0001). In general, the aminated particles, showed a clear trend of size-dependent particle uptake, wherein the 50 nm particles were taken up significantly more than 100 nm particles (~4% p<0.0001), which was in turn higher than the uptake of 1000 nm particles (1.1%, p<0.0001). For carboxylated and NF particles, no size dependent differences in internalization was observed for 50 and 100 nm particles (p>0.05). Internalization of 50 nm carboxylated or NF particles by SNU-1 cells were 5.4 and 4.7%, respectively, while for 100 nm carboxylated or NF particles, the uptake were 3.9 and 4.5%, respectively. In comparison, internalization of 1000 nm carboxylated or NF particles was significantly lower than 50 or 100 nm carboxylated or NF particles (~1.2%, p<0.0001). Amongst particles of the same size but having different surface functionalization, significantly higher uptake was noted for 50 nm aminated particles compared to carboxylated or NF particles (p<0.0001). The 50 nm carboxylated particles were also taken up by the cells more than 50 nm NF particles (p<0.01). Among 100 nm particles, the uptake of 100 nm carboxylated particles were significantly higher than aminated or NF particles of same size (p<0.0001 and p<0.05, respectively). However, uptake of aminated and NF particles was not different. Uptake of 1000 nm particles was similar (P> 0.05) across the three charges. Jiang et al, had also noted higher uptake of carboxylated compared to non-functionalized PS particles (~100 nm) by mesenchymal stem cells [53].

Overall, our study illustrated that particle uptake by SNU-1 cells is dictated by particle size; with substantially higher uptake of 50 or 100 nm particles compared to 1000 nm particles, irrespective of surface functionalization. Also, aminated particles show a greater size-dependent uptake behavior compared to carboxylated or NF particles, which demonstrates surface charge dependent differential uptake characteristics of SNU-1 cells. Higher and rapid uptake of 44 nm compared to 100 nm PS particles has been documented in adenocarcinoma cells [54]. Non-phagocytic cells are generally known to take up 20–50 nm spherical particles at a higher rate compared to other sizes [55]. Higher cellular uptake of cationic particles compared to anionic counterparts has been also documented for gold, silver, iron oxide, chitosan, lipid, and silicon dioxide particles [55]. Yacobi et al. observed 20–40 fold higher flux of 20 and 100–120 nm amidinated particles compared to similar sized carboxylated particles across alveolar epithelial cells [56]. Generally, the cellular uptake mechanism also varies for anionic and cationic particles, wherein anionic particles are internalized mostly through clathrin/caveolae independent endocytosis while cationic particles are taken up via micropinocytosis [57].

**Fig 2:**
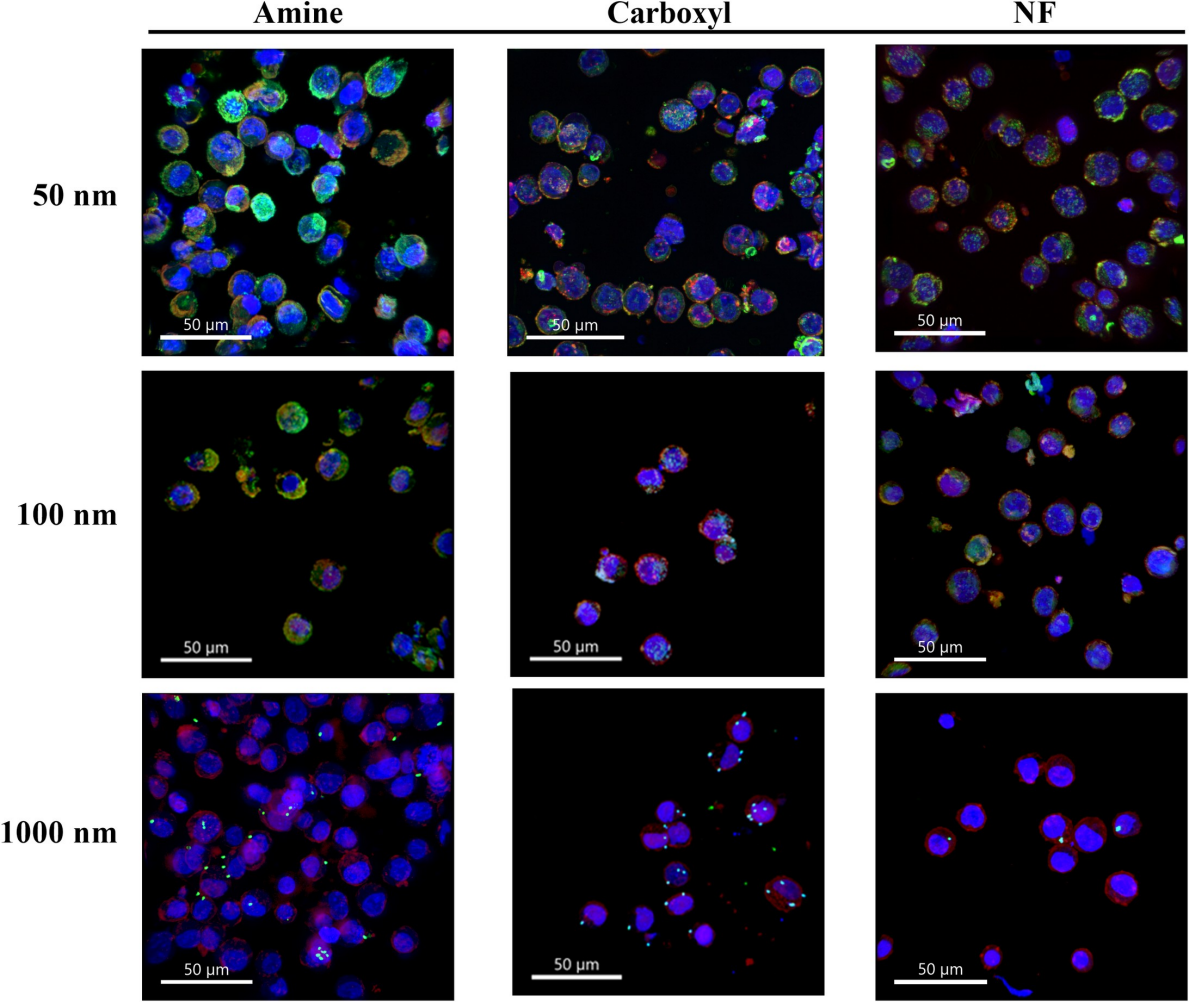
Confocal microscopy images of cells after treatment with polystyrene particles. Representative confocal laser scanning microscopy images of SNU-1 cells after treatment with 50, 100 or 1000 nm aminated, carboxylated or NF particles for 4 h. An overlay of blue representing nuclear stain, red representing cell membrane stain, and green representing MP/NP particles, is provided. All images were taken at 40X magnification and a scale bar of 50 μm was used.

Differential uptake and cellular localization of particles by SNU-1 cells based on their size and surface functionalization was also observed using confocal laser scanning microscopy. As shown in Fig 2 and S3 Fig, regardless of surface functionalization, 50 nm particles were more abundant in the cells compared to 100 nm particles, which in turn were present in higher amounts than 1000 nm particles. The 50 and 100 nm aminated particles appeared to be present mostly in or around the cell membrane, with a smaller proportion in the cytoplasm. Conversely, 50 and 100 nm carboxylated or NF particles appeared to be present in the cytoplasm as well as around the cell membrane. Positive charges on particle surface are known to interact electrostatically with negatively charged phosphate groups in the lipid bilayer of the cell membrane [58]. Significantly lower uptake of 1000 nm particles compared to 50 or 100 nm particles was also observed, corroborating the quantitative uptake data. The 1000 nm particles, irrespective of surface functionalization, were present on the cell surface and not inside the cells. A full panel of confocal microscopy images of the different particle sizes and surface functionalization is provided in S4A, S4B and S4C Fig. None of the particles appeared to be present inside the nucleus or around the nucleus.

### Cellular viability upon treatment with different particles

**Fig 3:**
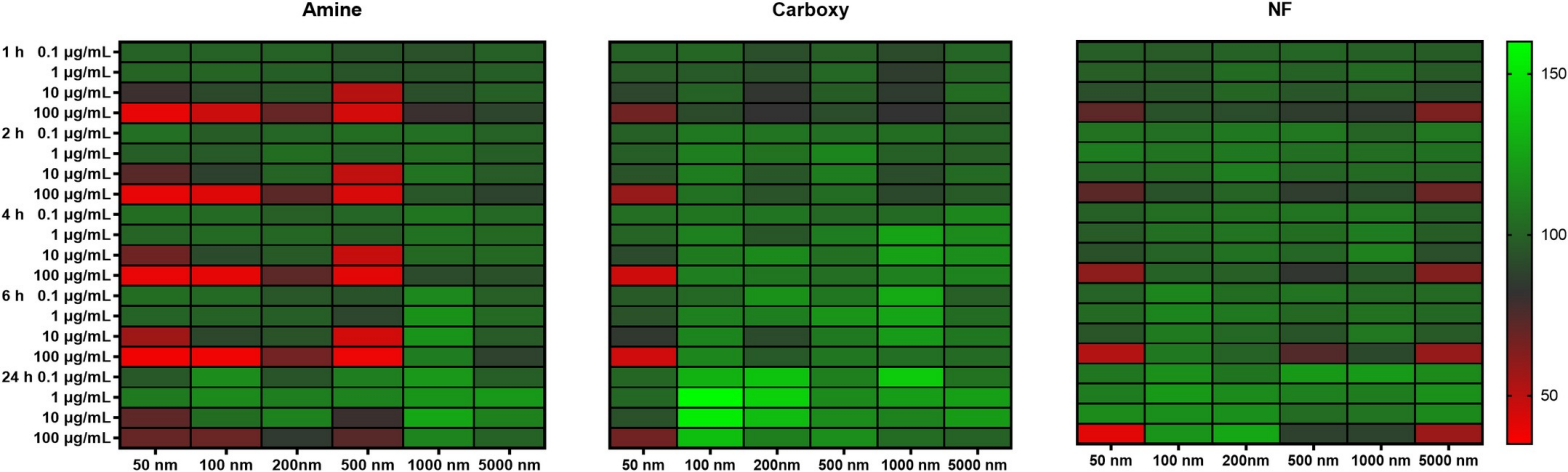
Percentage viability of cells after treatment with polystyrene particles. Heat map representing cytotoxicity of 50–5000 nm aminated, carboxylated or non-functionalized particles after 1, 2, 4, 6 and 24 h exposure of SNU-1 cells to doses of 0.1, 1, 10 and 100 μg/mL. Data are represented as percentage viability, normalized to blank (untreated cells) responses; data range shown is between 35 to 160% (n = 3). Green represents high viability, while red represents low viability.

The viability of cells treated with 50–5000 nm aminated, carboxylated and NF particles for 24 h was investigated using particle concentrations ranging from 0.1–100 μg/mL. As shown in Table 4 and Fig 3, the 50 nm particles were generally more toxic to cells than larger particles. The greatest toxicity was observed within 1 h of treatment with 50 nm aminated particles (≥ 7.5 μg/mL), followed by carboxylated particles (≥ 50 μg/mL) and NF particles (≥ 75 μg/mL). High uptake of 50 nm amine particles compared to carboxylated and NF particles likely contributed to the higher toxicity observed.

**Table 4:** Concentrations of particles that caused significant decreases in the viability of SNU-1 cells compared to untreated cells during 1, 2, 4, 6 or 24 h treatment periods.

Particle type	Size (nm)	Toxic concentration
**Aminated**	50	≥ 7.5 μg/mL at all time points studied
	100	≥ 50 μg/mL at all time points studied
	200	At 100 μg/mL at 1–6 h
	500	≥ 10 μg/mL at all time points studied
	1000	Not toxic up to 100 μg/mL at all time points studied
	5000	At 100 μg/mL at 6 and 24 h
**Carboxylated**	50	≥ 50 μg/mL at 1–6 h
	100	Not toxic up to 100 μg/mL at all time points studied
	200	Not toxic up to 100 μg/mL at all time points studied
	500	Not toxic up to 100 μg/mL at all time points studied
	1000	Not toxic up to 100 μg/mL at all time points studied
	5000	Not toxic up to 100 μg/mL at all time points studied
**Non-functionalized**	50	≥ 75 μg/mL at 1, 4, 6, 24 h time points
	100	Not toxic up to 100 μg/mL at all time points studied
	200	Not toxic up to 100 μg/mL at all time points studied
	500	At 100 μg/mL at 1,2 and 4 h time points
	1000	≥ 75 μg/mL at 1, 2, 4 h time points
	5000	≥ 50 μg/mL at all time points studied

As particle size increased to 100 and 200 nm, the dose of aminated particles required to elicit toxicity increased to ≥50 and 100 μg/mL, respectively. Bhattacharjee et al. similarly noted that 50 nm cationic particles (amine or amidine functionalized) were more toxic than 100 nm cationic particles in NR8383 macrophages [59]. Moreover, all amine functionalized particles in our study, except 1000 nm particles, were more toxic than the carboxylated particles of the same size. The binding of aminated particles to the phospholipid bilayer of the cell membrane, is reported to damage cell membrane integrity, cause deformation and pore formation, resulting in high cellular toxicity [58]. Loos et al. observed that aminated PS particles (~110 nm) inhibited proliferation of THP-1 macrophages but similar sized carboxylated particles had no effect on the proliferation process [60]. Xia et al. observed that 60 nm aminated PS particles were toxic to RAW 264.7 macrophages at 25 μg/mL but not the same sized carboxylated particles [61]. In their study, conversion of the amine functional groups to carboxylates, annulled particle toxicity. Interestingly, in our study, toxicity of 500 nm aminated particles was greater than 100 or 200 nm aminated particles. Although a clear explanation for this observation is not available, it is to be noted that an optimal size range for toxicity has been observed in some studies. For instance, Hesler et al. documented that 500 nm PS particles were more toxic to NIH/3T3 and ES-D3 mouse embryo cells compared to 50 nm particles (half-maximal inhibitory concentrations were 12.6 and >100 μg/mL, respectively) [62].

While cationic particles can cause cell membrane fluidization, anionic particles can cause cell membrane gelation [57, 63]. However, cationic particles are known to cause greater extents of lipid bilayer disruption than anionic particles [64]. Not surprisingly, our carboxylated particles (100–5000 nm) were not toxic to SNU-1 cells at doses up to 100 μg/mL and 6 h treatment. Interestingly, NF particles were toxic at both the smaller and larger end of the size spectrum *i*.*e*. fairly toxic at 50, 1000 and 5000 nm sizes, albeit only at higher doses (50–75 μg/mL). Since zeta potential of carboxylated and non-functionalized particles were similar, we speculate that surface functionalization rather than charge played a greater role in cellular viability.

### Apoptosis induced by different particles

The cellular viability study indicated that toxicity induced by PS particles was based on particle size and surface functionalization. Common mechanisms of cell death are apoptosis or necrosis, wherein apoptosis is programmed cell death triggered by various physical, chemical or biological changes and tightly regulated by the Caspase family of proteases. In contrast, necrosis is unregulated lytic cell death primarily caused by physical injury, pathogens, or chemical insult which often trigger inflammatory responses [65]. Among the Caspase family of enzymes, Caspase-8 is considered the molecular switch for apoptosis and necroptosis/pyroptosis (lytic but programmed inflammatory cell death) [66–68]. Treatment of SNU-1 cells with LPS (10–1000 ng/mL) for 12 h did not trigger release of pro-inflammatory cytokine TNF-α (S5 Fig). Therefore, inflammatory stress related cell death was not further investigated in this study. Lack of an inflammatory response in SNU-1 cells may be attributed to the lack of expression of cyclooxegenease-2, a chief enzyme responsible for causing inflammation [69].

**Fig 4:**
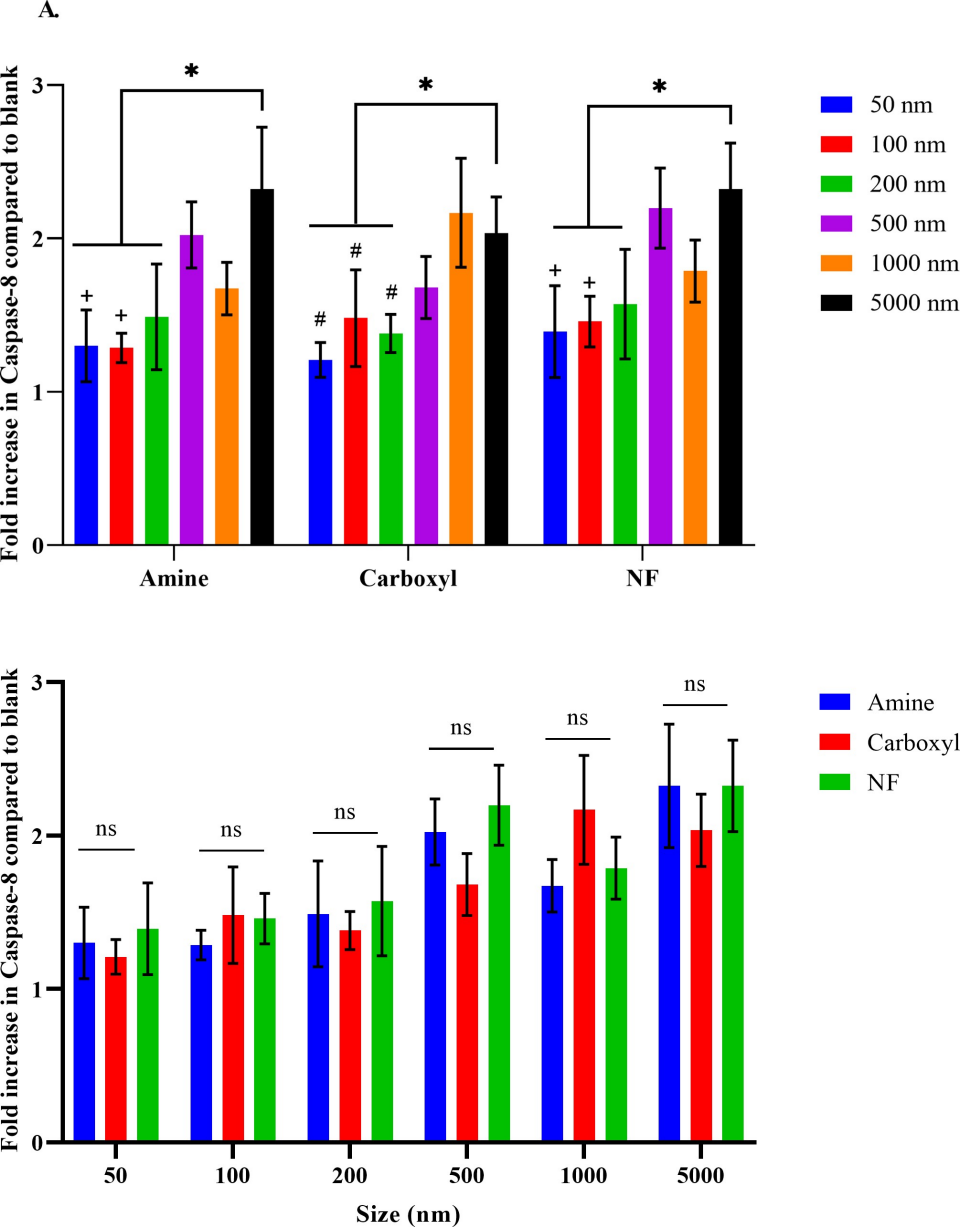
Caspase-8 content in SNU-1 cells. Increases in intracellular Caspase-8 compared to blank (untreated) SNU-1 cells upon treatment with 50–5000 nm sized aminated, carboxylated and NF particles for 4 h. Data compared with respect to surface functionalization (A) or particle size (B). All data represent means ± S.D (n = 4). Statistically significant difference between 5000 nm particles and 50–200 nm particles is represented by * (p<0.05); between 1000 nm particles and 50–100 nm particles as # (p<0.05); and between 500 and 50–100 nm particles as + (p<0.05); ns represents no significant difference.

The potential of PS particles to trigger apoptotic cell death was determined by measuring Caspase-8 concentration, as an early marker for apoptosis. Treatment of SNU-1 cells for 4 h with 50–5000 nm aminated, carboxylated or NF particles increased Caspase-8 levels by 1.2–2.3-fold compared to untreated cells (Fig 4A and 4B). Intracellular Caspase-8 concentration varied based on particle size but not surface functionalization (S2 Table) i.e. a general trend of increase in Caspase-8 levels was observed with increase in particle size but no surface functionalization-based significant difference was observed between same sized particles. The fold increase in Caspase-8 concentration compared to untreated cells were 1.2–1.6 for 50–200 nm particles and 1.7–2.3 for 500–5000 nm particles. Within particles with same surface functionalization, Caspase-8 levels were similar (p>0.05) for cells treated with 50, 100 and 200 nm particles. However, Caspase-8 levels were higher (p<0.05) in 5000 nm treated cells compared to cells treated with 50, 100 or 200 nm particles of the same functionalization; between 1000 nm carboxyl treated cells compared to same surface functionalized 50, 100 and 200 nm treated cells (p<0.05) and between 500 nm amine or NF treated cells compared to same surface functionalized 50 and 100 nm treated cells (p<0.05). The study revealed particle size-based differential accumulation of Caspase-8 in the cells after 4 h of treatment.

In addition to Caspase-8, BAX and Bcl-2 play critical roles in apoptosis progression [70]. Upregulation of BAX causes mitochondrial outer membrane permeabilization leading to apoptotic cell death, while Bcl-2 inactivates BAX, therefore functions as a pro-survival or anti-apoptotic moiety [71–73]. Treatment with 100 μg/mL of 50–5000 nm PS particles (aminated, carboxylated or NF) for 4 h caused significant elevation in BAX concentrations in cells treated with 50 nm amine compared to same surface functionalized 100, 200, 1000 and 5000 nm particles (p<0.05) or compared to 50 nm carboxyl and NF particles (p<0.01) (S6A and S6B Fig, S3 Table). No significant difference was observed between 50 and 500 nm aminated particle treatment, which is consistent with the high toxicity associated with these two particles. Concentrations of BAX were also significantly elevated in cells treated with 5000 nm carboxylated particles compared to 50–200 nm carboxylated particles (p<0.05) and in 5000 nm NF particles treated cells compared to 5000 nm carboxylated/5000 nm aminated/all other NF particle treatments (p<0.0001). A general trend of higher BAX concentrations in 500–5000 nm particle treatments as opposed to 100–200 nm particle treatments was observed, which is consistent with Caspase-8 levels observed in the cells.

Conversely, Bcl-2 concentrations were upregulated only in 50 nm carboxylated particle treated group compared to blank and no significant difference was observed amongst same sized or surface functionalized particles (S7A and S7B Fig, S4 Table). In presence of apoptotic stimuli, Bcl-2 upregulation is critical for cell survival. Therefore, lack of elevation in Bcl-2 expression while increase in BAX and Caspase-8 in cells treated with PS particles, indicate tipping of balance favoring apoptosis onset. González-Fernández et al. had also observed increased transcription of BAX genes, concomitant with decreased gene expression of Bcl-2 in SaB-1 cells exposed to 50 nm aminated PS particle treatment compared to carboxyl or plain PS particles [74].

However, high intracellular pool of Caspase-8 or BAX does not necessarily equate to high apoptotic cell death as activation of downstream signaling cascade is required for the cell’s entry into late-stage apoptosis to cause death. Also, at the 4 h treatment time point, cells treated with smaller particles could potentially be at late-stage apoptosis or undergoing necrotic cell death, where Caspase-8 or BAX concentrations could be low. To investigate this possibility, cells were treated with 50–5000 nm aminated, carboxylated or NF particles for 4 h and intracellular Caspase-3 concentrations were determined. Caspase-3 is often referred to as ‘executioner’ caspase because of its role in the terminal phase of apoptosis *i*.*e*. cleavage of structural, cell cycle and DNase proteins, among other key cellular proteins [75]. Treatment with 50–5000 nm PS particles for 4 h significantly (p<0.01) increased Caspase-3 levels in all the treatments, irrespective of particle size or surface change when compared to blank (1.6–2.4 folds) but no surface charge or particle size specific differences were observed in the Caspase-3 levels (p>0.05) (S8A and S8B Fig, S5 Table).

**Fig 5:**
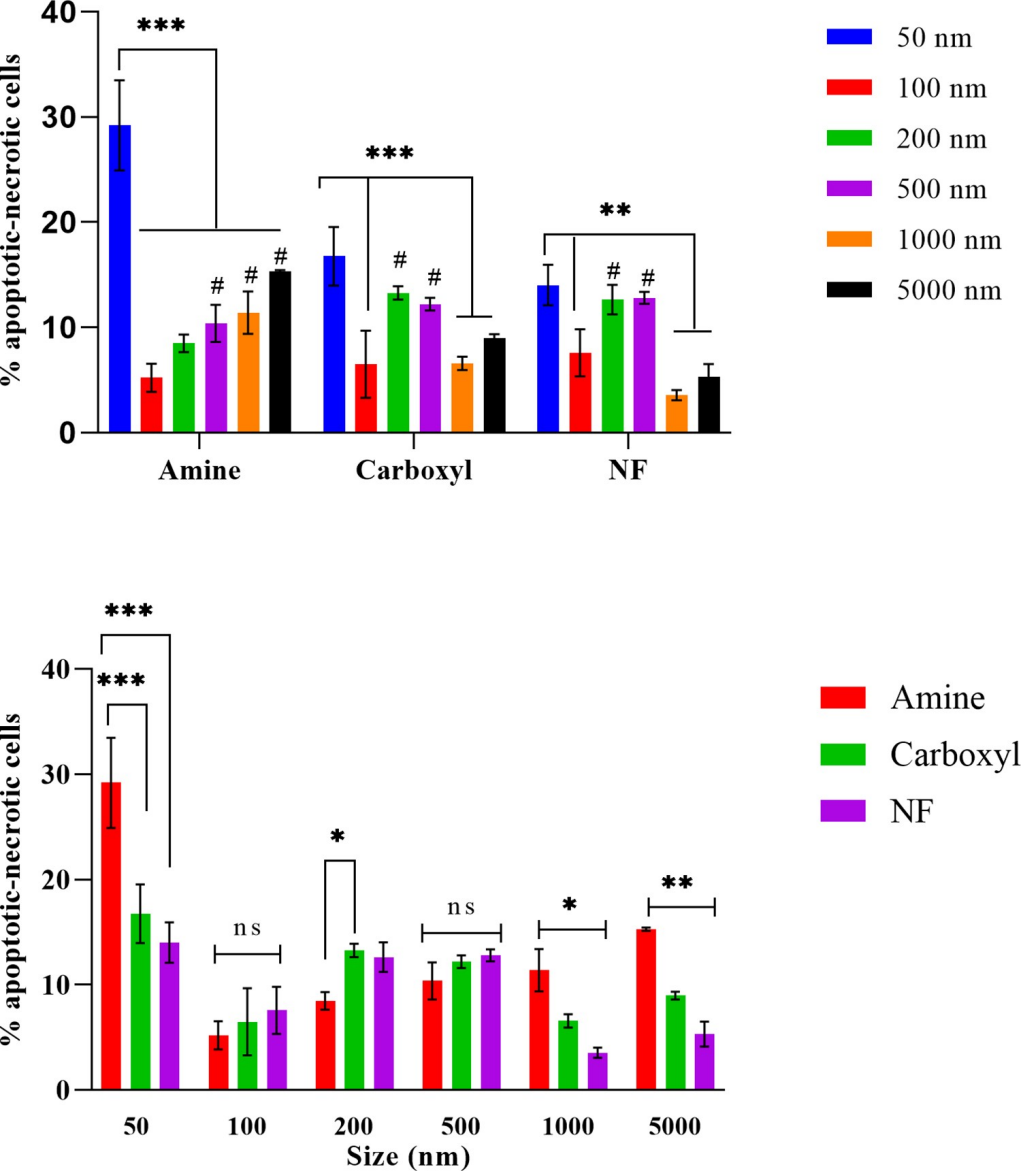
Capability of PS particles in inducing apoptosis-necrosis in SNU-1 cells. Cells were treated for 4 h with 100 μg/mL aminated, carboxylated or NF particles of sizes 50–5000 nm and stained with Annexin V-PI dyes. Plot shows percentage of annexin V-PI positive cells in late apoptosis-necrosis stage. Data compared with respect to surface functionalization (A) or particle size (B). All data represented as mean ± S.D. (n = 4). Statistically significant difference is represented by *, ** and *** at p < 0.05, 0.01 and 0.0001, respectively and between 100 nm compared to same surface functionalized larger sized particles by # (p < 0.05); ns represents no significant difference.

On the contrary, Annexin V-PI study revealed particle size and surface functionalization dependent late-stage apoptosis-necrosis (S6–S8 Tables). Cells were treated with the PS particles for 4 or 24 h and stained with FITC Annexin V and PI, where Annexin V labels apoptotic cells while PI labels necrotic cells. Cell populations exhibiting high Annexin V and PI fluorescence are considered to be in late-stage apoptosis or undergoing necrosis. As expected from viability studies, a high percentage of cells treated for 4 h with 50 nm aminated, carboxylated or NF particles were in late apoptotic and necrotic stages (Fig 5A and 5B, S9A and S9B Fig). Toxicity of 50 nm particles could be attributed to avid cellular uptake of the nanoplastics that could result in high cellular stress, triggering apoptosis-necrosis in a large proportion of the cells. Expectedly, the highest proportion of late apoptotic-necrotic cells after 4 h was in the 50 nm aminated particle treated cell population (29.2%) followed by 50 nm carboxylated (16.8%) and 50 nm NF treated cells (14%). A significant difference was observed in percent apoptosis-necrosis induced by 50 nm aminated particles compared to 50 nm carboxylated or NF particles (p<0.0001). The observation followed the cellular toxicity trend of 50 nm particles: aminated > carboxy > NF. This pattern of late apoptosis-necrosis was also noted for 1000 and 5000 nm particles, albeit at a significantly lower extent compared to 50 nm particles. However, surface functionalization-based difference in late apoptosis-necrosis was not observed in cells treated with 100 and 500 nm particles. Interestingly, the 200 nm amine bead treated cells had significantly lower population of cells in late apoptotic-necrotic stage compared to 200 nm carboxyl bead treated cells (p<0.05). Particle charge based differential induction of apoptosis has been previously demonstrated in three intestinal cell lines, *viz* Caco-2, HT-29 and LS174T using 60 nm amine, carboxyl, and NF PS particles [76]. The cationic particles induced apoptosis in all the cells at 100 μg/mL, but carboxyl or NF particles did not.

Among particles of different sizes but the same surface functionalization, the percentage of late apoptotic-necrotic cells at 4 h was significantly higher in 50 nm particle treated cells compared to 100, 1000 and 5000 nm particle treated cells. On the other hand, the percentage of late apoptotic-necrotic cells were significantly lower in the 100 nm treated group compared to same surface functionalized larger particles viz. 500, 1000, 5000 nm (for amine group) or 200, 500 nm (for carboxyl and NF groups) treated cells. Similar to our observation with 100 nm beads, Sun et al. observed a positive correlation between particle size and apoptosis of NCI-H292 cells exposed to 100, 200 and 300 nm titanium dioxide particles [77]. In our study, a size-dependent trend of increase in percent apoptotic-necrotic cells was noted for 100–5000 nm aminated particles but the carboxylated and NF particles showed a bell-shaped trend, wherein percent apoptotic-necrotic cells were higher for 200 and 500 nm particles compared to 100, 1000 and 5000 nm particles.

After 24 h treatment with particles, the highest percent of apoptotic-necrotic cells was observed in 50 nm carboxylated particle (48.7%) followed by 50 nm NF particle (35.3%) treated cells compared to all other treatments (S10A, S10B, S11A and S11B Figs). Interestingly, only 16.3% cells treated with 50 nm aminated particles were in late-stage apoptosis-necrosis at 24 h. The 50 nm aminated particles likely caused acute toxicity and higher cell death at early hours of exposure compared to 50 nm carboxylated or NF particles. The results suggest that after 24 h exposure to 50 nm amine particles, only a smaller proportion of surviving cells undergo apoptotic-necrotic cell death. On the other hand, longer exposure to 50 nm carboxylated or NF particles significantly increased toxicity. This observation corroborates cell viability results from alamar Blue^®^ toxicity study, wherein the cumulative cell death at 24 h among the 50 nm aminated and carboxylated particles were similar (69.9 and 67.3%, respectively). The percent cells undergoing late-stage apoptosis-necrosis also significantly increased in 24 h in cells treated with 100, 200 nm aminated particles (p<0.01) and 50, 100 nm carboxylated and NF particles (p<0.0001), indicating that toxicity of smaller particles (50–200 nm) can significantly vary based on the length of exposure (S12 Fig and S8 Table). Increase in toxicity with longer exposure to polystyrene particles has been reported by other researchers as well. For instance, Poma et al. documented a decrease in growth of Hs27 cells after 48 h incubation with 75 μg/mL of 100 nm PS particles when compared to 4 or 24 h exposure [78]. Frohlich et al., observed a steep dose dependent decline in viability of EAhy926 cells when exposed to 20 nm carboxylated PS particles for 48–120 h compared to early hours [79]. The group however did not observe this trend with larger (200 nm) PS particles.

## Conclusions

SNU-1 gastric cells were treated with polystyrene particles of varying surface composition and size. Particles across surface functionality and size tended to aggregate in cell culture media that was prevented by inclusion of the surfactant Tween 20. A negative association between particle size and cellular uptake occurred, wherein 50 nm particles were taken up more avidly than 1000 nm particles. The highest uptake was observed for 50 nm amine functionalized particles and confocal microscope images corroborated the uptake results. Cellular uptake of 50 nm particles increased cytotoxicity; the highest being for 50 nm amine functionalized particles after 6 h exposure. In general, aminated and NF particles were more toxic to cells at the two ends of the particle size spectrum (50 and 5000 nm) at doses ≤ 100 μg/mL and ≤ 24 h exposure. Apoptosis-necrosis induced by aminated 50 nm particles in 4 h was greater than all other particles, which is congruent with cellular uptake and viability results. However, 24 h exposure to 50 nm carboxyl and NF particles significantly revved up apoptosis-necrosis in the cells. The study clearly demonstrates that small size (50 nm) is more harmful to gastric cells than large size but positive charges on their surface leads to more acute toxicity compared to negative charges. Overall, the studies show that plastic waste particles should not be considered a mere nuisance but depending on size, dose, surface functionalization, and exposure duration, can be toxic to gastric cells. Among these parameters, particle size seems to play the most critical role in influencing toxicity, while surface functionalization can dictate the onset of toxicity in the cells (acute vs sub-chronic), especially among smaller particles.

## Supporting information

S1 Fig Viability of SNU-1 cells in presence of Tween 20. Toxicity of 0.0025% v/v Tween 20 on SNU-1 cells was determined using alamar Blue assay. No significant difference (ns) was observed in the viability of cells treated with 0.0025% Tween 20 compared to untreated cells (without Tween). Data represented as mean ± S.D. (n = 3).
(TIF)

S2 Fig Scanning electron microscopy images of various particles. Representative electron microscopy images of A) 50–200 nm and B) 500–5000 nm PS particles. Scale bar set at 100 nm for 50–1000 nm particles and at 1000 nm for 5000 nm particles.
(TIF)

S3 Fig Confocal microscopy images of individual cells treated with particles. Representative confocal microscopy images of cells treated with 50, 100 and 1000 nm aminated, carboxylated or NF particles. All images were taken at 40X magnification and scale bar of 20 μm was used to observe individual cells closely.
(TIF)

S4 Fig Confocal microscopy image panels to visualize uptake of different beads by SNU-1 cells. Full panel of representative laser scanning confocal microscopy images of SNU-1 cells after treatment with 50 nm (A), 100 nm (B) or 1000 nm (C) aminated, carboxylated or NF beads for 4 h. All images were taken at 40X magnification and scale bar of 50 μm was used.
(TIF)

S5 Fig Inflammatory response in SNU-1 cells to treatment with various doses of lipopolysaccharide. Secretion of tumor necrosis factor α (TNF-α) after treatment with 0–1000 ng/mL of lipopolysaccharide (LPS) for up to 12 h. Data represented as mean ± S.D. (n = 3). No significant difference between blank (0 ng/mL LPS) treatment and 10–1000 ng/mL LPS treatments is denoted as ns.
(TIF)

S6 Fig BAX concentrations in SNU-1 cells after treatment with particles. Cells were treated with 100 μg/mL of 50–5000 nm PS particles for 4 h. Human BAX concentrations in the cellular lysates were determined and normalized to protein content. Data compared with respect to surface functionalization (A) or particle size (B). All data represented as mean ± S.D. (n = 4). Statistical difference is denoted as *p<0.05, **p<0.01, ***p<0.001, ****p<0.0001 while ns represents no significant difference.
(TIF)

S7 Fig Bcl-2 concentrations in SNU-1 cells after treatment with particles. Cells were treated with 100 μg/mL of 50–5000 nm PS particles for 4 h. Human Bcl-2 concentrations in the cellular lysates were determined and normalized to protein content. Data compared with respect to surface functionalization (A) or particle size (B). All data represented as mean ± S.D. (n = 4). Statistical difference is denoted as ***p<0.001, ****p<0.0001 while ns represents no significant difference.
(TIF)

S8 Fig Caspase-3 content in SNU-1 cells. Increase in intracellular Caspase-3 compared to blank (untreated) SNU-1 cells upon treatment with 50–5000 nm sized aminated, carboxylated and NF particles for 4 h. Data compared with respect to surface functionalization (A) or particle size (B). All data represented as mean ± S.D. (n = 4). No significant difference between the groups is denoted as ns.
(TIF)

S9 Fig Flow cytometer dot plots of apoptosis-necrosis of SNU-1 cells upon treatment with different beads for 4 h. Representative dot plots of Annexin V and PI staining obtained from flow cytometric analyses of cells treated for 4 h with 50–200 nm particles or blank (A) or 500–5000 nm particles (B). The upper right quadrant represents cells at late stage of apoptosis or undergoing necrosis.
(TIF)

S10 Fig Induction of late-stage apoptosis-necrosis in SNU-1 cells by PS particles in 24 h. Percent apoptotic-necrotic cells after 24 h treatment with 100 μg/mL of 50–5000 nm PS particles. Data compared with respect to surface functionalization (A) or particle size (B). All data represented as mean ± S.D. (n = 4). Statistical difference is denoted as **p<0.01, ***p<0.001, ****p<0.0001, # 100 nm compared to other sizes (p<0.05), + 1000 nm compared to 200 & 500 nm (p<0.01), ns represents no significant difference.
(TIF)

S11 Fig Flow cytometer dot plots of apoptosis-necrosis of SNU-1 cells upon treatment with different beads for 24 h. Representative dot plots of Annexin V and PI staining obtained from flow cytometric analyses of cells treated for 24 h with 50–200 nm particles or blank (A) or 500–5000 nm particles (B). The upper right quadrant represents cells at late stage of apoptosis or undergoing necrosis.
(TIF)

S12 Fig Comparison of percent apoptotic-necrotic cells after 4 and 24 h of treatment with PS particles. The figure compares the percent apoptotic-necrotic cells after 4 and 24 h treatment with 100 μg/mL of 50–5000 nm PS particles. All data represented as mean ± S.D. (n = 4). Statistically significant difference between 4 and 24 h is represented as *for aminated (p<0.01), # for carboxylated (<0.0001) and + for NF particles (p<0.0001).
(TIF)

S1 Table Analysis of variance (ANOVA) tables for uptake study. (PDF)

S2 Table ANOVA table for Caspase-8 assay study. (PDF)

S3 Table ANOVA table for BAX assay study. (PDF)

S4 Table ANOVA table for Bcl-2 assay study. (PDF)

S5 Table ANOVA table for Caspase-3 assay study. (PDF)

S6 Table ANOVA tables for Annexin V-PI apoptosis study (4 h). (PDF)

S7 Table ANOVA table for Annexin V-PI apoptosis assay study (24 h). (PDF)

S8 Table ANOVA table comparing Annexin V-PI apoptosis at 4 and 24 h. (PDF)
