# Supplementary figures and images for: Uptake and toxicity of polystyrene micro/nanoplastics in gastric cells: Effects of particle size and surface functionalization
Source: PLoS One. 2021 Dec 31;16(12):e0260803. doi: 10.1371/journal.pone.0260803 (PMC8719689; doi:10.1371/journal.pone.0260803)

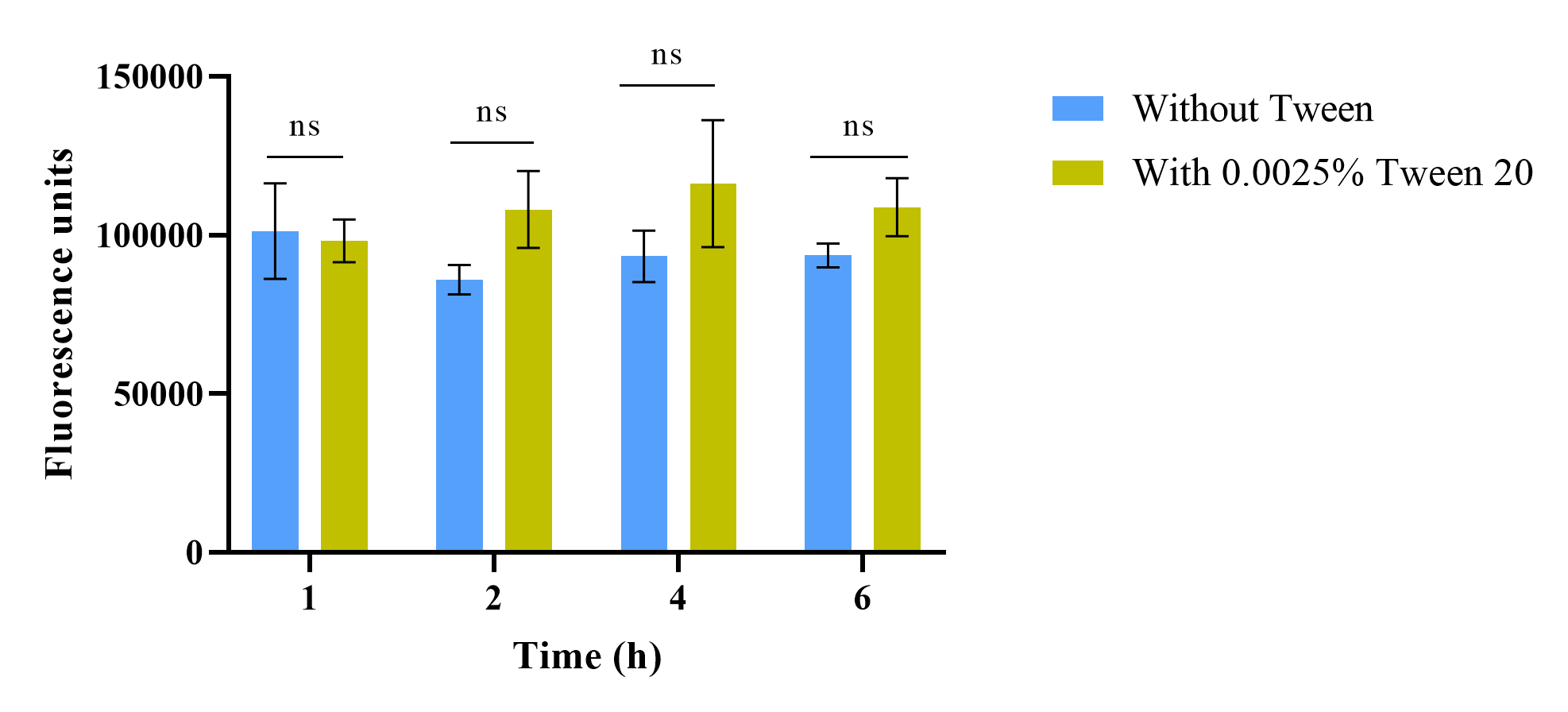

Supplement: S1 Fig — Toxicity of 0.0025% v/v Tween 20 on SNU-1 cells was determined using alamar Blue assay. No significant difference (ns) was observed in the viability of cells treated with 0.0025% Tween 20 compared to untreated cells (without Tween). Data represented as mean ± S.D. (n = 3). (TIF) [file pone.0260803.s001.tif]

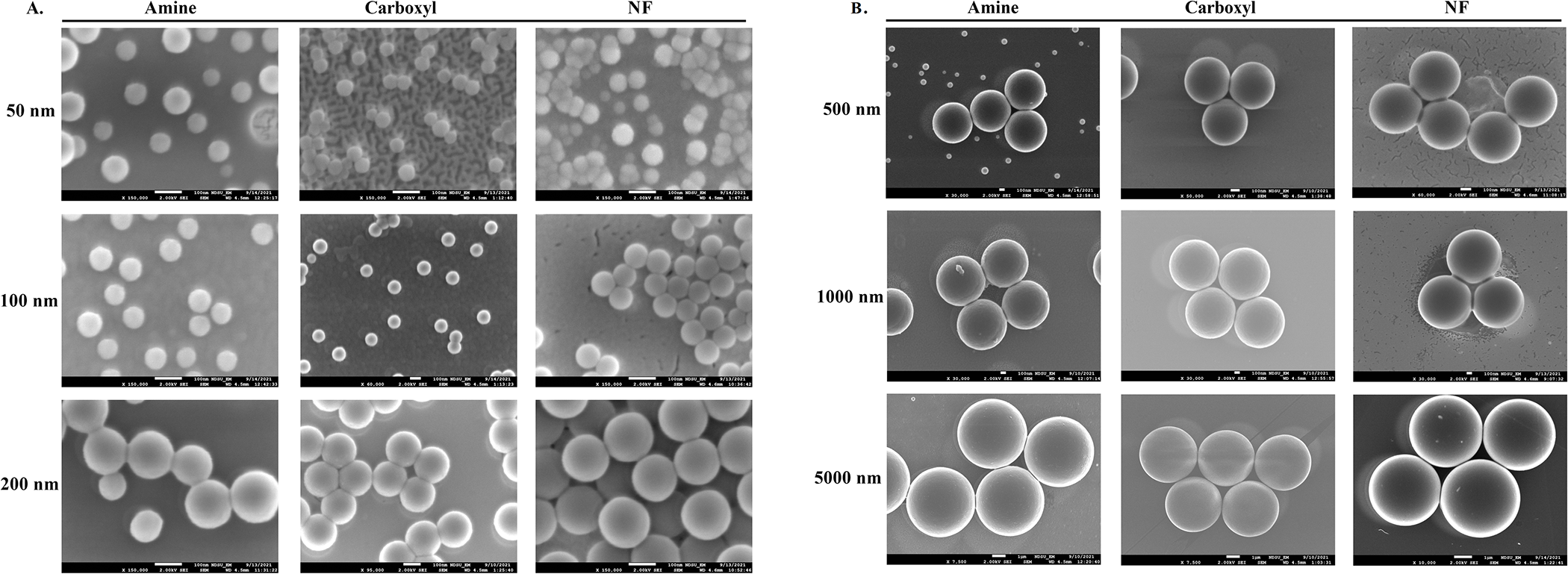

Supplement: S2 Fig — Representative electron microscopy images of A) 50–200 nm and B) 500–5000 nm PS particles. Scale bar set at 100 nm for 50–1000 nm particles and at 1000 nm for 5000 nm particles. (TIF) [file pone.0260803.s002.tif]

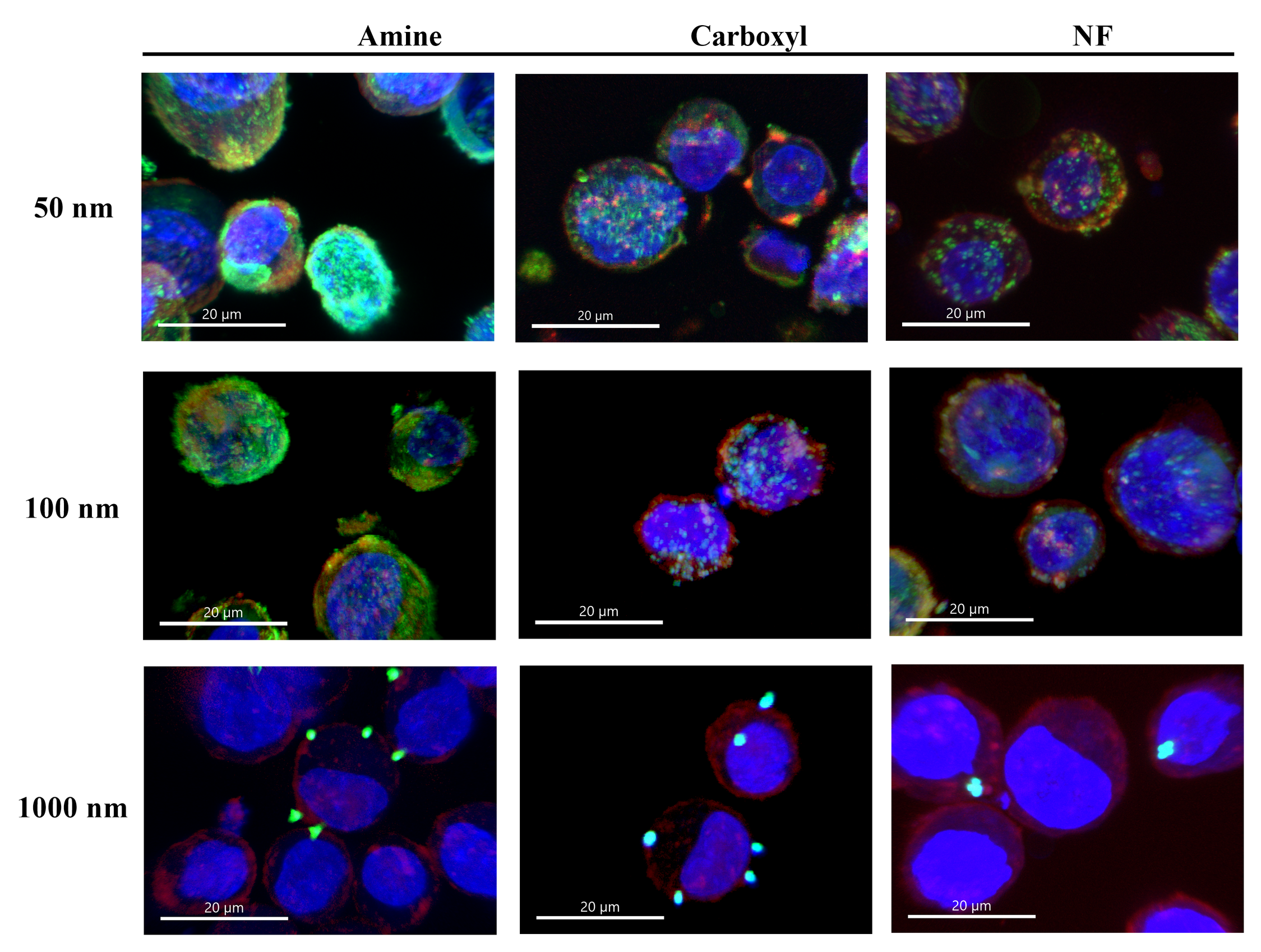

Supplement: S3 Fig — Representative confocal microscopy images of cells treated with 50, 100 and 1000 nm aminated, carboxylated or NF particles. All images were taken at 40X magnification and scale bar of 20 μm was used to observe individual cells closely. (TIF) [file pone.0260803.s003.tif]

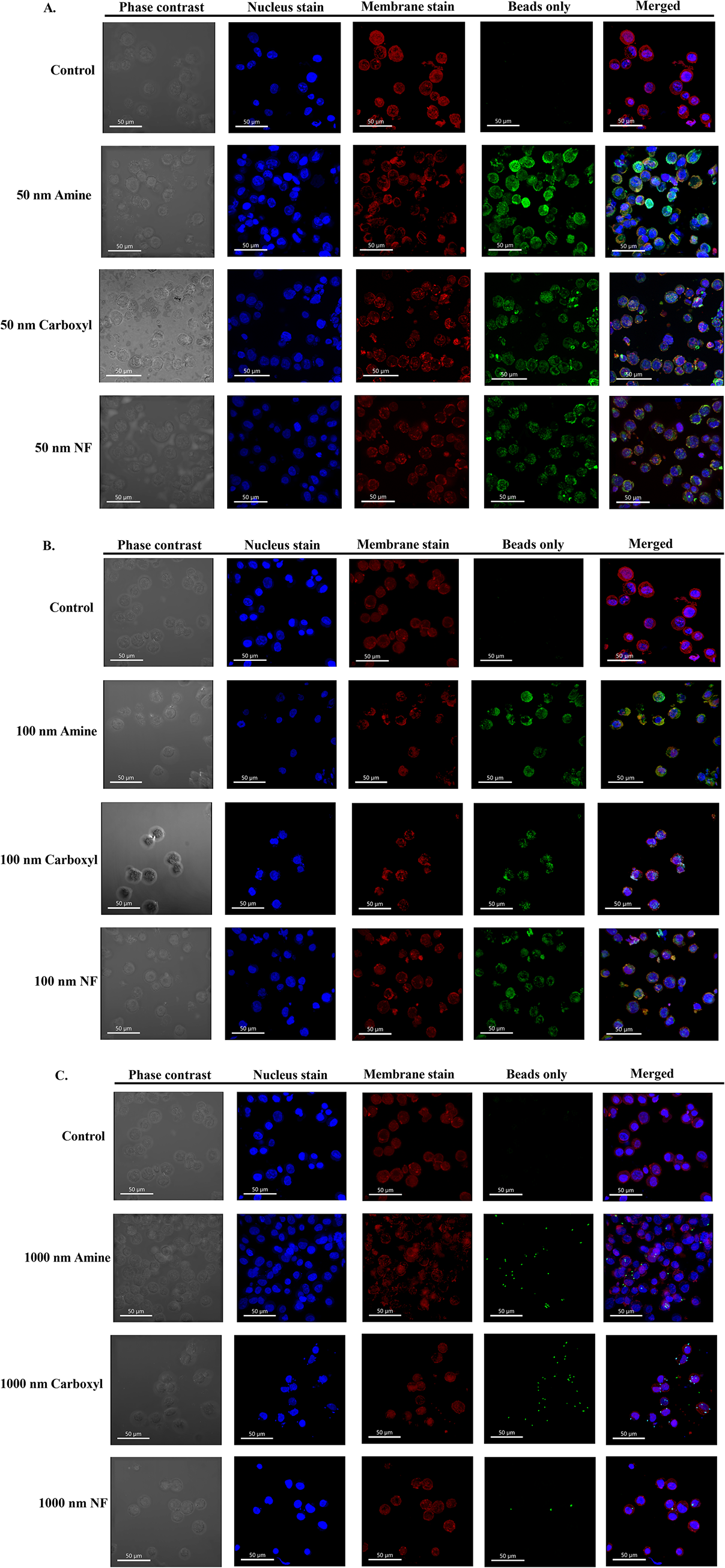

Supplement: S4 Fig — Full panel of representative laser scanning confocal microscopy images of SNU-1 cells after treatment with 50 nm (A), 100 nm (B) or 1000 nm (C) aminated, carboxylated or NF beads for 4 h. All images were taken at 40X magnification and scale bar of 50 μm was used. (TIF) [file pone.0260803.s004.tif]

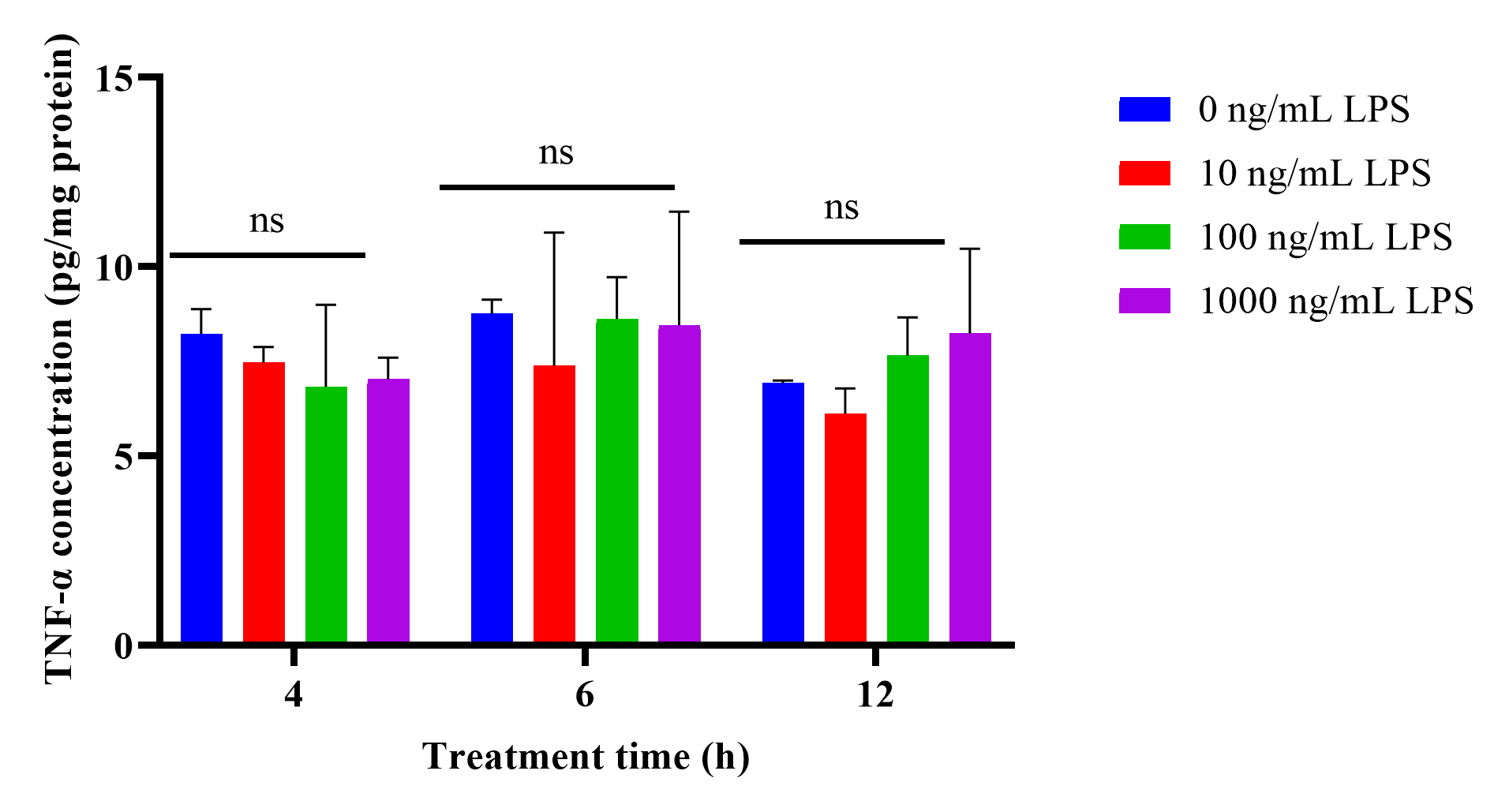

Supplement: S5 Fig — Secretion of tumor necrosis factor α (TNF-α) after treatment with 0–1000 ng/mL of lipopolysaccharide (LPS) for up to 12 h. Data represented as mean ± S.D. (n = 3). No significant difference between blank (0 ng/mL LPS) treatment and 10–1000 ng/mL LPS treatments is denoted as ns. (TIF) [file pone.0260803.s005.tif]

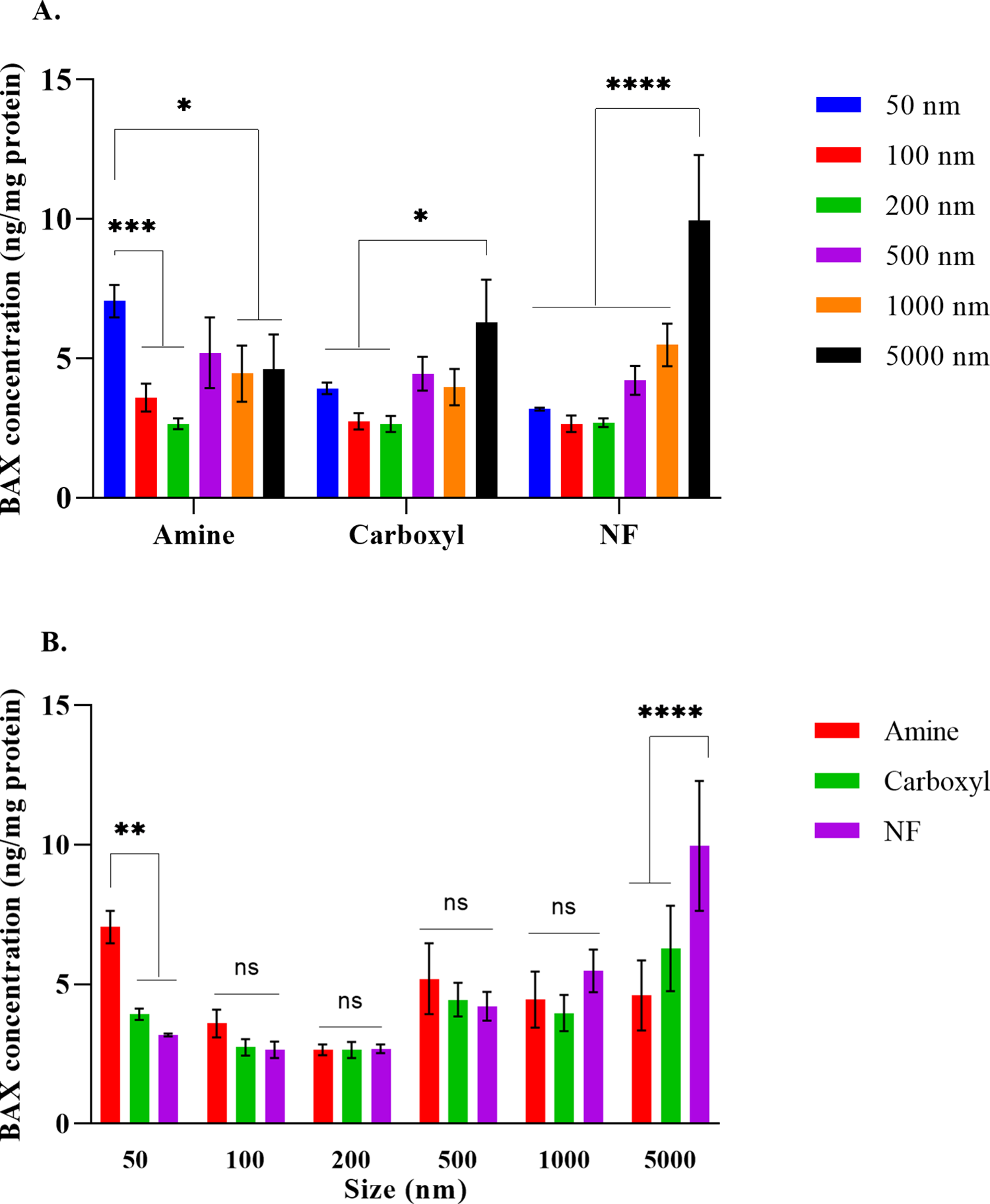

Supplement: S6 Fig — Cells were treated with 100 μg/mL of 50–5000 nm PS particles for 4 h. Human BAX concentrations in the cellular lysates were determined and normalized to protein content. Data compared with respect to surface functionalization (A) or particle size (B). All data represented as mean ± S.D. (n = 4). Statistical difference is denoted as *p<0.05, **p<0.01, ***p<0.001, ****p<0.0001 while ns represents no significant difference. (TIF) [file pone.0260803.s006.tif]

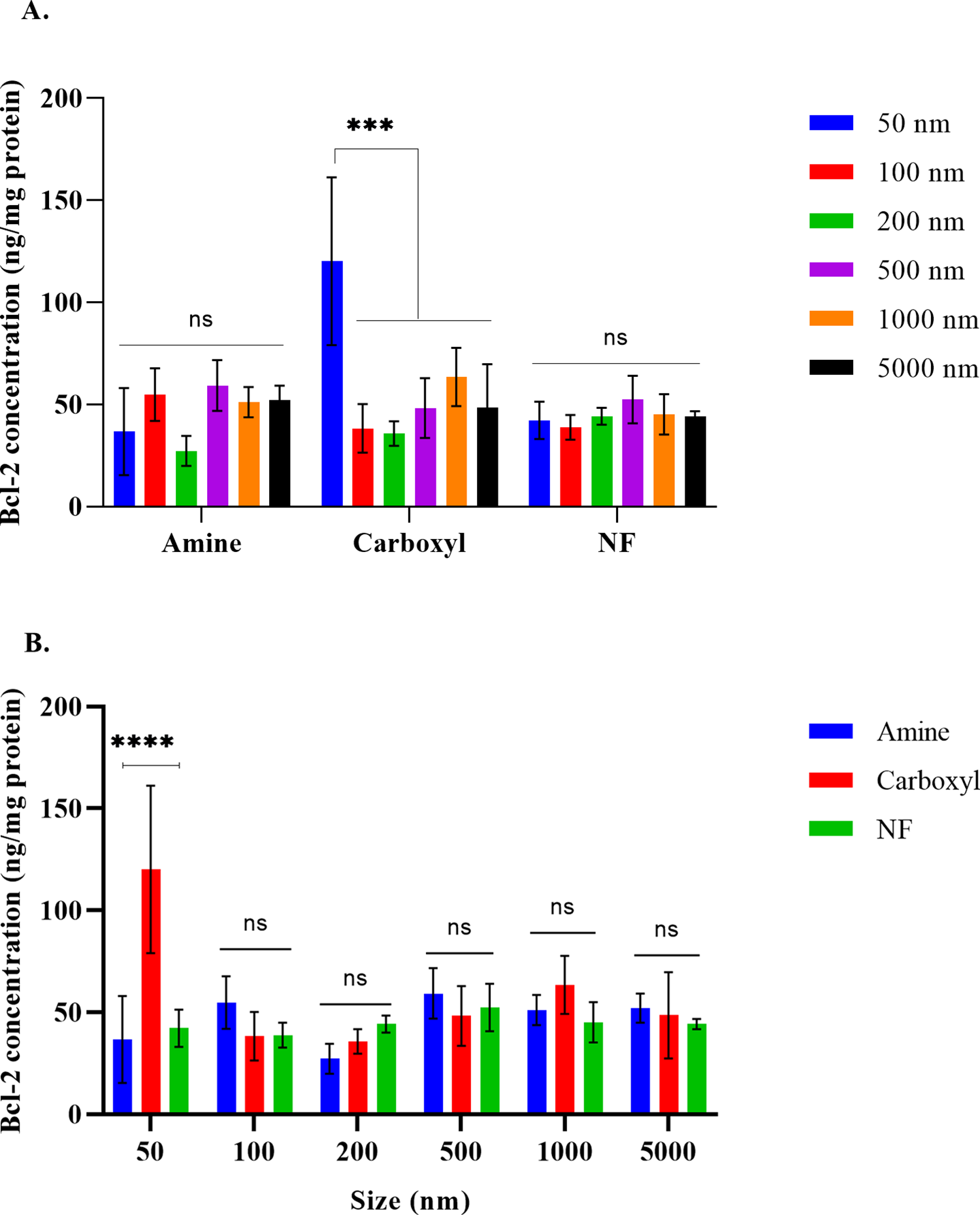

Supplement: S7 Fig — Cells were treated with 100 μg/mL of 50–5000 nm PS particles for 4 h. Human Bcl-2 concentrations in the cellular lysates were determined and normalized to protein content. Data compared with respect to surface functionalization (A) or particle size (B). All data represented as mean ± S.D. (n = 4). Statistical difference is denoted as ***p<0.001, ****p<0.0001 while ns represents no significant difference. (TIF) [file pone.0260803.s007.tif]

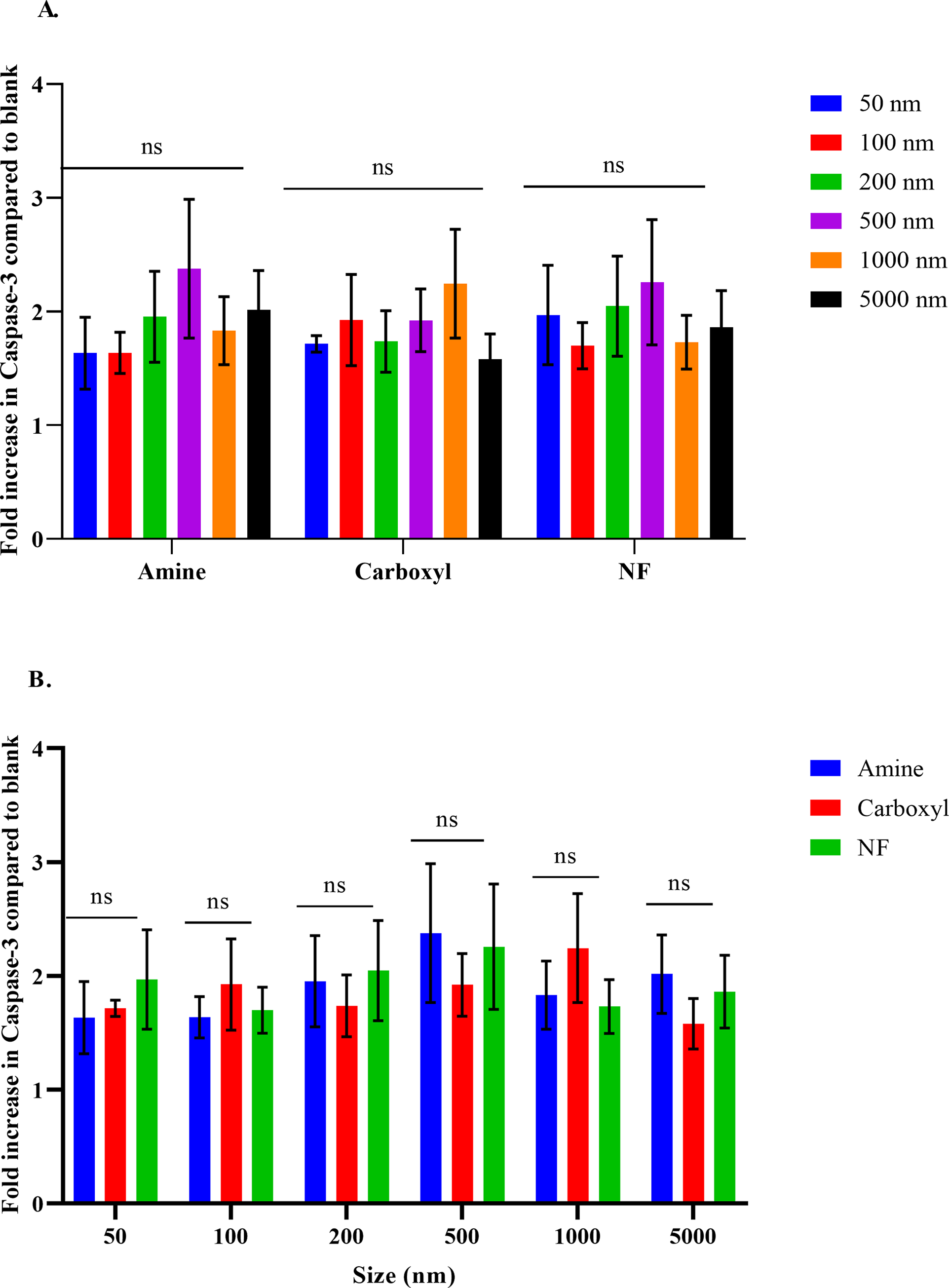

Supplement: S8 Fig — Increase in intracellular Caspase-3 compared to blank (untreated) SNU-1 cells upon treatment with 50–5000 nm sized aminated, carboxylated and NF particles for 4 h. Data compared with respect to surface functionalization (A) or particle size (B). All data represented as mean ± S.D. (n = 4). No significant difference between the groups is denoted as ns. (TIF) [file pone.0260803.s008.tif]

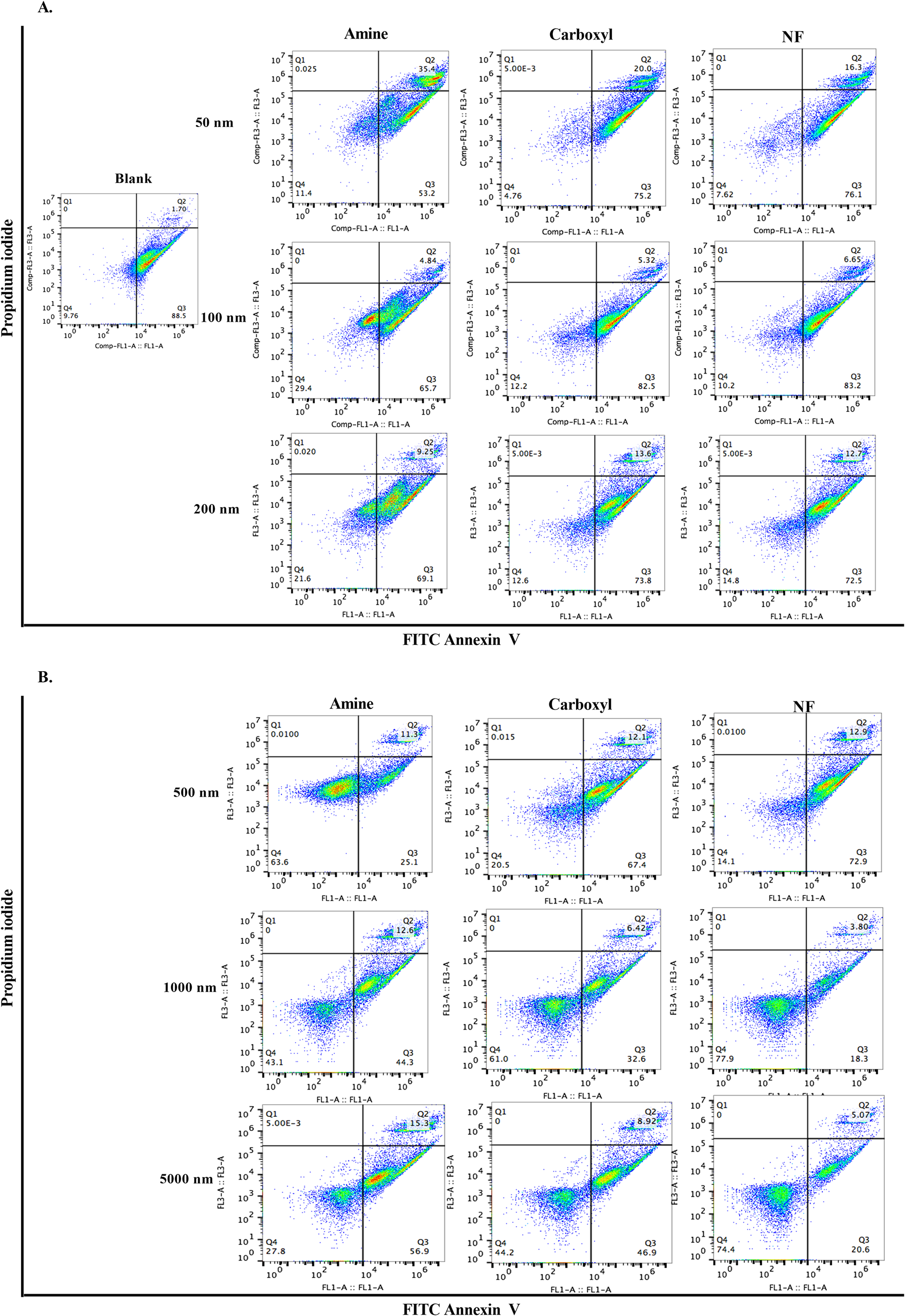

Supplement: S9 Fig — Representative dot plots of Annexin V and PI staining obtained from flow cytometric analyses of cells treated for 4 h with 50–200 nm particles or blank (A) or 500–5000 nm particles (B). The upper right quadrant represents cells at late stage of apoptosis or undergoing necrosis. (TIF) [file pone.0260803.s009.tif]

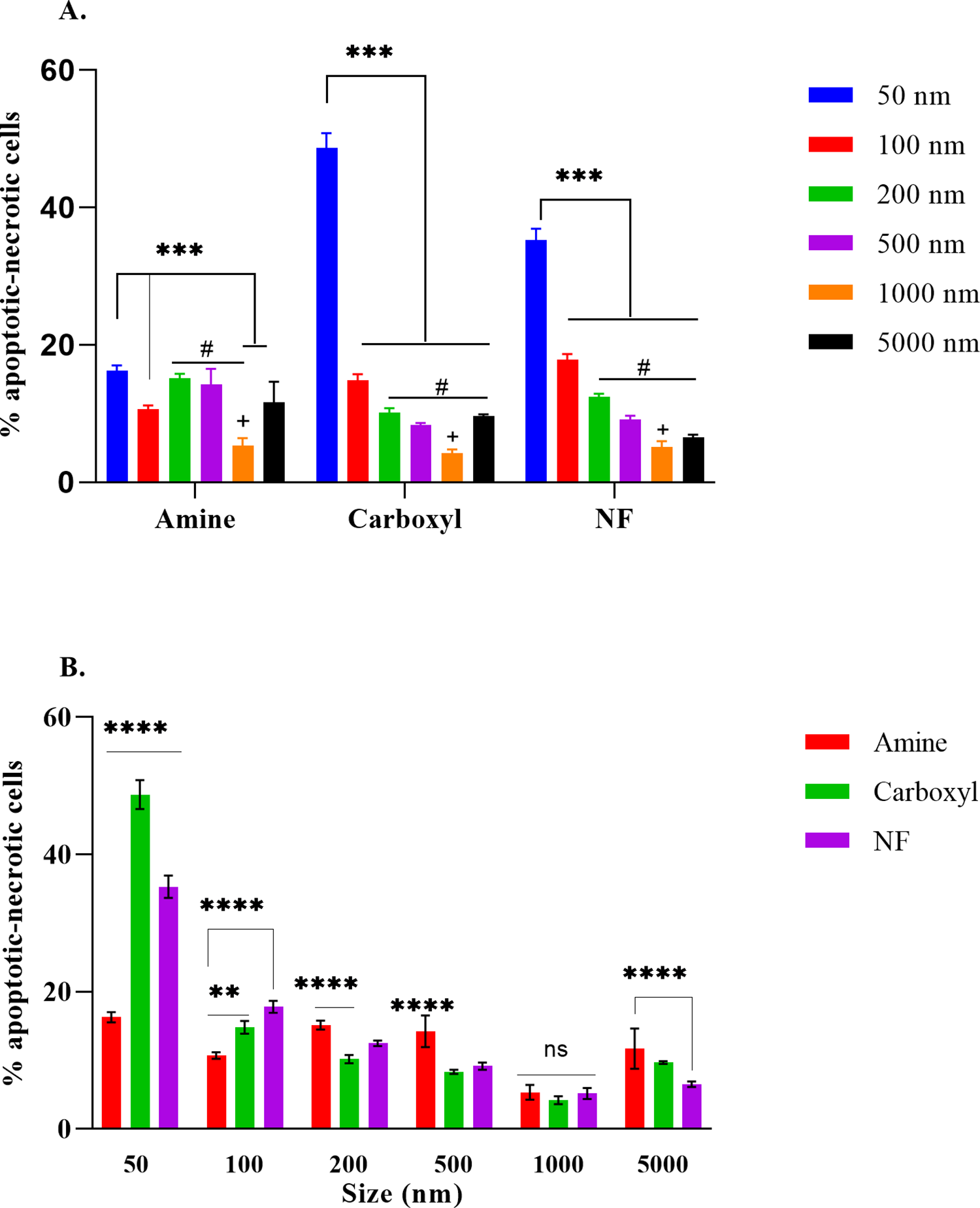

Supplement: S10 Fig — Percent apoptotic-necrotic cells after 24 h treatment with 100 μg/mL of 50–5000 nm PS particles. Data compared with respect to surface functionalization (A) or particle size (B). All data represented as mean ± S.D. (n = 4). Statistical difference is denoted as **p<0.01, ***p<0.001, ****p<0.0001, # 100 nm compared to other sizes (p<0.05), + 1000 nm compared to 200 & 500 nm (p<0.01), ns represents no significant difference. (TIF) [file pone.0260803.s010.tif]

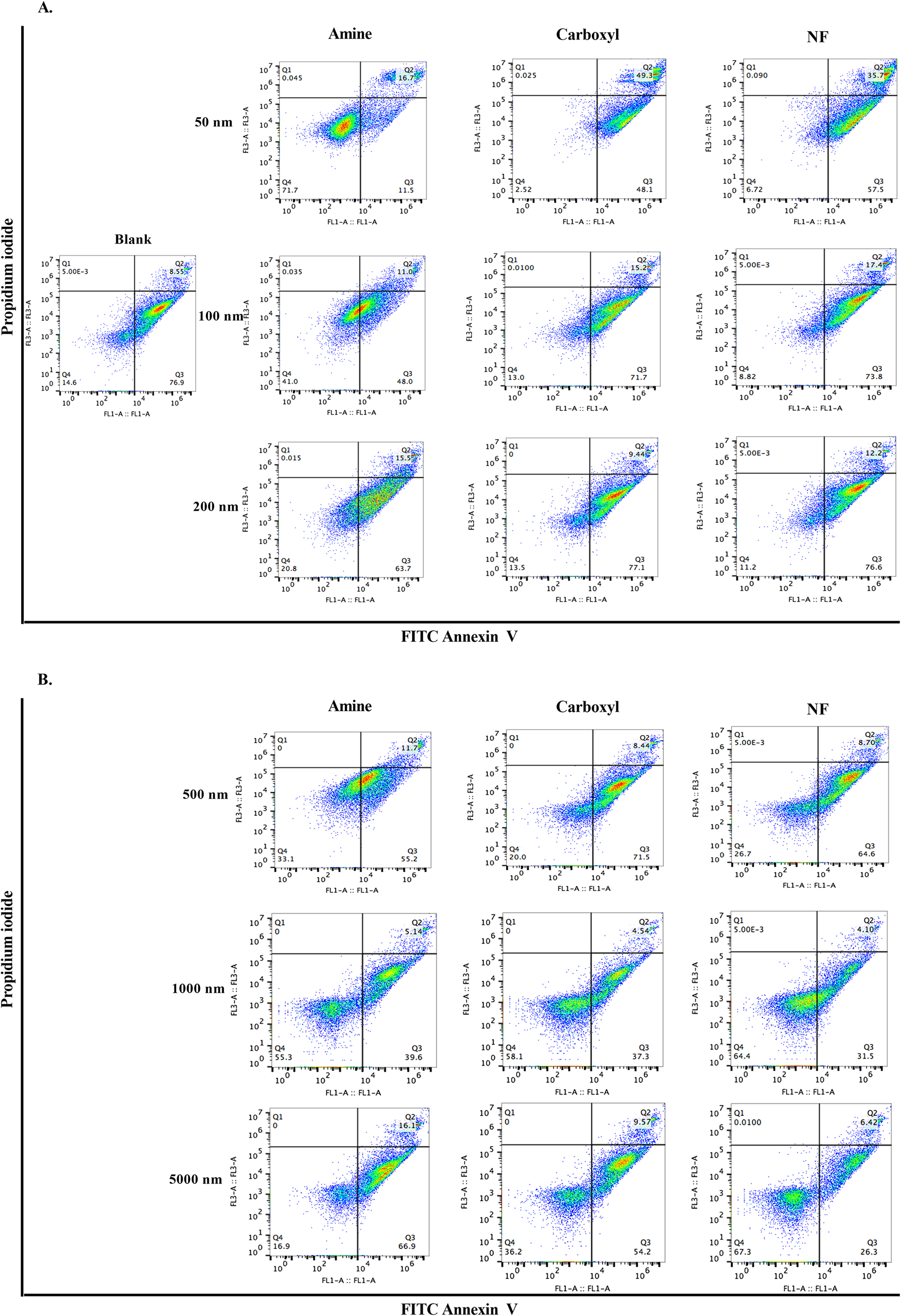

Supplement: S11 Fig — Representative dot plots of Annexin V and PI staining obtained from flow cytometric analyses of cells treated for 24 h with 50–200 nm particles or blank (A) or 500–5000 nm particles (B). The upper right quadrant represents cells at late stage of apoptosis or undergoing necrosis. (TIF) [file pone.0260803.s011.tif]

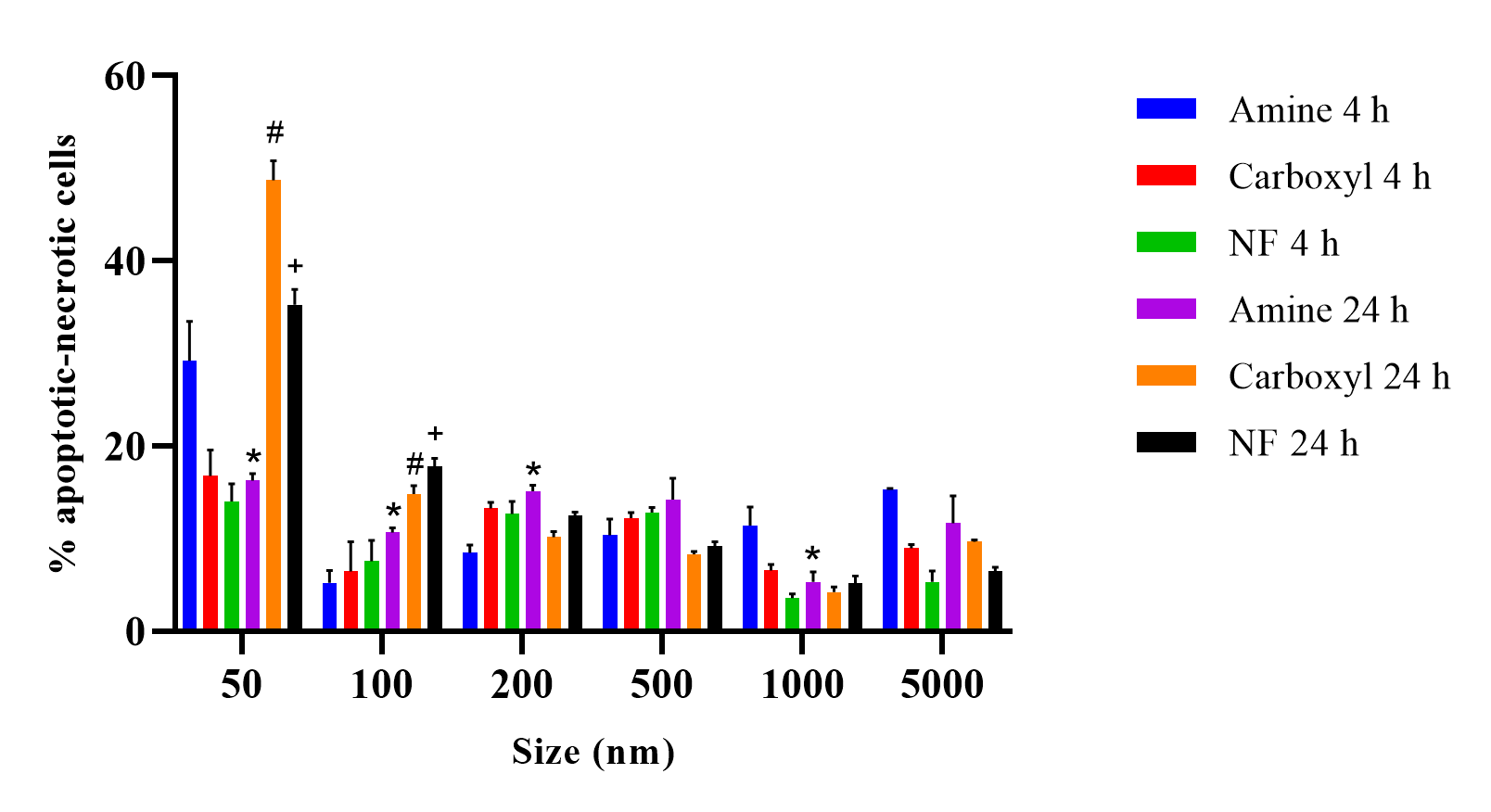

Supplement: S12 Fig — The figure compares the percent apoptotic-necrotic cells after 4 and 24 h treatment with 100 μg/mL of 50–5000 nm PS particles. All data represented as mean ± S.D. (n = 4). Statistically significant difference between 4 and 24 h is represented as *for aminated (p<0.01), # for carboxylated (<0.0001) and + for NF particles (p<0.0001). (TIF) [file pone.0260803.s012.tif]
