# Supplementary material for: Uptake and toxicity of polystyrene micro/nanoplastics in gastric cells: Effects of particle size and surface functionalization
Source: PLoS One. 2021 Dec 31;16(12):e0260803. doi: 10.1371/journal.pone.0260803 (PMC8719689; doi:10.1371/journal.pone.0260803)
Supplement: S1 Table — (PDF) [file pone.0260803.s013.pdf]

Source	Nparm	DF	Sum of Squares	F Ratio	Prob > F
Bead surface functionalization	2	2	39.84132	350.4441	<.0001*
Bead size	2	2	205.09202	1803.989	<.0001*
Bead surface functionalization * Bead size	4	4	110.86174	487.5698	<.0001*

Level	- Level	Difference	Std Err Dif	Lower CL	Upper CL	p-Value
Amine,50 nm	Amine,1000 nm	12.51033	0.1946690	11.8282	13.19243	<.0001*
Amine,50 nm	Carboxyl,1000 nm	12.46300	0.1946690	11.7809	13.14509	<.0001*
Amine,50 nm	NF,1000 nm	12.45000	0.1946690	11.7679	13.13209	<.0001*
Amine,50 nm	Amine,100 nm	9.70433	0.1946690	9.0222	10.38643	<.0001*
Amine,50 nm	NF,100 nm	9.17200	0.1946690	8.4899	9.85409	<.0001*
Amine,50 nm	NF,50 nm	8.95200	0.1946690	8.2699	9.63409	<.0001*
Amine,50 nm	Carboxyl,100 nm	8.42467	0.1946690	7.7426	9.10676	<.0001*
Amine,50 nm	Carboxyl,50 nm	8.21833	0.1946690	7.5362	8.90043	<.0001*
Carboxyl,50 nm	Amine,1000 nm	4.29200	0.1946690	3.6099	4.97409	<.0001*
Carboxyl,50 nm	Carboxyl,1000 nm	4.24467	0.1946690	3.5626	4.92676	<.0001*
Carboxyl,50 nm	NF,1000 nm	4.23167	0.1946690	3.5496	4.91376	<.0001*
Carboxyl,100 nm	Amine,1000 nm	4.08567	0.1946690	3.4036	4.76776	<.0001*
Carboxyl,100 nm	Carboxyl,1000 nm	4.03833	0.1946690	3.3562	4.72043	<.0001*
Carboxyl,100 nm	NF,1000 nm	4.02533	0.1946690	3.3432	4.70743	<.0001*
NF,50 nm	Amine,1000 nm	3.55833	0.1946690	2.8762	4.24043	<.0001*
NF,50 nm	Carboxyl,1000 nm	3.51100	0.1946690	2.8289	4.19309	<.0001*
NF,50 nm	NF,1000 nm	3.49800	0.1946690	2.8159	4.18009	<.0001*
NF,100 nm	Amine,1000 nm	3.33833	0.1946690	2.6562	4.02043	<.0001*
NF,100 nm	Carboxyl,1000 nm	3.29100	0.1946690	2.6089	3.97309	<.0001*
NF,100 nm	NF,1000 nm	3.27800	0.1946690	2.5959	3.96009	<.0001*
Amine,100 nm	Amine,1000 nm	2.80600	0.1946690	2.1239	3.48809	<.0001*
Amine,100 nm	Carboxyl,1000 nm	2.75867	0.1946690	2.0766	3.44076	<.0001*
Amine,100 nm	NF,1000 nm	2.74567	0.1946690	2.0636	3.42776	<.0001*
Carboxyl,50 nm	Amine,100 nm	1.48600	0.1946690	0.8039	2.16809	<.0001*
Carboxyl,100 nm	Amine,100 nm	1.27967	0.1946690	0.5976	1.96176	<.0001*
Carboxyl,50 nm	NF,100 nm	0.95367	0.1946690	0.2716	1.63576	0.0029*
NF,50 nm	Amine,100 nm	0.75233	0.1946690	0.0702	1.43443	0.0244*
Carboxyl,100 nm	NF,100 nm	0.74733	0.1946690	0.0652	1.42943	0.0257*
Carboxyl,50 nm	NF,50 nm	0.73367	0.1946690	0.0516	1.41576	0.0296*
NF,100 nm	Amine,100 nm	0.53233	0.1946690	-0.1498	1.21443	0.2031
Carboxyl,100 nm	NF,50 nm	0.52733	0.1946690	-0.1548	1.20943	0.2118
NF,50 nm	NF,100 nm	0.22000	0.1946690	-0.4621	0.90209	0.9613
Carboxyl,50 nm	Carboxyl,100 nm	0.20633	0.1946690	-0.4758	0.88843	0.9732
NF,1000 nm	Amine,1000 nm	0.06033	0.1946690	-0.6218	0.74243	1.0000
Carboxyl,1000 nm	Amine,1000 nm	0.04733	0.1946690	-0.6348	0.72943	1.0000
NF,1000 nm	Carboxyl,1000 nm	0.01300	0.1946690	-0.6691	0.69509	1.0000
