# Supplementary material for: Uptake and toxicity of polystyrene micro/nanoplastics in gastric cells: Effects of particle size and surface functionalization
Source: PLoS One. 2021 Dec 31;16(12):e0260803. doi: 10.1371/journal.pone.0260803 (PMC8719689; doi:10.1371/journal.pone.0260803)
Supplement: S2 Table — (PDF) [file pone.0260803.s014.pdf]

Source	Nparm	DF	Sum of Squares	F Ratio	Prob > F
Bead surface functionalization	2	2	0.2291785	1.7033	0.1917
Bead size	5	5	8.0695365	23.9903	<.0001*
Bead surface functionalization * Bead size	10	10	1.3145828	1.9541	0.0574

Level	- Level	Difference	Std Err Dif	Lower CL	Upper CL	p-Value
Amine,5000 nm	Carboxyl,50 nm	1.114861	0.1834032	0.443212	1.786510	<.0001*
NF,5000 nm	Carboxyl,50 nm	1.114861	0.1834032	0.443212	1.786510	<.0001*
Amine,5000 nm	Amine,100 nm	1.037083	0.1834032	0.365434	1.708732	<.0001*
NF,5000 nm	Amine,100 nm	1.037083	0.1834032	0.365434	1.708732	<.0001*
Amine,5000 nm	Amine,50 nm	1.023194	0.1834032	0.351545	1.694843	0.0001*
NF,5000 nm	Amine,50 nm	1.023194	0.1834032	0.351545	1.694843	0.0001*
NF,500 nm	Carboxyl,50 nm	0.989385	0.1834032	0.317736	1.661034	0.0002*
Carboxyl,1000 nm	Carboxyl,50 nm	0.958967	0.1834032	0.287318	1.630616	0.0004*
Amine,5000 nm	Carboxyl,200 nm	0.942638	0.1834032	0.270989	1.614287	0.0005*
NF,5000 nm	Carboxyl,200 nm	0.942638	0.1834032	0.270989	1.614287	0.0005*
Amine,5000 nm	NF,50 nm	0.931527	0.1834032	0.259878	1.603176	0.0006*
NF,5000 nm	NF,50 nm	0.931527	0.1834032	0.259878	1.603176	0.0006*
NF,500 nm	Amine,100 nm	0.911608	0.1834032	0.239958	1.583257	0.0009*
NF,500 nm	Amine,50 nm	0.897719	0.1834032	0.226070	1.569368	0.0012*
Carboxyl,1000 nm	Amine,100 nm	0.881189	0.1834032	0.209540	1.552838	0.0016*
Carboxyl,1000 nm	Amine,50 nm	0.867300	0.1834032	0.195651	1.538949	0.0020*
Amine,5000 nm	NF,100 nm	0.864861	0.1834032	0.193212	1.536510	0.0021*
NF,5000 nm	NF,100 nm	0.864861	0.1834032	0.193212	1.536510	0.0021*
Amine,5000 nm	Carboxyl,100 nm	0.842638	0.1834032	0.170989	1.514287	0.0031*
NF,5000 nm	Carboxyl,100 nm	0.842638	0.1834032	0.170989	1.514287	0.0031*
Amine,5000 nm	Amine,200 nm	0.834305	0.1834032	0.162656	1.505954	0.0036*
NF,5000 nm	Amine,200 nm	0.834305	0.1834032	0.162656	1.505954	0.0036*
Carboxyl,5000 nm	Carboxyl,50 nm	0.825887	0.1834032	0.154238	1.497536	0.0042*
NF,500 nm	Carboxyl,200 nm	0.817163	0.1834032	0.145514	1.488812	0.0048*
Amine,500 nm	Carboxyl,50 nm	0.814480	0.1834032	0.142831	1.486129	0.0051*
NF,500 nm	NF,50 nm	0.806052	0.1834032	0.134403	1.477701	0.0059*
Carboxyl,1000 nm	Carboxyl,200 nm	0.786745	0.1834032	0.115096	1.458394	0.0082*
Carboxyl,1000 nm	NF,50 nm	0.775634	0.1834032	0.103985	1.447283	0.0098*
Amine,5000 nm	NF,200 nm	0.750972	0.1834032	0.079323	1.422621	0.0147*
NF,5000 nm	NF,200 nm	0.750972	0.1834032	0.079323	1.422621	0.0147*
Carboxyl,5000 nm	Amine,100 nm	0.748109	0.1834032	0.076460	1.419758	0.0154*
NF,500 nm	NF,100 nm	0.739385	0.1834032	0.067736	1.411034	0.0178*
Amine,500 nm	Amine,100 nm	0.736703	0.1834032	0.065054	1.408352	0.0185*
Carboxyl,5000 nm	Amine,50 nm	0.734221	0.1834032	0.062571	1.405870	0.0193*
Amine,500 nm	Amine,50 nm	0.722814	0.1834032	0.051165	1.394463	0.0231*
NF,500 nm	Carboxyl,100 nm	0.717163	0.1834032	0.045514	1.388812	0.0252*
Carboxyl,1000 nm	NF,100 nm	0.708967	0.1834032	0.037318	1.380616	0.0286*
NF,500 nm	Amine,200 nm	0.708830	0.1834032	0.037181	1.380479	0.0287*
Carboxyl,1000 nm	Carboxyl,100 nm	0.686745	0.1834032	0.015096	1.358394	0.0400*
Carboxyl,1000 nm	Amine,200 nm	0.678411	0.1834032	0.006762	1.350061	0.0453*
Carboxyl,5000 nm	Carboxyl,200 nm	0.653665	0.1834032	-0.017984	1.325314	0.0646
Amine,5000 nm	Amine,1000 nm	0.650190	0.1834032	-0.021459	1.321839	0.0679
NF,5000 nm	Amine,1000 nm	0.650190	0.1834032	-0.021459	1.321839	0.0679
Amine,5000 nm	Carboxyl,500 nm	0.642586	0.1834032	-0.029063	1.314235	0.0754
NF,5000 nm	Carboxyl,500 nm	0.642586	0.1834032	-0.029063	1.314235	0.0754
Carboxyl,5000 nm	NF,50 nm	0.642554	0.1834032	-0.029095	1.314203	0.0755

Level	- Level	Difference	Std Err Dif	Lower CL	Upper CL	p-Value
Amine,500 nm	Carboxyl,200 nm	0.642258	0.1834032	-0.029391	1.313907	0.0758
Amine,500 nm	NF,50 nm	0.631147	0.1834032	-0.040502	1.302796	0.0881
NF,500 nm	NF,200 nm	0.625496	0.1834032	-0.046153	1.297145	0.0950
Carboxyl,1000 nm	NF,200 nm	0.595078	0.1834032	-0.076571	1.266727	0.1404
NF,1000 nm	Carboxyl,50 nm	0.578739	0.1834032	-0.092910	1.250388	0.1711
Carboxyl,5000 nm	NF,100 nm	0.575887	0.1834032	-0.095762	1.247536	0.1770
Amine,500 nm	NF,100 nm	0.564480	0.1834032	-0.107169	1.236129	0.2019
Carboxyl,5000 nm	Carboxyl,100 nm	0.553665	0.1834032	-0.117984	1.225314	0.2277
Carboxyl,5000 nm	Amine,200 nm	0.545332	0.1834032	-0.126317	1.216981	0.2492
Amine,500 nm	Carboxyl,100 nm	0.542258	0.1834032	-0.129391	1.213907	0.2574
Amine,5000 nm	NF,1000 nm	0.536122	0.1834032	-0.135527	1.207771	0.2744
NF,5000 nm	NF,1000 nm	0.536122	0.1834032	-0.135527	1.207771	0.2744
Amine,500 nm	Amine,200 nm	0.533925	0.1834032	-0.137724	1.205574	0.2807
NF,500 nm	Amine,1000 nm	0.524715	0.1834032	-0.146934	1.196364	0.3079
NF,500 nm	Carboxyl,500 nm	0.517110	0.1834032	-0.154539	1.188759	0.3315
NF,1000 nm	Amine,100 nm	0.500961	0.1834032	-0.170688	1.172610	0.3848
Carboxyl,1000 nm	Amine,1000 nm	0.494297	0.1834032	-0.177352	1.165946	0.4080
NF,1000 nm	Amine,50 nm	0.487072	0.1834032	-0.184577	1.158721	0.4337
Carboxyl,1000 nm	Carboxyl,500 nm	0.486692	0.1834032	-0.184957	1.158341	0.4351
Carboxyl,500 nm	Carboxyl,50 nm	0.472275	0.1834032	-0.199374	1.143924	0.4882
Amine,1000 nm	Carboxyl,50 nm	0.464670	0.1834032	-0.206979	1.136320	0.5170
Carboxyl,5000 nm	NF,200 nm	0.461998	0.1834032	-0.209651	1.133647	0.5271
Amine,500 nm	NF,200 nm	0.450591	0.1834032	-0.221058	1.122241	0.5708
NF,500 nm	NF,1000 nm	0.410646	0.1834032	-0.261003	1.082295	0.7199
NF,1000 nm	Carboxyl,200 nm	0.406517	0.1834032	-0.265132	1.078166	0.7344
NF,1000 nm	NF,50 nm	0.395406	0.1834032	-0.276243	1.067055	0.7719
Carboxyl,500 nm	Amine,100 nm	0.394497	0.1834032	-0.277152	1.066146	0.7748
Amine,1000 nm	Amine,100 nm	0.386893	0.1834032	-0.284756	1.058542	0.7990
Carboxyl,500 nm	Amine,50 nm	0.380608	0.1834032	-0.291041	1.052257	0.8179
Carboxyl,1000 nm	NF,1000 nm	0.380228	0.1834032	-0.291421	1.051877	0.8190
Amine,1000 nm	Amine,50 nm	0.373004	0.1834032	-0.298645	1.044653	0.8396
NF,200 nm	Carboxyl,50 nm	0.363889	0.1834032	-0.307760	1.035538	0.8636
Carboxyl,5000 nm	Amine,1000 nm	0.361217	0.1834032	-0.310432	1.032866	0.8703
Carboxyl,5000 nm	Carboxyl,500 nm	0.353612	0.1834032	-0.318037	1.025261	0.8881
Amine,500 nm	Amine,1000 nm	0.349810	0.1834032	-0.321839	1.021459	0.8964
Amine,500 nm	Carboxyl,500 nm	0.342205	0.1834032	-0.329444	1.013854	0.9119
NF,1000 nm	NF,100 nm	0.328739	0.1834032	-0.342910	1.000388	0.9354
NF,1000 nm	Carboxyl,100 nm	0.306517	0.1834032	-0.365132	0.978166	0.9641
Amine,5000 nm	Amine,500 nm	0.300380	0.1834032	-0.371269	0.972029	0.9700
NF,5000 nm	Amine,500 nm	0.300380	0.1834032	-0.371269	0.972029	0.9700
Carboxyl,500 nm	Carboxyl,200 nm	0.300053	0.1834032	-0.371596	0.971702	0.9703
NF,1000 nm	Amine,200 nm	0.298183	0.1834032	-0.373466	0.969832	0.9719
Amine,1000 nm	Carboxyl,200 nm	0.292448	0.1834032	-0.379201	0.964097	0.9765
Amine,5000 nm	Carboxyl,5000 nm	0.288973	0.1834032	-0.382676	0.960622	0.9790
NF,5000 nm	Carboxyl,5000 nm	0.288973	0.1834032	-0.382676	0.960622	0.9790
Carboxyl,500 nm	NF,50 nm	0.288942	0.1834032	-0.382707	0.960591	0.9790
NF,200 nm	Amine,100 nm	0.286111	0.1834032	-0.385538	0.957760	0.9809
Amine,1000 nm	NF,50 nm	0.281337	0.1834032	-0.390312	0.952986	0.9838
Amine,200 nm	Carboxyl,50 nm	0.280556	0.1834032	-0.391093	0.952205	0.9842
NF,200 nm	Amine,50 nm	0.272222	0.1834032	-0.399427	0.943871	0.9883
Carboxyl,100 nm	Carboxyl,50 nm	0.272222	0.1834032	-0.399427	0.943871	0.9883
NF,100 nm	Carboxyl,50 nm	0.250000	0.1834032	-0.421649	0.921649	0.9952
Carboxyl,5000 nm	NF,1000 nm	0.247148	0.1834032	-0.424501	0.918797	0.9958

Level	- Level	Difference	Std Err Dif	Lower CL	Upper CL	p-Value
Amine,500 nm	NF,1000 nm	0.235741	0.1834032	-0.435908	0.907390	0.9975
Carboxyl,500 nm	NF,100 nm	0.222275	0.1834032	-0.449374	0.893924	0.9988
NF,1000 nm	NF,200 nm	0.214850	0.1834032	-0.456799	0.886499	0.9992
Amine,1000 nm	NF,100 nm	0.214670	0.1834032	-0.456979	0.886320	0.9992
Amine,200 nm	Amine,100 nm	0.202778	0.1834032	-0.468871	0.874427	0.9996
Carboxyl,500 nm	Carboxyl,100 nm	0.200053	0.1834032	-0.471596	0.871702	0.9997
Carboxyl,100 nm	Amine,100 nm	0.194444	0.1834032	-0.477205	0.866093	0.9998
Amine,1000 nm	Carboxyl,100 nm	0.192448	0.1834032	-0.479201	0.864097	0.9998
Carboxyl,500 nm	Amine,200 nm	0.191719	0.1834032	-0.479930	0.863369	0.9998
NF,200 nm	Carboxyl,200 nm	0.191667	0.1834032	-0.479982	0.863316	0.9998
Amine,200 nm	Amine,50 nm	0.188889	0.1834032	-0.482760	0.860538	0.9998
Amine,1000 nm	Amine,200 nm	0.184115	0.1834032	-0.487534	0.855764	0.9999
NF,50 nm	Carboxyl,50 nm	0.183333	0.1834032	-0.488316	0.854982	0.9999
Carboxyl,100 nm	Amine,50 nm	0.180556	0.1834032	-0.491093	0.852205	0.9999
NF,200 nm	NF,50 nm	0.180556	0.1834032	-0.491093	0.852205	0.9999
NF,500 nm	Amine,500 nm	0.174905	0.1834032	-0.496744	0.846554	0.9999
Carboxyl,200 nm	Carboxyl,50 nm	0.172222	0.1834032	-0.499427	0.843871	1.0000
NF,100 nm	Amine,100 nm	0.172222	0.1834032	-0.499427	0.843871	1.0000
NF,500 nm	Carboxyl,5000 nm	0.163498	0.1834032	-0.508151	0.835147	1.0000
NF,100 nm	Amine,50 nm	0.158333	0.1834032	-0.513316	0.829982	1.0000
Amine,5000 nm	Carboxyl,1000 nm	0.155894	0.1834032	-0.515756	0.827543	1.0000
NF,5000 nm	Carboxyl,1000 nm	0.155894	0.1834032	-0.515756	0.827543	1.0000
Carboxyl,1000 nm	Amine,500 nm	0.144487	0.1834032	-0.527162	0.816136	1.0000
Carboxyl,1000 nm	Carboxyl,5000 nm	0.133080	0.1834032	-0.538569	0.804729	1.0000
Amine,5000 nm	NF,500 nm	0.125475	0.1834032	-0.546174	0.797124	1.0000
NF,5000 nm	NF,500 nm	0.125475	0.1834032	-0.546174	0.797124	1.0000
NF,1000 nm	Amine,1000 nm	0.114068	0.1834032	-0.557581	0.785717	1.0000
NF,200 nm	NF,100 nm	0.113889	0.1834032	-0.557760	0.785538	1.0000
Carboxyl,500 nm	NF,200 nm	0.108386	0.1834032	-0.563263	0.780035	1.0000
Amine,200 nm	Carboxyl,200 nm	0.108333	0.1834032	-0.563316	0.779982	1.0000
NF,1000 nm	Carboxyl,500 nm	0.106464	0.1834032	-0.565185	0.778113	1.0000
NF,50 nm	Amine,100 nm	0.105556	0.1834032	-0.566093	0.777205	1.0000
Amine,1000 nm	NF,200 nm	0.100782	0.1834032	-0.570867	0.772431	1.0000
Carboxyl,100 nm	Carboxyl,200 nm	0.100000	0.1834032	-0.571649	0.771649	1.0000
Amine,200 nm	NF,50 nm	0.097222	0.1834032	-0.574427	0.768871	1.0000
Carboxyl,200 nm	Amine,100 nm	0.094444	0.1834032	-0.577205	0.766093	1.0000
NF,50 nm	Amine,50 nm	0.091667	0.1834032	-0.579982	0.763316	1.0000
NF,200 nm	Carboxyl,100 nm	0.091667	0.1834032	-0.579982	0.763316	1.0000
Amine,50 nm	Carboxyl,50 nm	0.091667	0.1834032	-0.579982	0.763316	1.0000
Carboxyl,100 nm	NF,50 nm	0.088889	0.1834032	-0.582760	0.760538	1.0000
NF,200 nm	Amine,200 nm	0.083333	0.1834032	-0.588316	0.754982	1.0000
Carboxyl,200 nm	Amine,50 nm	0.080556	0.1834032	-0.591093	0.752205	1.0000
Amine,100 nm	Carboxyl,50 nm	0.077778	0.1834032	-0.593871	0.749427	1.0000
NF,100 nm	Carboxyl,200 nm	0.077778	0.1834032	-0.593871	0.749427	1.0000
NF,100 nm	NF,50 nm	0.066667	0.1834032	-0.604982	0.738316	1.0000
Amine,200 nm	NF,100 nm	0.030556	0.1834032	-0.641093	0.702205	1.0000
NF,500 nm	Carboxyl,1000 nm	0.030418	0.1834032	-0.641231	0.702067	1.0000
Carboxyl,100 nm	NF,100 nm	0.022222	0.1834032	-0.649427	0.693871	1.0000
Amine,50 nm	Amine,100 nm	0.013889	0.1834032	-0.657760	0.685538	1.0000
Carboxyl,5000 nm	Amine,500 nm	0.011407	0.1834032	-0.660242	0.683056	1.0000
NF,50 nm	Carboxyl,200 nm	0.011111	0.1834032	-0.660538	0.682760	1.0000
Amine,200 nm	Carboxyl,100 nm	0.008333	0.1834032	-0.663316	0.679982	1.0000
Carboxyl,500 nm	Amine,1000 nm	0.007605	0.1834032	-0.664044	0.679254	1.0000

Level	- Level	Difference	Std Err Dif	Lower CL	Upper CL	p-Value
NF,5000 nm	Amine,5000 nm	0.000000	0.1834032	-0.671649	0.671649	1.0000
