# Supplementary material for: Uptake and toxicity of polystyrene micro/nanoplastics in gastric cells: Effects of particle size and surface functionalization
Source: PLoS One. 2021 Dec 31;16(12):e0260803. doi: 10.1371/journal.pone.0260803 (PMC8719689; doi:10.1371/journal.pone.0260803)
Supplement: S3 Table — (PDF) [file pone.0260803.s015.pdf]

Tukey's multiple comparisons test	Mean Diff.	95.00% CI of diff.	Below threshold?	Summary	Adjusted P Value
Amine:50 nm vs. Amine:100 nm	3.460	1.128 to 5.792	Yes	***	0.0002
Amine:50 nm vs. Amine:200 nm	4.406	2.074 to 6.738	Yes	****	<0.0001
Amine:50 nm vs. Amine:500 nm	1.853	-0.4786 to 4.185	No	ns	0.2811
Amine:50 nm vs. Amine:1000 nm	2.601	0.2696 to 4.933	Yes	*	0.0151
Amine:50 nm vs. Amine:5000 nm	2.450	0.1183 to 4.782	Yes	*	0.0301
Amine:50 nm vs. Carboxyl:50 nm	3.129	0.7976 to 5.461	Yes	**	0.0011
Amine:50 nm vs. Carboxyl:100 nm	4.313	1.981 to 6.645	Yes	****	<0.0001
Amine:50 nm vs. Carboxyl:200 nm	4.407	2.076 to 6.739	Yes	****	<0.0001
Amine:50 nm vs. Carboxyl:500 nm	2.605	0.2732 to 4.937	Yes	*	0.0149
Amine:50 nm vs. Carboxyl:1000 nm	3.079	0.7472 to 5.411	Yes	**	0.0014
Amine:50 nm vs. Carboxyl:5000 nm	0.7701	-1.562 to 3.102	No	ns	0.9988
Amine:50 nm vs. NF:50 nm	3.871	1.539 to 6.203	Yes	****	<0.0001
Amine:50 nm vs. NF:100 nm	4.403	2.071 to 6.735	Yes	****	<0.0001
Amine:50 nm vs. NF:200 nm	4.366	2.034 to 6.698	Yes	****	<0.0001
Amine:50 nm vs. NF:500 nm	2.837	0.5053 to 5.169	Yes	**	0.0048
Amine:50 nm vs. NF:1000 nm	1.566	-0.7658 to 3.898	No	ns	0.5689
Amine:50 nm vs. NF:5000 nm	-2.910	-5.241 to -0.5778	Yes	**	0.0034
Amine:100 nm vs. Amine:200 nm	0.9459	-1.386 to 3.278	No	ns	0.9882
Amine:100 nm vs. Amine:500 nm	-1.607	-3.939 to 0.7250	No	ns	0.5240
Amine:100 nm vs. Amine:1000 nm	-0.8586	-3.190 to 1.473	No	ns	0.9958
Amine:100 nm vs. Amine:5000 nm	-1.010	-3.342 to 1.322	No	ns	0.9777
Amine:100 nm vs. Carboxyl:50 nm	-0.3307	-2.663 to 2.001	No	ns	>0.9999
Amine:100 nm vs. Carboxyl:100 nm	0.8527	-1.479 to 3.185	No	ns	0.9961
Amine:100 nm vs. Carboxyl:200 nm	0.9474	-1.384 to 3.279	No	ns	0.9880
Amine:100 nm vs. Carboxyl:500 nm	-0.8551	-3.187 to 1.477	No	ns	0.9960
Amine:100 nm vs. Carboxyl:1000 nm	-0.3810	-2.713 to 1.951	No	ns	>0.9999
Amine:100 nm vs. Carboxyl:5000 nm	-2.690	-5.022 to -0.3581	Yes	**	0.0100
Amine:100 nm vs. NF:50 nm	0.4111	-1.921 to 2.743	No	ns	>0.9999
Amine:100 nm vs. NF:100 nm	0.9431	-1.389 to 3.275	No	ns	0.9886
Amine:100 nm vs. NF:200 nm	0.9062	-1.426 to 3.238	No	ns	0.9924
Amine:100 nm vs. NF:500 nm	-0.6229	-2.955 to 1.709	No	ns	>0.9999
Amine:100 nm vs. NF:1000 nm	-1.894	-4.226 to 0.4379	No	ns	0.2487
Amine:100 nm vs. NF:5000 nm	-6.370	-8.702 to -4.038	Yes	****	<0.0001
Amine:200 nm vs. Amine:500 nm	-2.553	-4.885 to -0.2209	Yes	*	0.0190
Amine:200 nm vs. Amine:1000 nm	-1.805	-4.136 to 0.5273	No	ns	0.3231
Amine:200 nm vs. Amine:5000 nm	-1.956	-4.288 to 0.3760	No	ns	0.2045
Amine:200 nm vs. Carboxyl:50 nm	-1.277	-3.608 to 1.055	No	ns	0.8538
Amine:200 nm vs. Carboxyl:100 nm	-0.09320	-2.425 to 2.239	No	ns	>0.9999
Amine:200 nm vs. Carboxyl:200 nm	0.001480	-2.330 to 2.333	No	ns	>0.9999
Amine:200 nm vs. Carboxyl:500 nm	-1.801	-4.133 to 0.5309	No	ns	0.3263
Amine:200 nm vs. Carboxyl:1000 nm	-1.327	-3.659 to 1.005	No	ns	0.8132
Amine:200 nm vs. Carboxyl:5000 nm	-3.636	-5.968 to -1.304	Yes	****	<0.0001
Amine:200 nm vs. NF:50 nm	-0.5349	-2.867 to 1.797	No	ns	>0.9999
Amine:200 nm vs. NF:100 nm	-0.002830	-2.335 to 2.329	No	ns	>0.9999
Amine:200 nm vs. NF:200 nm	-0.03976	-2.372 to 2.292	No	ns	>0.9999
Amine:200 nm vs. NF:500 nm	-1.569	-3.901 to 0.7630	No	ns	0.5658
Amine:200 nm vs. NF:1000 nm	-2.840	-5.172 to -0.5081	Yes	**	0.0048
Amine:200 nm vs. NF:5000 nm	-7.316	-9.647 to -4.984	Yes	****	<0.0001
Amine:500 nm vs. Amine:1000 nm	0.7482	-1.584 to 3.080	No	ns	0.9991
Amine:500 nm vs. Amine:5000 nm	0.5969	-1.735 to 2.929	No	ns	>0.9999
Amine:500 nm vs. Carboxyl:50 nm	1.276	-1.056 to 3.608	No	ns	0.8541
Amine:500 nm vs. Carboxyl:100 nm	2.460	0.1277 to 4.791	Yes	*	0.0288
Amine:500 nm vs. Carboxyl:200 nm	2.554	0.2224 to 4.886	Yes	*	0.0188
Amine:500 nm vs. Carboxyl:500 nm	0.7518	-1.580 to 3.084	No	ns	0.9991
Amine:500 nm vs. Carboxyl:1000 nm	1.226	-1.106 to 3.558	No	ns	0.8893
Amine:500 nm vs. Carboxyl:5000 nm	-1.083	-3.415 to 1.249	No	ns	0.9581

Amine:500 nm vs. NF:50 nm	2.018	-0.3139 to 4.350	No	ns	0.1661
Amine:500 nm vs. NF:100 nm	2.550	0.2181 to 4.882	Yes	*	0.0192
Amine:500 nm vs. NF:200 nm	2.513	0.1812 to 4.845	Yes	*	0.0227
Amine:500 nm vs. NF:500 nm	0.9839	-1.348 to 3.316	No	ns	0.9826
Amine:500 nm vs. NF:1000 nm	-0.2872	-2.619 to 2.045	No	ns	>0.9999
Amine:500 nm vs. NF:5000 nm	-4.763	-7.095 to -2.431	Yes	****	<0.0001
Amine:1000 nm vs. Amine:5000 nm	-0.1513	-2.483 to 2.181	No	ns	>0.9999
Amine:1000 nm vs. Carboxyl:50 nm	0.5279	-1.804 to 2.860	No	ns	>0.9999
Amine:1000 nm vs. Carboxyl:100 nm	1.711	-0.6205 to 4.043	No	ns	0.4128
Amine:1000 nm vs. Carboxyl:200 nm	1.806	-0.5258 to 4.138	No	ns	0.3218
Amine:1000 nm vs. Carboxyl:500 nm	0.003555	-2.328 to 2.335	No	ns	>0.9999
Amine:1000 nm vs. Carboxyl:1000 nm	0.4776	-1.854 to 2.809	No	ns	>0.9999
Amine:1000 nm vs. Carboxyl:5000 nm	-1.831	-4.163 to 0.5005	No	ns	0.2995
Amine:1000 nm vs. NF:50 nm	1.270	-1.062 to 3.602	No	ns	0.8590
Amine:1000 nm vs. NF:100 nm	1.802	-0.5301 to 4.134	No	ns	0.3257
Amine:1000 nm vs. NF:200 nm	1.765	-0.5671 to 4.097	No	ns	0.3600
Amine:1000 nm vs. NF:500 nm	0.2357	-2.096 to 2.568	No	ns	>0.9999
Amine:1000 nm vs. NF:1000 nm	-1.035	-3.367 to 1.296	No	ns	0.9719
Amine:1000 nm vs. NF:5000 nm	-5.511	-7.843 to -3.179	Yes	****	<0.0001
Amine:5000 nm vs. Carboxyl:50 nm	0.6793	-1.653 to 3.011	No	ns	0.9997
Amine:5000 nm vs. Carboxyl:100 nm	1.863	-0.4692 to 4.195	No	ns	0.2733
Amine:5000 nm vs. Carboxyl:200 nm	1.957	-0.3745 to 4.289	No	ns	0.2035
Amine:5000 nm vs. Carboxyl:500 nm	0.1549	-2.177 to 2.487	No	ns	>0.9999
Amine:5000 nm vs. Carboxyl:1000 nm	0.6289	-1.703 to 2.961	No	ns	>0.9999
Amine:5000 nm vs. Carboxyl:5000 nm	-1.680	-4.012 to 0.6518	No	ns	0.4452
Amine:5000 nm vs. NF:50 nm	1.421	-0.9108 to 3.753	No	ns	0.7247
Amine:5000 nm vs. NF:100 nm	1.953	-0.3788 to 4.285	No	ns	0.2063
Amine:5000 nm vs. NF:200 nm	1.916	-0.4157 to 4.248	No	ns	0.2322
Amine:5000 nm vs. NF:500 nm	0.3870	-1.945 to 2.719	No	ns	>0.9999
Amine:5000 nm vs. NF:1000 nm	-0.8841	-3.216 to 1.448	No	ns	0.9942
Amine:5000 nm vs. NF:5000 nm	-5.360	-7.692 to -3.028	Yes	****	<0.0001
Carboxyl:50 nm vs. Carboxyl:100 nm	1.183	-1.148 to 3.515	No	ns	0.9144
Carboxyl:50 nm vs. Carboxyl:200 nm	1.278	-1.054 to 3.610	No	ns	0.8527
Carboxyl:50 nm vs. Carboxyl:500 nm	-0.5244	-2.856 to 1.807	No	ns	>0.9999
Carboxyl:50 nm vs. Carboxyl:1000 nm	-0.05034	-2.382 to 2.282	No	ns	>0.9999
Carboxyl:50 nm vs. Carboxyl:5000 nm	-2.359	-4.691 to -0.02748	Yes	*	0.0445
Carboxyl:50 nm vs. NF:50 nm	0.7417	-1.590 to 3.074	No	ns	0.9992
Carboxyl:50 nm vs. NF:100 nm	1.274	-1.058 to 3.606	No	ns	0.8560
Carboxyl:50 nm vs. NF:200 nm	1.237	-1.095 to 3.569	No	ns	0.8821
Carboxyl:50 nm vs. NF:500 nm	-0.2922	-2.624 to 2.040	No	ns	>0.9999
Carboxyl:50 nm vs. NF:1000 nm	-1.563	-3.895 to 0.7685	No	ns	0.5719
Carboxyl:50 nm vs. NF:5000 nm	-6.039	-8.371 to -3.707	Yes	****	<0.0001
Carboxyl:100 nm vs. Carboxyl:200 nm	0.09468	-2.237 to 2.427	No	ns	>0.9999
Carboxyl:100 nm vs. Carboxyl:500 nm	-1.708	-4.040 to 0.6241	No	ns	0.4165
Carboxyl:100 nm vs. Carboxyl:1000 nm	-1.234	-3.566 to 1.098	No	ns	0.8841
Carboxyl:100 nm vs. Carboxyl:5000 nm	-3.543	-5.875 to -1.211	Yes	***	0.0001
Carboxyl:100 nm vs. NF:50 nm	-0.4417	-2.774 to 1.890	No	ns	>0.9999
Carboxyl:100 nm vs. NF:100 nm	0.09037	-2.241 to 2.422	No	ns	>0.9999
Carboxyl:100 nm vs. NF:200 nm	0.05344	-2.278 to 2.385	No	ns	>0.9999
Carboxyl:100 nm vs. NF:500 nm	-1.476	-3.807 to 0.8562	No	ns	0.6677
Carboxyl:100 nm vs. NF:1000 nm	-2.747	-5.079 to -0.4149	Yes	**	0.0076
Carboxyl:100 nm vs. NF:5000 nm	-7.222	-9.554 to -4.891	Yes	****	<0.0001
Carboxyl:200 nm vs. Carboxyl:500 nm	-1.802	-4.134 to 0.5294	No	ns	0.3250
Carboxyl:200 nm vs. Carboxyl:1000 nm	-1.328	-3.660 to 1.003	No	ns	0.8119
Carboxyl:200 nm vs. Carboxyl:5000 nm	-3.637	-5.969 to -1.306	Yes	****	<0.0001
Carboxyl:200 nm vs. NF:50 nm	-0.5363	-2.868 to 1.796	No	ns	>0.9999
Carboxyl:200 nm vs. NF:100 nm	-0.004310	-2.336 to 2.328	No	ns	>0.9999
Carboxyl:200 nm vs. NF:200 nm	-0.04124	-2.373 to 2.291	No	ns	>0.9999

Carboxyl:200 nm vs. NF:500 nm	-1.570	-3.902 to 0.7615	No	ns	0.5642
Carboxyl:200 nm vs. NF:1000 nm	-2.841	-5.173 to -0.5096	Yes	**	0.0047
Carboxyl:200 nm vs. NF:5000 nm	-7.317	-9.649 to -4.985	Yes	****	<0.0001
Carboxyl:500 nm vs. Carboxyl:1000 nm	0.4740	-1.858 to 2.806	No	ns	>0.9999
Carboxyl:500 nm vs. Carboxyl:5000 nm	-1.835	-4.167 to 0.4969	No	ns	0.2965
Carboxyl:500 nm vs. NF:50 nm	1.266	-1.066 to 3.598	No	ns	0.8616
Carboxyl:500 nm vs. NF:100 nm	1.798	-0.5337 to 4.130	No	ns	0.3289
Carboxyl:500 nm vs. NF:200 nm	1.761	-0.5706 to 4.093	No	ns	0.3634
Carboxyl:500 nm vs. NF:500 nm	0.2322	-2.100 to 2.564	No	ns	>0.9999
Carboxyl:500 nm vs. NF:1000 nm	-1.039	-3.371 to 1.293	No	ns	0.9710
Carboxyl:500 nm vs. NF:5000 nm	-5.515	-7.846 to -3.183	Yes	****	<0.0001
Carboxyl:1000 nm vs. Carboxyl:5000 nm	-2.309	-4.641 to 0.02286	No	ns	0.0550
Carboxyl:1000 nm vs. NF:50 nm	0.7921	-1.540 to 3.124	No	ns	0.9983
Carboxyl:1000 nm vs. NF:100 nm	1.324	-1.008 to 3.656	No	ns	0.8156
Carboxyl:1000 nm vs. NF:200 nm	1.287	-1.045 to 3.619	No	ns	0.8457
Carboxyl:1000 nm vs. NF:500 nm	-0.2419	-2.574 to 2.090	No	ns	>0.9999
Carboxyl:1000 nm vs. NF:1000 nm	-1.513	-3.845 to 0.8189	No	ns	0.6273
Carboxyl:1000 nm vs. NF:5000 nm	-5.989	-8.321 to -3.657	Yes	****	<0.0001
Carboxyl:5000 nm vs. NF:50 nm	3.101	0.7692 to 5.433	Yes	**	0.0013
Carboxyl:5000 nm vs. NF:100 nm	3.633	1.301 to 5.965	Yes	****	<0.0001
Carboxyl:5000 nm vs. NF:200 nm	3.596	1.264 to 5.928	Yes	****	<0.0001
Carboxyl:5000 nm vs. NF:500 nm	2.067	-0.2647 to 4.399	No	ns	0.1399
Carboxyl:5000 nm vs. NF:1000 nm	0.7960	-1.536 to 3.128	No	ns	0.9982
Carboxyl:5000 nm vs. NF:5000 nm	-3.680	-6.012 to -1.348	Yes	****	<0.0001
NF:50 nm vs. NF:100 nm	0.5320	-1.800 to 2.864	No	ns	>0.9999
NF:50 nm vs. NF:200 nm	0.4951	-1.837 to 2.827	No	ns	>0.9999
NF:50 nm vs. NF:500 nm	-1.034	-3.366 to 1.298	No	ns	0.9722
NF:50 nm vs. NF:1000 nm	-2.305	-4.637 to 0.02678	No	ns	0.0559
NF:50 nm vs. NF:5000 nm	-6.781	-9.113 to -4.449	Yes	****	<0.0001
NF:100 nm vs. NF:200 nm	-0.03693	-2.369 to 2.295	No	ns	>0.9999
NF:100 nm vs. NF:500 nm	-1.566	-3.898 to 0.7658	No	ns	0.5690
NF:100 nm vs. NF:1000 nm	-2.837	-5.169 to -0.5053	Yes	**	0.0048
NF:100 nm vs. NF:5000 nm	-7.313	-9.645 to -4.981	Yes	****	<0.0001
NF:200 nm vs. NF:500 nm	-1.529	-3.861 to 0.8028	No	ns	0.6097
NF:200 nm vs. NF:1000 nm	-2.800	-5.132 to -0.4683	Yes	**	0.0058
NF:200 nm vs. NF:5000 nm	-7.276	-9.608 to -4.944	Yes	****	<0.0001
NF:500 nm vs. NF:1000 nm	-1.271	-3.603 to 1.061	No	ns	0.8579
NF:500 nm vs. NF:5000 nm	-5.747	-8.079 to -3.415	Yes	****	<0.0001
NF:1000 nm vs. NF:5000 nm	-4.476	-6.808 to -2.144	Yes	****	<0.0001
