# Supplementary material for: Uptake and toxicity of polystyrene micro/nanoplastics in gastric cells: Effects of particle size and surface functionalization
Source: PLoS One. 2021 Dec 31;16(12):e0260803. doi: 10.1371/journal.pone.0260803 (PMC8719689; doi:10.1371/journal.pone.0260803)
Supplement: S4 Table — (PDF) [file pone.0260803.s016.pdf]

Tukey's multiple comparisons test	Mean Diff.	95.00% CI of diff.	Below threshold?	Summary	Adjusted P Value
Amine:50 nm vs. Amine:100 nm	-18.05	-56.90 to 20.80	No	ns	0.9580
Amine:50 nm vs. Amine:200 nm	9.531	-29.32 to 48.38	No	ns	>0.9999
Amine:50 nm vs. Amine:500 nm	-22.51	-61.36 to 16.34	No	ns	0.7917
Amine:50 nm vs. Amine:1000 nm	-14.38	-53.23 to 24.47	No	ns	0.9955
Amine:50 nm vs. Amine:5000 nm	-15.31	-54.16 to 23.54	No	ns	0.9912
Amine:50 nm vs. Carboxyl:50 nm	-83.40	-122.3 to -44.55	Yes	****	<0.0001
Amine:50 nm vs. Carboxyl:100 nm	-1.500	-40.35 to 37.35	No	ns	>0.9999
Amine:50 nm vs. Carboxyl:200 nm	1.072	-37.78 to 39.92	No	ns	>0.9999
Amine:50 nm vs. Carboxyl:500 nm	-11.44	-50.29 to 27.41	No	ns	0.9997
Amine:50 nm vs. Carboxyl:1000 nm	-26.68	-65.53 to 12.17	No	ns	0.5301
Amine:50 nm vs. Carboxyl:5000 nm	-11.78	-50.63 to 27.07	No	ns	0.9996
Amine:50 nm vs. NF:50 nm	-5.495	-44.35 to 33.36	No	ns	>0.9999
Amine:50 nm vs. NF:100 nm	-1.974	-40.82 to 36.88	No	ns	>0.9999
Amine:50 nm vs. NF:200 nm	-7.389	-46.24 to 31.46	No	ns	>0.9999
Amine:50 nm vs. NF:500 nm	-15.57	-54.42 to 23.28	No	ns	0.9896
Amine:50 nm vs. NF:1000 nm	-8.352	-47.20 to 30.50	No	ns	>0.9999
Amine:50 nm vs. NF:5000 nm	-7.427	-46.28 to 31.42	No	ns	>0.9999
Amine:100 nm vs. Amine:200 nm	27.58	-11.27 to 66.43	No	ns	0.4712
Amine:100 nm vs. Amine:500 nm	-4.463	-43.31 to 34.39	No	ns	>0.9999
Amine:100 nm vs. Amine:1000 nm	3.667	-35.18 to 42.52	No	ns	>0.9999
Amine:100 nm vs. Amine:5000 nm	2.737	-36.11 to 41.59	No	ns	>0.9999
Amine:100 nm vs. Carboxyl:50 nm	-65.35	-104.2 to -26.50	Yes	****	<0.0001
Amine:100 nm vs. Carboxyl:100 nm	16.55	-22.30 to 55.40	No	ns	0.9809
Amine:100 nm vs. Carboxyl:200 nm	19.12	-19.73 to 57.97	No	ns	0.9324
Amine:100 nm vs. Carboxyl:500 nm	6.611	-32.24 to 45.46	No	ns	>0.9999
Amine:100 nm vs. Carboxyl:1000 nm	-8.627	-47.48 to 30.22	No	ns	>0.9999
Amine:100 nm vs. Carboxyl:5000 nm	6.275	-32.58 to 45.13	No	ns	>0.9999
Amine:100 nm vs. NF:50 nm	12.56	-26.29 to 51.41	No	ns	0.9991
Amine:100 nm vs. NF:100 nm	16.08	-22.77 to 54.93	No	ns	0.9856
Amine:100 nm vs. NF:200 nm	10.66	-28.19 to 49.51	No	ns	0.9999
Amine:100 nm vs. NF:500 nm	2.484	-36.37 to 41.33	No	ns	>0.9999
Amine:100 nm vs. NF:1000 nm	9.699	-29.15 to 48.55	No	ns	>0.9999
Amine:100 nm vs. NF:5000 nm	10.62	-28.23 to 49.47	No	ns	0.9999
Amine:200 nm vs. Amine:500 nm	-32.04	-70.90 to 6.805	No	ns	0.2269
Amine:200 nm vs. Amine:1000 nm	-23.92	-62.77 to 14.94	No	ns	0.7100
Amine:200 nm vs. Amine:5000 nm	-24.84	-63.69 to 14.01	No	ns	0.6510
Amine:200 nm vs. Carboxyl:50 nm	-92.93	-131.8 to -54.08	Yes	****	<0.0001
Amine:200 nm vs. Carboxyl:100 nm	-11.03	-49.88 to 27.82	No	ns	0.9998
Amine:200 nm vs. Carboxyl:200 nm	-8.458	-47.31 to 30.39	No	ns	>0.9999
Amine:200 nm vs. Carboxyl:500 nm	-20.97	-59.82 to 17.88	No	ns	0.8670
Amine:200 nm vs. Carboxyl:1000 nm	-36.21	-75.06 to 2.641	No	ns	0.0944
Amine:200 nm vs. Carboxyl:5000 nm	-21.31	-60.16 to 17.54	No	ns	0.8521
Amine:200 nm vs. NF:50 nm	-15.03	-53.88 to 23.82	No	ns	0.9928
Amine:200 nm vs. NF:100 nm	-11.50	-50.36 to 27.35	No	ns	0.9997
Amine:200 nm vs. NF:200 nm	-16.92	-55.77 to 21.93	No	ns	0.9765
Amine:200 nm vs. NF:500 nm	-25.10	-63.95 to 13.75	No	ns	0.6345
Amine:200 nm vs. NF:1000 nm	-17.88	-56.73 to 20.97	No	ns	0.9613
Amine:200 nm vs. NF:5000 nm	-16.96	-55.81 to 21.89	No	ns	0.9760
Amine:500 nm vs. Amine:1000 nm	8.130	-30.72 to 46.98	No	ns	>0.9999
Amine:500 nm vs. Amine:5000 nm	7.201	-31.65 to 46.05	No	ns	>0.9999
Amine:500 nm vs. Carboxyl:50 nm	-60.89	-99.74 to -22.04	Yes	****	<0.0001
Amine:500 nm vs. Carboxyl:100 nm	21.01	-17.84 to 59.86	No	ns	0.8651
Amine:500 nm vs. Carboxyl:200 nm	23.59	-15.26 to 62.44	No	ns	0.7301
Amine:500 nm vs. Carboxyl:500 nm	11.07	-27.78 to 49.92	No	ns	0.9998
Amine:500 nm vs. Carboxyl:1000 nm	-4.164	-43.01 to 34.69	No	ns	>0.9999
Amine:500 nm vs. Carboxyl:5000 nm	10.74	-28.11 to 49.59	No	ns	0.9999

Amine:500 nm vs. NF:50 nm	17.02	-21.83 to 55.87	No	ns	0.9752
Amine:500 nm vs. NF:100 nm	20.54	-18.31 to 59.39	No	ns	0.8847
Amine:500 nm vs. NF:200 nm	15.13	-23.73 to 53.98	No	ns	0.9923
Amine:500 nm vs. NF:500 nm	6.947	-31.90 to 45.80	No	ns	>0.9999
Amine:500 nm vs. NF:1000 nm	14.16	-24.69 to 53.01	No	ns	0.9962
Amine:500 nm vs. NF:5000 nm	15.09	-23.76 to 53.94	No	ns	0.9925
Amine:1000 nm vs. Amine:5000 nm	-0.9291	-39.78 to 37.92	No	ns	>0.9999
Amine:1000 nm vs. Carboxyl:50 nm	-69.02	-107.9 to -30.17	Yes	****	<0.0001
Amine:1000 nm vs. Carboxyl:100 nm	12.88	-25.97 to 51.73	No	ns	0.9987
Amine:1000 nm vs. Carboxyl:200 nm	15.46	-23.39 to 54.31	No	ns	0.9903
Amine:1000 nm vs. Carboxyl:500 nm	2.944	-35.91 to 41.79	No	ns	>0.9999
Amine:1000 nm vs. Carboxyl:1000 nm	-12.29	-51.14 to 26.56	No	ns	0.9993
Amine:1000 nm vs. Carboxyl:5000 nm	2.608	-36.24 to 41.46	No	ns	>0.9999
Amine:1000 nm vs. NF:50 nm	8.889	-29.96 to 47.74	No	ns	>0.9999
Amine:1000 nm vs. NF:100 nm	12.41	-26.44 to 51.26	No	ns	0.9992
Amine:1000 nm vs. NF:200 nm	6.996	-31.85 to 45.85	No	ns	>0.9999
Amine:1000 nm vs. NF:500 nm	-1.183	-40.03 to 37.67	No	ns	>0.9999
Amine:1000 nm vs. NF:1000 nm	6.033	-32.82 to 44.88	No	ns	>0.9999
Amine:1000 nm vs. NF:5000 nm	6.958	-31.89 to 45.81	No	ns	>0.9999
Amine:5000 nm vs. Carboxyl:50 nm	-68.09	-106.9 to -29.24	Yes	****	<0.0001
Amine:5000 nm vs. Carboxyl:100 nm	13.81	-25.04 to 52.66	No	ns	0.9971
Amine:5000 nm vs. Carboxyl:200 nm	16.39	-22.46 to 55.24	No	ns	0.9827
Amine:5000 nm vs. Carboxyl:500 nm	3.873	-34.98 to 42.72	No	ns	>0.9999
Amine:5000 nm vs. Carboxyl:1000 nm	-11.36	-50.22 to 27.49	No	ns	0.9997
Amine:5000 nm vs. Carboxyl:5000 nm	3.537	-35.31 to 42.39	No	ns	>0.9999
Amine:5000 nm vs. NF:50 nm	9.818	-29.03 to 48.67	No	ns	>0.9999
Amine:5000 nm vs. NF:100 nm	13.34	-25.51 to 52.19	No	ns	0.9981
Amine:5000 nm vs. NF:200 nm	7.925	-30.93 to 46.78	No	ns	>0.9999
Amine:5000 nm vs. NF:500 nm	-0.2538	-39.10 to 38.60	No	ns	>0.9999
Amine:5000 nm vs. NF:1000 nm	6.962	-31.89 to 45.81	No	ns	>0.9999
Amine:5000 nm vs. NF:5000 nm	7.887	-30.96 to 46.74	No	ns	>0.9999
Carboxyl:50 nm vs. Carboxyl:100 nm	81.90	43.05 to 120.8	Yes	****	<0.0001
Carboxyl:50 nm vs. Carboxyl:200 nm	84.47	45.62 to 123.3	Yes	****	<0.0001
Carboxyl:50 nm vs. Carboxyl:500 nm	71.96	33.11 to 110.8	Yes	****	<0.0001
Carboxyl:50 nm vs. Carboxyl:1000 nm	56.72	17.87 to 95.57	Yes	***	0.0002
Carboxyl:50 nm vs. Carboxyl:5000 nm	71.63	32.78 to 110.5	Yes	****	<0.0001
Carboxyl:50 nm vs. NF:50 nm	77.91	39.06 to 116.8	Yes	****	<0.0001
Carboxyl:50 nm vs. NF:100 nm	81.43	42.58 to 120.3	Yes	****	<0.0001
Carboxyl:50 nm vs. NF:200 nm	76.01	37.16 to 114.9	Yes	****	<0.0001
Carboxyl:50 nm vs. NF:500 nm	67.84	28.98 to 106.7	Yes	****	<0.0001
Carboxyl:50 nm vs. NF:1000 nm	75.05	36.20 to 113.9	Yes	****	<0.0001
Carboxyl:50 nm vs. NF:5000 nm	75.98	37.13 to 114.8	Yes	****	<0.0001
Carboxyl:100 nm vs. Carboxyl:200 nm	2.572	-36.28 to 41.42	No	ns	>0.9999
Carboxyl:100 nm vs. Carboxyl:500 nm	-9.940	-48.79 to 28.91	No	ns	>0.9999
Carboxyl:100 nm vs. Carboxyl:1000 nm	-25.18	-64.03 to 13.67	No	ns	0.6292
Carboxyl:100 nm vs. Carboxyl:5000 nm	-10.28	-49.13 to 28.57	No	ns	>0.9999
Carboxyl:100 nm vs. NF:50 nm	-3.995	-42.85 to 34.86	No	ns	>0.9999
Carboxyl:100 nm vs. NF:100 nm	-0.4737	-39.32 to 38.38	No	ns	>0.9999
Carboxyl:100 nm vs. NF:200 nm	-5.889	-44.74 to 32.96	No	ns	>0.9999
Carboxyl:100 nm vs. NF:500 nm	-14.07	-52.92 to 24.78	No	ns	0.9965
Carboxyl:100 nm vs. NF:1000 nm	-6.852	-45.70 to 32.00	No	ns	>0.9999
Carboxyl:100 nm vs. NF:5000 nm	-5.927	-44.78 to 32.92	No	ns	>0.9999
Carboxyl:200 nm vs. Carboxyl:500 nm	-12.51	-51.36 to 26.34	No	ns	0.9991
Carboxyl:200 nm vs. Carboxyl:1000 nm	-27.75	-66.60 to 11.10	No	ns	0.4604
Carboxyl:200 nm vs. Carboxyl:5000 nm	-12.85	-51.70 to 26.00	No	ns	0.9988
Carboxyl:200 nm vs. NF:50 nm	-6.568	-45.42 to 32.28	No	ns	>0.9999
Carboxyl:200 nm vs. NF:100 nm	-3.046	-41.90 to 35.80	No	ns	>0.9999
Carboxyl:200 nm vs. NF:200 nm	-8.461	-47.31 to 30.39	No	ns	>0.9999

Carboxyl:200 nm vs. NF:500 nm	-16.64	-55.49 to 22.21	No	ns	0.9799
Carboxyl:200 nm vs. NF:1000 nm	-9.424	-48.27 to 29.43	No	ns	>0.9999
Carboxyl:200 nm vs. NF:5000 nm	-8.499	-47.35 to 30.35	No	ns	>0.9999
Carboxyl:500 nm vs. Carboxyl:1000 nm	-15.24	-54.09 to 23.61	No	ns	0.9917
Carboxyl:500 nm vs. Carboxyl:5000 nm	-0.3359	-39.19 to 38.51	No	ns	>0.9999
Carboxyl:500 nm vs. NF:50 nm	5.945	-32.91 to 44.80	No	ns	>0.9999
Carboxyl:500 nm vs. NF:100 nm	9.467	-29.38 to 48.32	No	ns	>0.9999
Carboxyl:500 nm vs. NF:200 nm	4.051	-34.80 to 42.90	No	ns	>0.9999
Carboxyl:500 nm vs. NF:500 nm	-4.127	-42.98 to 34.72	No	ns	>0.9999
Carboxyl:500 nm vs. NF:1000 nm	3.089	-35.76 to 41.94	No	ns	>0.9999
Carboxyl:500 nm vs. NF:5000 nm	4.014	-34.84 to 42.86	No	ns	>0.9999
Carboxyl:1000 nm vs. Carboxyl:5000 nm	14.90	-23.95 to 53.75	No	ns	0.9934
Carboxyl:1000 nm vs. NF:50 nm	21.18	-17.67 to 60.03	No	ns	0.8577
Carboxyl:1000 nm vs. NF:100 nm	24.70	-14.15 to 63.55	No	ns	0.6600
Carboxyl:1000 nm vs. NF:200 nm	19.29	-19.56 to 58.14	No	ns	0.9276
Carboxyl:1000 nm vs. NF:500 nm	11.11	-27.74 to 49.96	No	ns	0.9998
Carboxyl:1000 nm vs. NF:1000 nm	18.33	-20.52 to 57.18	No	ns	0.9522
Carboxyl:1000 nm vs. NF:5000 nm	19.25	-19.60 to 58.10	No	ns	0.9287
Carboxyl:5000 nm vs. NF:50 nm	6.281	-32.57 to 45.13	No	ns	>0.9999
Carboxyl:5000 nm vs. NF:100 nm	9.802	-29.05 to 48.65	No	ns	>0.9999
Carboxyl:5000 nm vs. NF:200 nm	4.387	-34.46 to 43.24	No	ns	>0.9999
Carboxyl:5000 nm vs. NF:500 nm	-3.791	-42.64 to 35.06	No	ns	>0.9999
Carboxyl:5000 nm vs. NF:1000 nm	3.425	-35.43 to 42.28	No	ns	>0.9999
Carboxyl:5000 nm vs. NF:5000 nm	4.350	-34.50 to 43.20	No	ns	>0.9999
NF:50 nm vs. NF:100 nm	3.521	-35.33 to 42.37	No	ns	>0.9999
NF:50 nm vs. NF:200 nm	-1.894	-40.74 to 36.96	No	ns	>0.9999
NF:50 nm vs. NF:500 nm	-10.07	-48.92 to 28.78	No	ns	>0.9999
NF:50 nm vs. NF:1000 nm	-2.856	-41.71 to 35.99	No	ns	>0.9999
NF:50 nm vs. NF:5000 nm	-1.931	-40.78 to 36.92	No	ns	>0.9999
NF:100 nm vs. NF:200 nm	-5.415	-44.27 to 33.44	No	ns	>0.9999
NF:100 nm vs. NF:500 nm	-13.59	-52.44 to 25.26	No	ns	0.9976
NF:100 nm vs. NF:1000 nm	-6.378	-45.23 to 32.47	No	ns	>0.9999
NF:100 nm vs. NF:5000 nm	-5.453	-44.30 to 33.40	No	ns	>0.9999
NF:200 nm vs. NF:500 nm	-8.179	-47.03 to 30.67	No	ns	>0.9999
NF:200 nm vs. NF:1000 nm	-0.9627	-39.81 to 37.89	No	ns	>0.9999
NF:200 nm vs. NF:5000 nm	-0.03770	-38.89 to 38.81	No	ns	>0.9999
NF:500 nm vs. NF:1000 nm	7.216	-31.63 to 46.07	No	ns	>0.9999
NF:500 nm vs. NF:5000 nm	8.141	-30.71 to 46.99	No	ns	>0.9999
NF:1000 nm vs. NF:5000 nm	0.9250	-37.93 to 39.78	No	ns	>0.9999
