# Supplementary material for: Uptake and toxicity of polystyrene micro/nanoplastics in gastric cells: Effects of particle size and surface functionalization
Source: PLoS One. 2021 Dec 31;16(12):e0260803. doi: 10.1371/journal.pone.0260803 (PMC8719689; doi:10.1371/journal.pone.0260803)
Supplement: S5 Table — (PDF) [file pone.0260803.s017.pdf]

Tukey's multiple comparisons test	Mean Diff.	95.0% CI of diff.	Below threshold?	Summary	Adjusted P Value
Amine:50 nm vs. Amine:100 nm	-0.003135	-0.9398 to 0.9335	No	ns	>0.9999
Amine:50 nm vs. Amine:200 nm	-0.3197	-1.256 to 0.6169	No	ns	0.9982
Amine:50 nm vs. Amine:500 nm	-0.7429	-1.680 to 0.1937	No	ns	0.2841
Amine:50 nm vs. Amine:1000 nm	-0.1975	-1.134 to 0.7392	No	ns	>0.9999
Amine:50 nm vs. Amine:5000 nm	-0.3824	-1.319 to 0.5542	No	ns	0.9874
Amine:50 nm vs. Carboxyl:50 nm	-0.08150	-1.018 to 0.8552	No	ns	>0.9999
Amine:50 nm vs. Carboxyl:100 nm	-0.2915	-1.228 to 0.6451	No	ns	0.9994
Amine:50 nm vs. Carboxyl:200 nm	-0.1034	-1.040 to 0.8332	No	ns	>0.9999
Amine:50 nm vs. Carboxyl:500 nm	-0.2884	-1.225 to 0.6483	No	ns	0.9995
Amine:50 nm vs. Carboxyl:1000 nm	-0.6113	-1.548 to 0.3254	No	ns	0.6177
Amine:50 nm vs. Carboxyl:5000 nm	0.05329	-0.8834 to 0.9900	No	ns	>0.9999
Amine:50 nm vs. NF:50 nm	-0.3354	-1.272 to 0.6013	No	ns	0.9969
Amine:50 nm vs. NF:100 nm	-0.06583	-1.003 to 0.8708	No	ns	>0.9999
Amine:50 nm vs. NF:200 nm	-0.4138	-1.350 to 0.5229	No	ns	0.9732
Amine:50 nm vs. NF:500 nm	-0.6238	-1.561 to 0.3129	No	ns	0.5833
Amine:50 nm vs. NF:1000 nm	-0.09718	-1.034 to 0.8395	No	ns	>0.9999
Amine:50 nm vs. NF:5000 nm	-0.2288	-1.166 to 0.7078	No	ns	>0.9999
Amine:100 nm vs. Amine:200 nm	-0.3166	-1.253 to 0.6201	No	ns	0.9984
Amine:100 nm vs. Amine:500 nm	-0.7398	-1.676 to 0.1969	No	ns	0.2907
Amine:100 nm vs. Amine:1000 nm	-0.1944	-1.131 to 0.7423	No	ns	>0.9999
Amine:100 nm vs. Amine:5000 nm	-0.3793	-1.316 to 0.5574	No	ns	0.9884
Amine:100 nm vs. Carboxyl:50 nm	-0.07837	-1.015 to 0.8583	No	ns	>0.9999
Amine:100 nm vs. Carboxyl:100 nm	-0.2884	-1.225 to 0.6483	No	ns	0.9995
Amine:100 nm vs. Carboxyl:200 nm	-0.1003	-1.037 to 0.8364	No	ns	>0.9999
Amine:100 nm vs. Carboxyl:500 nm	-0.2853	-1.222 to 0.6514	No	ns	0.9996
Amine:100 nm vs. Carboxyl:1000 nm	-0.6082	-1.545 to 0.3285	No	ns	0.6262
Amine:100 nm vs. Carboxyl:5000 nm	0.05643	-0.8803 to 0.9931	No	ns	>0.9999
Amine:100 nm vs. NF:50 nm	-0.3323	-1.269 to 0.6044	No	ns	0.9972
Amine:100 nm vs. NF:100 nm	-0.06270	-0.9994 to 0.8740	No	ns	>0.9999
Amine:100 nm vs. NF:200 nm	-0.4107	-1.347 to 0.5260	No	ns	0.9750
Amine:100 nm vs. NF:500 nm	-0.6207	-1.557 to 0.3160	No	ns	0.5919
Amine:100 nm vs. NF:1000 nm	-0.09404	-1.031 to 0.8426	No	ns	>0.9999
Amine:100 nm vs. NF:5000 nm	-0.2257	-1.162 to 0.7110	No	ns	>0.9999
Amine:200 nm vs. Amine:500 nm	-0.4232	-1.360 to 0.5135	No	ns	0.9671
Amine:200 nm vs. Amine:1000 nm	0.1223	-0.8144 to 1.059	No	ns	>0.9999
Amine:200 nm vs. Amine:5000 nm	-0.06270	-0.9994 to 0.8740	No	ns	>0.9999
Amine:200 nm vs. Carboxyl:50 nm	0.2382	-0.6984 to 1.175	No	ns	>0.9999
Amine:200 nm vs. Carboxyl:100 nm	0.02821	-0.9085 to 0.9649	No	ns	>0.9999
Amine:200 nm vs. Carboxyl:200 nm	0.2163	-0.7204 to 1.153	No	ns	>0.9999
Amine:200 nm vs. Carboxyl:500 nm	0.03135	-0.9053 to 0.9680	No	ns	>0.9999
Amine:200 nm vs. Carboxyl:1000 nm	-0.2915	-1.228 to 0.6451	No	ns	0.9994
Amine:200 nm vs. Carboxyl:5000 nm	0.3730	-0.5636 to 1.310	No	ns	0.9902
Amine:200 nm vs. NF:50 nm	-0.01567	-0.9524 to 0.9210	No	ns	>0.9999
Amine:200 nm vs. NF:100 nm	0.2539	-0.6828 to 1.191	No	ns	>0.9999
Amine:200 nm vs. NF:200 nm	-0.09404	-1.031 to 0.8426	No	ns	>0.9999
Amine:200 nm vs. NF:500 nm	-0.3041	-1.241 to 0.6326	No	ns	0.9990
Amine:200 nm vs. NF:1000 nm	0.2226	-0.7141 to 1.159	No	ns	>0.9999
Amine:200 nm vs. NF:5000 nm	0.09091	-0.8458 to 1.028	No	ns	>0.9999
Amine:500 nm vs. Amine:1000 nm	0.5455	-0.3912 to 1.482	No	ns	0.7857
Amine:500 nm vs. Amine:5000 nm	0.3605	-0.5762 to 1.297	No	ns	0.9932
Amine:500 nm vs. Carboxyl:50 nm	0.6614	-0.2752 to 1.598	No	ns	0.4807
Amine:500 nm vs. Carboxyl:100 nm	0.4514	-0.4853 to 1.388	No	ns	0.9430
Amine:500 nm vs. Carboxyl:200 nm	0.6395	-0.2972 to 1.576	No	ns	0.5403
Amine:500 nm vs. Carboxyl:500 nm	0.4545	-0.4821 to 1.391	No	ns	0.9397
Amine:500 nm vs. Carboxyl:1000 nm	0.1317	-0.8050 to 1.068	No	ns	>0.9999
Amine:500 nm vs. Carboxyl:5000 nm	0.7962	-0.1404 to 1.733	No	ns	0.1875

Amine:500 nm vs. NF:50 nm	0.4075	-0.5292 to 1.344	No	ns	0.9767
Amine:500 nm vs. NF:100 nm	0.6771	-0.2596 to 1.614	No	ns	0.4393
Amine:500 nm vs. NF:200 nm	0.3292	-0.6075 to 1.266	No	ns	0.9975
Amine:500 nm vs. NF:500 nm	0.1191	-0.8176 to 1.056	No	ns	>0.9999
Amine:500 nm vs. NF:1000 nm	0.6458	-0.2909 to 1.582	No	ns	0.5231
Amine:500 nm vs. NF:5000 nm	0.5141	-0.4226 to 1.451	No	ns	0.8514
Amine:1000 nm vs. Amine:5000 nm	-0.1850	-1.122 to 0.7517	No	ns	>0.9999
Amine:1000 nm vs. Carboxyl:50 nm	0.1160	-0.8207 to 1.053	No	ns	>0.9999
Amine:1000 nm vs. Carboxyl:100 nm	-0.09404	-1.031 to 0.8426	No	ns	>0.9999
Amine:1000 nm vs. Carboxyl:200 nm	0.09404	-0.8426 to 1.031	No	ns	>0.9999
Amine:1000 nm vs. Carboxyl:500 nm	-0.09091	-1.028 to 0.8458	No	ns	>0.9999
Amine:1000 nm vs. Carboxyl:1000 nm	-0.4138	-1.350 to 0.5229	No	ns	0.9732
Amine:1000 nm vs. Carboxyl:5000 nm	0.2508	-0.6859 to 1.187	No	ns	>0.9999
Amine:1000 nm vs. NF:50 nm	-0.1379	-1.075 to 0.7987	No	ns	>0.9999
Amine:1000 nm vs. NF:100 nm	0.1317	-0.8050 to 1.068	No	ns	>0.9999
Amine:1000 nm vs. NF:200 nm	-0.2163	-1.153 to 0.7204	No	ns	>0.9999
Amine:1000 nm vs. NF:500 nm	-0.4263	-1.363 to 0.5103	No	ns	0.9649
Amine:1000 nm vs. NF:1000 nm	0.1003	-0.8364 to 1.037	No	ns	>0.9999
Amine:1000 nm vs. NF:5000 nm	-0.03135	-0.9680 to 0.9053	No	ns	>0.9999
Amine:5000 nm vs. Carboxyl:50 nm	0.3009	-0.6357 to 1.238	No	ns	0.9991
Amine:5000 nm vs. Carboxyl:100 nm	0.09091	-0.8458 to 1.028	No	ns	>0.9999
Amine:5000 nm vs. Carboxyl:200 nm	0.2790	-0.6577 to 1.216	No	ns	0.9997
Amine:5000 nm vs. Carboxyl:500 nm	0.09404	-0.8426 to 1.031	No	ns	>0.9999
Amine:5000 nm vs. Carboxyl:1000 nm	-0.2288	-1.166 to 0.7078	No	ns	>0.9999
Amine:5000 nm vs. Carboxyl:5000 nm	0.4357	-0.5009 to 1.372	No	ns	0.9576
Amine:5000 nm vs. NF:50 nm	0.04702	-0.8897 to 0.9837	No	ns	>0.9999
Amine:5000 nm vs. NF:100 nm	0.3166	-0.6201 to 1.253	No	ns	0.9984
Amine:5000 nm vs. NF:200 nm	-0.03135	-0.9680 to 0.9053	No	ns	>0.9999
Amine:5000 nm vs. NF:500 nm	-0.2414	-1.178 to 0.6953	No	ns	>0.9999
Amine:5000 nm vs. NF:1000 nm	0.2853	-0.6514 to 1.222	No	ns	0.9996
Amine:5000 nm vs. NF:5000 nm	0.1536	-0.7831 to 1.090	No	ns	>0.9999
Carboxyl:50 nm vs. Carboxyl:100 nm	-0.2100	-1.147 to 0.7266	No	ns	>0.9999
Carboxyl:50 nm vs. Carboxyl:200 nm	-0.02194	-0.9586 to 0.9147	No	ns	>0.9999
Carboxyl:50 nm vs. Carboxyl:500 nm	-0.2069	-1.144 to 0.7298	No	ns	>0.9999
Carboxyl:50 nm vs. Carboxyl:1000 nm	-0.5298	-1.466 to 0.4069	No	ns	0.8200
Carboxyl:50 nm vs. Carboxyl:5000 nm	0.1348	-0.8019 to 1.071	No	ns	>0.9999
Carboxyl:50 nm vs. NF:50 nm	-0.2539	-1.191 to 0.6828	No	ns	>0.9999
Carboxyl:50 nm vs. NF:100 nm	0.01567	-0.9210 to 0.9524	No	ns	>0.9999
Carboxyl:50 nm vs. NF:200 nm	-0.3323	-1.269 to 0.6044	No	ns	0.9972
Carboxyl:50 nm vs. NF:500 nm	-0.5423	-1.479 to 0.3944	No	ns	0.7928
Carboxyl:50 nm vs. NF:1000 nm	-0.01567	-0.9524 to 0.9210	No	ns	>0.9999
Carboxyl:50 nm vs. NF:5000 nm	-0.1473	-1.084 to 0.7893	No	ns	>0.9999
Carboxyl:100 nm vs. Carboxyl:200 nm	0.1881	-0.7486 to 1.125	No	ns	>0.9999
Carboxyl:100 nm vs. Carboxyl:500 nm	0.003135	-0.9335 to 0.9398	No	ns	>0.9999
Carboxyl:100 nm vs. Carboxyl:1000 nm	-0.3197	-1.256 to 0.6169	No	ns	0.9982
Carboxyl:100 nm vs. Carboxyl:5000 nm	0.3448	-0.5919 to 1.282	No	ns	0.9958
Carboxyl:100 nm vs. NF:50 nm	-0.04389	-0.9806 to 0.8928	No	ns	>0.9999
Carboxyl:100 nm vs. NF:100 nm	0.2257	-0.7110 to 1.162	No	ns	>0.9999
Carboxyl:100 nm vs. NF:200 nm	-0.1223	-1.059 to 0.8144	No	ns	>0.9999
Carboxyl:100 nm vs. NF:500 nm	-0.3323	-1.269 to 0.6044	No	ns	0.9972
Carboxyl:100 nm vs. NF:1000 nm	0.1944	-0.7423 to 1.131	No	ns	>0.9999
Carboxyl:100 nm vs. NF:5000 nm	0.06270	-0.8740 to 0.9994	No	ns	>0.9999
Carboxyl:200 nm vs. Carboxyl:500 nm	-0.1850	-1.122 to 0.7517	No	ns	>0.9999
Carboxyl:200 nm vs. Carboxyl:1000 nm	-0.5078	-1.445 to 0.4288	No	ns	0.8630
Carboxyl:200 nm vs. Carboxyl:5000 nm	0.1567	-0.7799 to 1.093	No	ns	>0.9999
Carboxyl:200 nm vs. NF:50 nm	-0.2320	-1.169 to 0.7047	No	ns	>0.9999
Carboxyl:200 nm vs. NF:100 nm	0.03762	-0.8991 to 0.9743	No	ns	>0.9999
Carboxyl:200 nm vs. NF:200 nm	-0.3103	-1.247 to 0.6263	No	ns	0.9987

Carboxyl:200 nm vs. NF:500 nm	-0.5204	-1.457 to 0.4163	No	ns	0.8392
Carboxyl:200 nm vs. NF:1000 nm	0.006269	-0.9304 to 0.9429	No	ns	>0.9999
Carboxyl:200 nm vs. NF:5000 nm	-0.1254	-1.062 to 0.8113	No	ns	>0.9999
Carboxyl:500 nm vs. Carboxyl:1000 nm	-0.3229	-1.260 to 0.6138	No	ns	0.9980
Carboxyl:500 nm vs. Carboxyl:5000 nm	0.3417	-0.5950 to 1.278	No	ns	0.9962
Carboxyl:500 nm vs. NF:50 nm	-0.04702	-0.9837 to 0.8897	No	ns	>0.9999
Carboxyl:500 nm vs. NF:100 nm	0.2226	-0.7141 to 1.159	No	ns	>0.9999
Carboxyl:500 nm vs. NF:200 nm	-0.1254	-1.062 to 0.8113	No	ns	>0.9999
Carboxyl:500 nm vs. NF:500 nm	-0.3354	-1.272 to 0.6013	No	ns	0.9969
Carboxyl:500 nm vs. NF:1000 nm	0.1912	-0.7455 to 1.128	No	ns	>0.9999
Carboxyl:500 nm vs. NF:5000 nm	0.05956	-0.8771 to 0.9962	No	ns	>0.9999
Carboxyl:1000 nm vs. Carboxyl:5000 nm	0.6646	-0.2721 to 1.601	No	ns	0.4723
Carboxyl:1000 nm vs. NF:50 nm	0.2759	-0.6608 to 1.213	No	ns	0.9997
Carboxyl:1000 nm vs. NF:100 nm	0.5455	-0.3912 to 1.482	No	ns	0.7857
Carboxyl:1000 nm vs. NF:200 nm	0.1975	-0.7392 to 1.134	No	ns	>0.9999
Carboxyl:1000 nm vs. NF:500 nm	-0.01254	-0.9492 to 0.9241	No	ns	>0.9999
Carboxyl:1000 nm vs. NF:1000 nm	0.5141	-0.4226 to 1.451	No	ns	0.8514
Carboxyl:1000 nm vs. NF:5000 nm	0.3824	-0.5542 to 1.319	No	ns	0.9874
Carboxyl:5000 nm vs. NF:50 nm	-0.3887	-1.325 to 0.5480	No	ns	0.9852
Carboxyl:5000 nm vs. NF:100 nm	-0.1191	-1.056 to 0.8176	No	ns	>0.9999
Carboxyl:5000 nm vs. NF:200 nm	-0.4671	-1.404 to 0.4696	No	ns	0.9252
Carboxyl:5000 nm vs. NF:500 nm	-0.6771	-1.614 to 0.2596	No	ns	0.4393
Carboxyl:5000 nm vs. NF:1000 nm	-0.1505	-1.087 to 0.7862	No	ns	>0.9999
Carboxyl:5000 nm vs. NF:5000 nm	-0.2821	-1.219 to 0.6545	No	ns	0.9996
NF:50 nm vs. NF:100 nm	0.2696	-0.6671 to 1.206	No	ns	0.9998
NF:50 nm vs. NF:200 nm	-0.07837	-1.015 to 0.8583	No	ns	>0.9999
NF:50 nm vs. NF:500 nm	-0.2884	-1.225 to 0.6483	No	ns	0.9995
NF:50 nm vs. NF:1000 nm	0.2382	-0.6984 to 1.175	No	ns	>0.9999
NF:50 nm vs. NF:5000 nm	0.1066	-0.8301 to 1.043	No	ns	>0.9999
NF:100 nm vs. NF:200 nm	-0.3480	-1.285 to 0.5887	No	ns	0.9953
NF:100 nm vs. NF:500 nm	-0.5580	-1.495 to 0.3787	No	ns	0.7563
NF:100 nm vs. NF:1000 nm	-0.03135	-0.9680 to 0.9053	No	ns	>0.9999
NF:100 nm vs. NF:5000 nm	-0.1630	-1.100 to 0.7737	No	ns	>0.9999
NF:200 nm vs. NF:500 nm	-0.2100	-1.147 to 0.7266	No	ns	>0.9999
NF:200 nm vs. NF:1000 nm	0.3166	-0.6201 to 1.253	No	ns	0.9984
NF:200 nm vs. NF:5000 nm	0.1850	-0.7517 to 1.122	No	ns	>0.9999
NF:500 nm vs. NF:1000 nm	0.5266	-0.4100 to 1.463	No	ns	0.8266
NF:500 nm vs. NF:5000 nm	0.3950	-0.5417 to 1.332	No	ns	0.9827
NF:1000 nm vs. NF:5000 nm	-0.1317	-1.068 to 0.8050	No	ns	>0.9999
