# Supplementary material for: Uptake and toxicity of polystyrene micro/nanoplastics in gastric cells: Effects of particle size and surface functionalization
Source: PLoS One. 2021 Dec 31;16(12):e0260803. doi: 10.1371/journal.pone.0260803 (PMC8719689; doi:10.1371/journal.pone.0260803)
Supplement: S6 Table — (PDF) [file pone.0260803.s018.pdf]

Source	Nparm	DF	Sum of Squares	F Ratio	Prob > F
Bead surface functionalization	2	2	198.4448	29.9263	<.0001*
Bead size	5	5	1422.4877	85.8068	<.0001*
Bead surface functionalization * Bead size	10	10	732.3442	22.0881	<.0001*

Level	- Level	Difference	Std Err Dif	Lower CL	Upper CL	p-Value
Amine,50 nm	NF,1000 nm	25.64750	1.287548	20.9323	30.36269	<.0001*
Amine,50 nm	Amine,100 nm	23.97500	1.287548	19.2598	28.69019	<.0001*
Amine,50 nm	NF,5000 nm	23.88250	1.287548	19.1673	28.59769	<.0001*
Amine,50 nm	Carboxyl,100 nm	22.70250	1.287548	17.9873	27.41769	<.0001*
Amine,50 nm	Carboxyl,1000 nm	22.61750	1.287548	17.9023	27.33269	<.0001*
Amine,50 nm	NF,100 nm	21.62750	1.287548	16.9123	26.34269	<.0001*
Amine,50 nm	Amine,200 nm	20.72500	1.287548	16.0098	25.44019	<.0001*
Amine,50 nm	Carboxyl,5000 nm	20.22000	1.287548	15.5048	24.93519	<.0001*
Amine,50 nm	Amine,500 nm	18.82000	1.287548	14.1048	23.53519	<.0001*
Amine,50 nm	Amine,1000 nm	17.80000	1.287548	13.0848	22.51519	<.0001*
Amine,50 nm	Carboxyl,500 nm	16.97500	1.287548	12.2598	21.69019	<.0001*
Amine,50 nm	NF,200 nm	16.55000	1.287548	11.8348	21.26519	<.0001*
Amine,50 nm	NF,500 nm	16.37500	1.287548	11.6598	21.09019	<.0001*
Amine,50 nm	Carboxyl,200 nm	15.92500	1.287548	11.2098	20.64019	<.0001*
Amine,50 nm	NF,50 nm	15.17500	1.287548	10.4598	19.89019	<.0001*
Amine,50 nm	Amine,5000 nm	13.90000	1.287548	9.1848	18.61519	<.0001*
Carboxyl,50 nm	NF,1000 nm	13.22250	1.287548	8.5073	17.93769	<.0001*
Amine,50 nm	Carboxyl,50 nm	12.42500	1.287548	7.7098	17.14019	<.0001*
Amine,5000 nm	NF,1000 nm	11.74750	1.287548	7.0323	16.46269	<.0001*
Carboxyl,50 nm	Amine,100 nm	11.55000	1.287548	6.8348	16.26519	<.0001*
Carboxyl,50 nm	NF,5000 nm	11.45750	1.287548	6.7423	16.17269	<.0001*
NF,50 nm	NF,1000 nm	10.47250	1.287548	5.7573	15.18769	<.0001*
Carboxyl,50 nm	Carboxyl,100 nm	10.27750	1.287548	5.5623	14.99269	<.0001*
Carboxyl,50 nm	Carboxyl,1000 nm	10.19250	1.287548	5.4773	14.90769	<.0001*
Amine,5000 nm	Amine,100 nm	10.07500	1.287548	5.3598	14.79019	<.0001*
Amine,5000 nm	NF,5000 nm	9.98250	1.287548	5.2673	14.69769	<.0001*
Carboxyl,200 nm	NF,1000 nm	9.72250	1.287548	5.0073	14.43769	<.0001*
NF,500 nm	NF,1000 nm	9.27250	1.287548	4.5573	13.98769	<.0001*
Carboxyl,50 nm	NF,100 nm	9.20250	1.287548	4.4873	13.91769	<.0001*
NF,200 nm	NF,1000 nm	9.09750	1.287548	4.3823	13.81269	<.0001*
Amine,5000 nm	Carboxyl,100 nm	8.80250	1.287548	4.0873	13.51769	<.0001*
NF,50 nm	Amine,100 nm	8.80000	1.287548	4.0848	13.51519	<.0001*
Amine,5000 nm	Carboxyl,1000 nm	8.71750	1.287548	4.0023	13.43269	<.0001*
NF,50 nm	NF,5000 nm	8.70750	1.287548	3.9923	13.42269	<.0001*
Carboxyl,500 nm	NF,1000 nm	8.67250	1.287548	3.9573	13.38769	<.0001*
Carboxyl,50 nm	Amine,200 nm	8.30000	1.287548	3.5848	13.01519	<.0001*
Carboxyl,200 nm	Amine,100 nm	8.05000	1.287548	3.3348	12.76519	<.0001*
Carboxyl,200 nm	NF,5000 nm	7.95750	1.287548	3.2423	12.67269	<.0001*
Amine,1000 nm	NF,1000 nm	7.84750	1.287548	3.1323	12.56269	<.0001*
Carboxyl,50 nm	Carboxyl,5000 nm	7.79500	1.287548	3.0798	12.51019	<.0001*
Amine,5000 nm	NF,100 nm	7.72750	1.287548	3.0123	12.44269	<.0001*
NF,500 nm	Amine,100 nm	7.60000	1.287548	2.8848	12.31519	<.0001*
NF,50 nm	Carboxyl,100 nm	7.52750	1.287548	2.8123	12.24269	<.0001*
NF,500 nm	NF,5000 nm	7.50750	1.287548	2.7923	12.22269	<.0001*
NF,50 nm	Carboxyl,1000 nm	7.44250	1.287548	2.7273	12.15769	<.0001*
NF,200 nm	Amine,100 nm	7.42500	1.287548	2.7098	12.14019	<.0001*
NF,200 nm	NF,5000 nm	7.33250	1.287548	2.6173	12.04769	<.0001*

Level	- Level	Difference	Std Err Dif	Lower CL	Upper CL	p-Value
Carboxyl,500 nm	Amine,100 nm	7.00000	1.287548	2.2848	11.71519	0.0002*
Carboxyl,500 nm	NF,5000 nm	6.90750	1.287548	2.1923	11.62269	0.0002*
Amine,500 nm	NF,1000 nm	6.82750	1.287548	2.1123	11.54269	0.0003*
Amine,5000 nm	Amine,200 nm	6.82500	1.287548	2.1098	11.54019	0.0003*
Carboxyl,200 nm	Carboxyl,100 nm	6.77750	1.287548	2.0623	11.49269	0.0003*
Carboxyl,200 nm	Carboxyl,1000 nm	6.69250	1.287548	1.9773	11.40769	0.0004*
NF,50 nm	NF,100 nm	6.45250	1.287548	1.7373	11.16769	0.0008*
Carboxyl,50 nm	Amine,500 nm	6.39500	1.287548	1.6798	11.11019	0.0009*
NF,500 nm	Carboxyl,100 nm	6.32750	1.287548	1.6123	11.04269	0.0011*
Amine,5000 nm	Carboxyl,5000 nm	6.32000	1.287548	1.6048	11.03519	0.0011*
NF,500 nm	Carboxyl,1000 nm	6.24250	1.287548	1.5273	10.95769	0.0013*
Amine,1000 nm	Amine,100 nm	6.17500	1.287548	1.4598	10.89019	0.0016*
NF,200 nm	Carboxyl,100 nm	6.15250	1.287548	1.4373	10.86769	0.0017*
Amine,1000 nm	NF,5000 nm	6.08250	1.287548	1.3673	10.79769	0.0020*
NF,200 nm	Carboxyl,1000 nm	6.06750	1.287548	1.3523	10.78269	0.0021*
Carboxyl,500 nm	Carboxyl,100 nm	5.72750	1.287548	1.0123	10.44269	0.0050*
Carboxyl,200 nm	NF,100 nm	5.70250	1.287548	0.9873	10.41769	0.0053*
Carboxyl,500 nm	Carboxyl,1000 nm	5.64250	1.287548	0.9273	10.35769	0.0061*
NF,50 nm	Amine,200 nm	5.55000	1.287548	0.8348	10.26519	0.0076*
Carboxyl,5000 nm	NF,1000 nm	5.42750	1.287548	0.7123	10.14269	0.0102*
Carboxyl,50 nm	Amine,1000 nm	5.37500	1.287548	0.6598	10.09019	0.0116*
NF,500 nm	NF,100 nm	5.25250	1.287548	0.5373	9.96769	0.0154*
Amine,500 nm	Amine,100 nm	5.15500	1.287548	0.4398	9.87019	0.0193*
NF,200 nm	NF,100 nm	5.07750	1.287548	0.3623	9.79269	0.0229*
Amine,500 nm	NF,5000 nm	5.06250	1.287548	0.3473	9.77769	0.0237*
NF,50 nm	Carboxyl,5000 nm	5.04500	1.287548	0.3298	9.76019	0.0246*
Amine,200 nm	NF,1000 nm	4.92250	1.287548	0.2073	9.63769	0.0322*
Amine,5000 nm	Amine,500 nm	4.92000	1.287548	0.2048	9.63519	0.0324*
Amine,1000 nm	Carboxyl,100 nm	4.90250	1.287548	0.1873	9.61769	0.0337*
Amine,1000 nm	Carboxyl,1000 nm	4.81750	1.287548	0.1023	9.53269	0.0404*
Carboxyl,200 nm	Amine,200 nm	4.80000	1.287548	0.0848	9.51519	0.0419*
Carboxyl,500 nm	NF,100 nm	4.65250	1.287548	-0.0627	9.36769	0.0569
Carboxyl,50 nm	Carboxyl,500 nm	4.55000	1.287548	-0.1652	9.26519	0.0699
NF,500 nm	Amine,200 nm	4.35000	1.287548	-0.3652	9.06519	0.1027
Carboxyl,200 nm	Carboxyl,5000 nm	4.29500	1.287548	-0.4202	9.01019	0.1137
NF,200 nm	Amine,200 nm	4.17500	1.287548	-0.5402	8.89019	0.1411
Carboxyl,50 nm	NF,200 nm	4.12500	1.287548	-0.5902	8.84019	0.1539
NF,100 nm	NF,1000 nm	4.02000	1.287548	-0.6952	8.73519	0.1838
Carboxyl,50 nm	NF,500 nm	3.95000	1.287548	-0.7652	8.66519	0.2061
Amine,5000 nm	Amine,1000 nm	3.90000	1.287548	-0.8152	8.61519	0.2231
Amine,500 nm	Carboxyl,100 nm	3.88250	1.287548	-0.8327	8.59769	0.2293
NF,500 nm	Carboxyl,5000 nm	3.84500	1.287548	-0.8702	8.56019	0.2430
Amine,1000 nm	NF,100 nm	3.82750	1.287548	-0.8877	8.54269	0.2495
Amine,500 nm	Carboxyl,1000 nm	3.79750	1.287548	-0.9177	8.51269	0.2611
Carboxyl,5000 nm	Amine,100 nm	3.75500	1.287548	-0.9602	8.47019	0.2780
Carboxyl,500 nm	Amine,200 nm	3.75000	1.287548	-0.9652	8.46519	0.2800
NF,200 nm	Carboxyl,5000 nm	3.67000	1.287548	-1.0452	8.38519	0.3138
Carboxyl,5000 nm	NF,5000 nm	3.66250	1.287548	-1.0527	8.37769	0.3171
NF,50 nm	Amine,500 nm	3.64500	1.287548	-1.0702	8.36019	0.3249
Carboxyl,50 nm	Carboxyl,200 nm	3.50000	1.287548	-1.2152	8.21519	0.3931
Amine,200 nm	Amine,100 nm	3.25000	1.287548	-1.4652	7.96519	0.5235
Carboxyl,500 nm	Carboxyl,5000 nm	3.24500	1.287548	-1.4702	7.96019	0.5262
Amine,200 nm	NF,5000 nm	3.15750	1.287548	-1.5577	7.87269	0.5739

Level	- Level	Difference	Std Err Dif	Lower CL	Upper CL	p-Value
Amine,5000 nm	Carboxyl,500 nm	3.07500	1.287548	-1.6402	7.79019	0.6188
Carboxyl,1000 nm	NF,1000 nm	3.03000	1.287548	-1.6852	7.74519	0.6431
Carboxyl,100 nm	NF,1000 nm	2.94500	1.287548	-1.7702	7.66019	0.6881
Amine,1000 nm	Amine,200 nm	2.92500	1.287548	-1.7902	7.64019	0.6985
Carboxyl,200 nm	Amine,500 nm	2.89500	1.287548	-1.8202	7.61019	0.7138
Amine,500 nm	NF,100 nm	2.80750	1.287548	-1.9077	7.52269	0.7570
Carboxyl,50 nm	NF,50 nm	2.75000	1.287548	-1.9652	7.46519	0.7838
Amine,5000 nm	NF,200 nm	2.65000	1.287548	-2.0652	7.36519	0.8270
NF,50 nm	Amine,1000 nm	2.62500	1.287548	-2.0902	7.34019	0.8371
Carboxyl,5000 nm	Carboxyl,100 nm	2.48250	1.287548	-2.2327	7.19769	0.8881
Amine,5000 nm	NF,500 nm	2.47500	1.287548	-2.2402	7.19019	0.8904
NF,500 nm	Amine,500 nm	2.44500	1.287548	-2.2702	7.16019	0.8996
Amine,1000 nm	Carboxyl,5000 nm	2.42000	1.287548	-2.2952	7.13519	0.9069
Carboxyl,5000 nm	Carboxyl,1000 nm	2.39750	1.287548	-2.3177	7.11269	0.9132
NF,100 nm	Amine,100 nm	2.34750	1.287548	-2.3677	7.06269	0.9261
NF,200 nm	Amine,500 nm	2.27000	1.287548	-2.4452	6.98519	0.9434
NF,100 nm	NF,5000 nm	2.25500	1.287548	-2.4602	6.97019	0.9464
Amine,5000 nm	Carboxyl,200 nm	2.02500	1.287548	-2.6902	6.74019	0.9794
Amine,200 nm	Carboxyl,100 nm	1.97750	1.287548	-2.7377	6.69269	0.9836
Amine,500 nm	Amine,200 nm	1.90500	1.287548	-2.8102	6.62019	0.9887
Amine,200 nm	Carboxyl,1000 nm	1.89250	1.287548	-2.8227	6.60769	0.9894
Carboxyl,200 nm	Amine,1000 nm	1.87500	1.287548	-2.8402	6.59019	0.9904
Carboxyl,500 nm	Amine,500 nm	1.84500	1.287548	-2.8702	6.56019	0.9919
NF,50 nm	Carboxyl,500 nm	1.80000	1.287548	-2.9152	6.51519	0.9937
NF,5000 nm	NF,1000 nm	1.76500	1.287548	-2.9502	6.48019	0.9949
Amine,100 nm	NF,1000 nm	1.67250	1.287548	-3.0427	6.38769	0.9972
Carboxyl,50 nm	Amine,5000 nm	1.47500	1.287548	-3.2402	6.19019	0.9994
NF,500 nm	Amine,1000 nm	1.42500	1.287548	-3.2902	6.14019	0.9996
Carboxyl,5000 nm	NF,100 nm	1.40750	1.287548	-3.3077	6.12269	0.9997
Amine,500 nm	Carboxyl,5000 nm	1.40000	1.287548	-3.3152	6.11519	0.9997
NF,50 nm	NF,200 nm	1.37500	1.287548	-3.3402	6.09019	0.9997
Carboxyl,1000 nm	Amine,100 nm	1.35750	1.287548	-3.3577	6.07269	0.9998
Amine,5000 nm	NF,50 nm	1.27500	1.287548	-3.4402	5.99019	0.9999
Carboxyl,100 nm	Amine,100 nm	1.27250	1.287548	-3.4427	5.98769	0.9999
Carboxyl,1000 nm	NF,5000 nm	1.26500	1.287548	-3.4502	5.98019	0.9999
NF,200 nm	Amine,1000 nm	1.25000	1.287548	-3.4652	5.96519	0.9999
NF,50 nm	NF,500 nm	1.20000	1.287548	-3.5152	5.91519	1.0000
Carboxyl,100 nm	NF,5000 nm	1.18000	1.287548	-3.5352	5.89519	1.0000
NF,100 nm	Carboxyl,100 nm	1.07500	1.287548	-3.6402	5.79019	1.0000
Carboxyl,200 nm	Carboxyl,500 nm	1.05000	1.287548	-3.6652	5.76519	1.0000
Amine,1000 nm	Amine,500 nm	1.02000	1.287548	-3.6952	5.73519	1.0000
NF,100 nm	Carboxyl,1000 nm	0.99000	1.287548	-3.7252	5.70519	1.0000
Amine,200 nm	NF,100 nm	0.90250	1.287548	-3.8127	5.61769	1.0000
Carboxyl,500 nm	Amine,1000 nm	0.82500	1.287548	-3.8902	5.54019	1.0000
NF,50 nm	Carboxyl,200 nm	0.75000	1.287548	-3.9652	5.46519	1.0000
Carboxyl,200 nm	NF,200 nm	0.62500	1.287548	-4.0902	5.34019	1.0000
NF,500 nm	Carboxyl,500 nm	0.60000	1.287548	-4.1152	5.31519	1.0000
Carboxyl,5000 nm	Amine,200 nm	0.50500	1.287548	-4.2102	5.22019	1.0000
Carboxyl,200 nm	NF,500 nm	0.45000	1.287548	-4.2652	5.16519	1.0000
NF,200 nm	Carboxyl,500 nm	0.42500	1.287548	-4.2902	5.14019	1.0000
NF,500 nm	NF,200 nm	0.17500	1.287548	-4.5402	4.89019	1.0000
NF,5000 nm	Amine,100 nm	0.09250	1.287548	-4.6227	4.80769	1.0000
Carboxyl,1000 nm	Carboxyl,100 nm	0.08500	1.287548	-4.6302	4.80019	1.0000
