# Supplementary material for: Uptake and toxicity of polystyrene micro/nanoplastics in gastric cells: Effects of particle size and surface functionalization
Source: PLoS One. 2021 Dec 31;16(12):e0260803. doi: 10.1371/journal.pone.0260803 (PMC8719689; doi:10.1371/journal.pone.0260803)
Supplement: S7 Table — (PDF) [file pone.0260803.s019.pdf]

Tukey's multiple comparisons test	Mean Diff.	95.00% CI of diff.	Below threshold?	Summary	Adjusted P Value
Amine:50 nm vs. Amine:100 nm	5.580	2.401 to 8.759	Yes	****	<0.0001
Amine:50 nm vs. Amine:200 nm	1.155	-2.024 to 4.334	No	ns	0.9963
Amine:50 nm vs. Amine:500 nm	2.055	-1.124 to 5.234	No	ns	0.6332
Amine:50 nm vs. Amine:1000 nm	10.94	7.764 to 14.12	Yes	****	<0.0001
Amine:50 nm vs. Amine:5000 nm	4.580	1.401 to 7.759	Yes	***	0.0003
Amine:50 nm vs. Carboxyl:50 nm	-32.45	-35.62 to -29.27	Yes	****	<0.0001
Amine:50 nm vs. Carboxyl:100 nm	1.480	-1.699 to 4.659	No	ns	0.9572
Amine:50 nm vs. Carboxyl:200 nm	6.095	2.916 to 9.274	Yes	****	<0.0001
Amine:50 nm vs. Carboxyl:500 nm	7.953	4.774 to 11.13	Yes	****	<0.0001
Amine:50 nm vs. Carboxyl:1000 nm	12.08	8.901 to 15.26	Yes	****	<0.0001
Amine:50 nm vs. Carboxyl:5000 nm	6.605	3.426 to 9.784	Yes	****	<0.0001
Amine:50 nm vs. NF:50 nm	-19.02	-22.20 to -15.84	Yes	****	<0.0001
Amine:50 nm vs. NF:100 nm	-1.520	-4.699 to 1.659	No	ns	0.9465
Amine:50 nm vs. NF:200 nm	3.805	0.6264 to 6.984	Yes	**	0.0061
Amine:50 nm vs. NF:500 nm	7.118	3.939 to 10.30	Yes	****	<0.0001
Amine:50 nm vs. NF:1000 nm	11.10	7.916 to 14.27	Yes	****	<0.0001
Amine:50 nm vs. NF:5000 nm	9.775	6.596 to 12.95	Yes	****	<0.0001
Amine:100 nm vs. Amine:200 nm	-4.425	-7.604 to -1.246	Yes	***	0.0006
Amine:100 nm vs. Amine:500 nm	-3.525	-6.704 to -0.3464	Yes	*	0.0163
Amine:100 nm vs. Amine:1000 nm	5.363	2.184 to 8.541	Yes	****	<0.0001
Amine:100 nm vs. Amine:5000 nm	-1.000	-4.179 to 2.179	No	ns	0.9993
Amine:100 nm vs. Carboxyl:50 nm	-38.03	-41.20 to -34.85	Yes	****	<0.0001
Amine:100 nm vs. Carboxyl:100 nm	-4.100	-7.279 to -0.9214	Yes	**	0.0020
Amine:100 nm vs. Carboxyl:200 nm	0.5150	-2.664 to 3.694	No	ns	>0.9999
Amine:100 nm vs. Carboxyl:500 nm	2.373	-0.8061 to 5.551	No	ns	0.3836
Amine:100 nm vs. Carboxyl:1000 nm	6.500	3.321 to 9.679	Yes	****	<0.0001
Amine:100 nm vs. Carboxyl:5000 nm	1.025	-2.154 to 4.204	No	ns	0.9991
Amine:100 nm vs. NF:50 nm	-24.60	-27.78 to -21.42	Yes	****	<0.0001
Amine:100 nm vs. NF:100 nm	-7.100	-10.28 to -3.921	Yes	****	<0.0001
Amine:100 nm vs. NF:200 nm	-1.775	-4.954 to 1.404	No	ns	0.8339
Amine:100 nm vs. NF:500 nm	1.538	-1.641 to 4.716	No	ns	0.9412
Amine:100 nm vs. NF:1000 nm	5.515	2.336 to 8.694	Yes	****	<0.0001
Amine:100 nm vs. NF:5000 nm	4.195	1.016 to 7.374	Yes	**	0.0014
Amine:200 nm vs. Amine:500 nm	0.9000	-2.279 to 4.079	No	ns	0.9998
Amine:200 nm vs. Amine:1000 nm	9.788	6.609 to 12.97	Yes	****	<0.0001
Amine:200 nm vs. Amine:5000 nm	3.425	0.2464 to 6.604	Yes	*	0.0228
Amine:200 nm vs. Carboxyl:50 nm	-33.60	-36.78 to -30.42	Yes	****	<0.0001
Amine:200 nm vs. Carboxyl:100 nm	0.3250	-2.854 to 3.504	No	ns	>0.9999
Amine:200 nm vs. Carboxyl:200 nm	4.940	1.761 to 8.119	Yes	****	<0.0001
Amine:200 nm vs. Carboxyl:500 nm	6.798	3.619 to 9.976	Yes	****	<0.0001
Amine:200 nm vs. Carboxyl:1000 nm	10.93	7.746 to 14.10	Yes	****	<0.0001
Amine:200 nm vs. Carboxyl:5000 nm	5.450	2.271 to 8.629	Yes	****	<0.0001
Amine:200 nm vs. NF:50 nm	-20.18	-23.35 to -17.00	Yes	****	<0.0001
Amine:200 nm vs. NF:100 nm	-2.675	-5.854 to 0.5036	No	ns	0.2001
Amine:200 nm vs. NF:200 nm	2.650	-0.5286 to 5.829	No	ns	0.2124
Amine:200 nm vs. NF:500 nm	5.963	2.784 to 9.141	Yes	****	<0.0001
Amine:200 nm vs. NF:1000 nm	9.940	6.761 to 13.12	Yes	****	<0.0001
Amine:200 nm vs. NF:5000 nm	8.620	5.441 to 11.80	Yes	****	<0.0001
Amine:500 nm vs. Amine:1000 nm	8.888	5.709 to 12.07	Yes	****	<0.0001
Amine:500 nm vs. Amine:5000 nm	2.525	-0.6536 to 5.704	No	ns	0.2818
Amine:500 nm vs. Carboxyl:50 nm	-34.50	-37.68 to -31.32	Yes	****	<0.0001
Amine:500 nm vs. Carboxyl:100 nm	-0.5750	-3.754 to 2.604	No	ns	>0.9999
Amine:500 nm vs. Carboxyl:200 nm	4.040	0.8614 to 7.219	Yes	**	0.0026
Amine:500 nm vs. Carboxyl:500 nm	5.898	2.719 to 9.076	Yes	****	<0.0001
Amine:500 nm vs. Carboxyl:1000 nm	10.03	6.846 to 13.20	Yes	****	<0.0001
Amine:500 nm vs. Carboxyl:5000 nm	4.550	1.371 to 7.729	Yes	***	0.0004

Amine:500 nm vs. NF:50 nm	-21.08	-24.25 to -17.90	Yes	****	<0.0001
Amine:500 nm vs. NF:100 nm	-3.575	-6.754 to -0.3964	Yes	*	0.0137
Amine:500 nm vs. NF:200 nm	1.750	-1.429 to 4.929	No	ns	0.8483
Amine:500 nm vs. NF:500 nm	5.063	1.884 to 8.241	Yes	****	<0.0001
Amine:500 nm vs. NF:1000 nm	9.040	5.861 to 12.22	Yes	****	<0.0001
Amine:500 nm vs. NF:5000 nm	7.720	4.541 to 10.90	Yes	****	<0.0001
Amine:1000 nm vs. Amine:5000 nm	-6.363	-9.541 to -3.184	Yes	****	<0.0001
Amine:1000 nm vs. Carboxyl:50 nm	-43.39	-46.57 to -40.21	Yes	****	<0.0001
Amine:1000 nm vs. Carboxyl:100 nm	-9.463	-12.64 to -6.284	Yes	****	<0.0001
Amine:1000 nm vs. Carboxyl:200 nm	-4.848	-8.026 to -1.669	Yes	***	0.0001
Amine:1000 nm vs. Carboxyl:500 nm	-2.990	-6.169 to 0.1886	No	ns	0.0874
Amine:1000 nm vs. Carboxyl:1000 nm	1.138	-2.041 to 4.316	No	ns	0.9969
Amine:1000 nm vs. Carboxyl:5000 nm	-4.338	-7.516 to -1.159	Yes	***	0.0008
Amine:1000 nm vs. NF:50 nm	-29.96	-33.14 to -26.78	Yes	****	<0.0001
Amine:1000 nm vs. NF:100 nm	-12.46	-15.64 to -9.284	Yes	****	<0.0001
Amine:1000 nm vs. NF:200 nm	-7.138	-10.32 to -3.959	Yes	****	<0.0001
Amine:1000 nm vs. NF:500 nm	-3.825	-7.004 to -0.6464	Yes	**	0.0057
Amine:1000 nm vs. NF:1000 nm	0.1525	-3.026 to 3.331	No	ns	>0.9999
Amine:1000 nm vs. NF:5000 nm	-1.168	-4.346 to 2.011	No	ns	0.9959
Amine:5000 nm vs. Carboxyl:50 nm	-37.03	-40.20 to -33.85	Yes	****	<0.0001
Amine:5000 nm vs. Carboxyl:100 nm	-3.100	-6.279 to 0.07861	No	ns	0.0634
Amine:5000 nm vs. Carboxyl:200 nm	1.515	-1.664 to 4.694	No	ns	0.9479
Amine:5000 nm vs. Carboxyl:500 nm	3.373	0.1939 to 6.551	Yes	*	0.0270
Amine:5000 nm vs. Carboxyl:1000 nm	7.500	4.321 to 10.68	Yes	****	<0.0001
Amine:5000 nm vs. Carboxyl:5000 nm	2.025	-1.154 to 5.204	No	ns	0.6571
Amine:5000 nm vs. NF:50 nm	-23.60	-26.78 to -20.42	Yes	****	<0.0001
Amine:5000 nm vs. NF:100 nm	-6.100	-9.279 to -2.921	Yes	****	<0.0001
Amine:5000 nm vs. NF:200 nm	-0.7750	-3.954 to 2.404	No	ns	>0.9999
Amine:5000 nm vs. NF:500 nm	2.538	-0.6411 to 5.716	No	ns	0.2743
Amine:5000 nm vs. NF:1000 nm	6.515	3.336 to 9.694	Yes	****	<0.0001
Amine:5000 nm vs. NF:5000 nm	5.195	2.016 to 8.374	Yes	****	<0.0001
Carboxyl:50 nm vs. Carboxyl:100 nm	33.93	30.75 to 37.10	Yes	****	<0.0001
Carboxyl:50 nm vs. Carboxyl:200 nm	38.54	35.36 to 41.72	Yes	****	<0.0001
Carboxyl:50 nm vs. Carboxyl:500 nm	40.40	37.22 to 43.58	Yes	****	<0.0001
Carboxyl:50 nm vs. Carboxyl:1000 nm	44.53	41.35 to 47.70	Yes	****	<0.0001
Carboxyl:50 nm vs. Carboxyl:5000 nm	39.05	35.87 to 42.23	Yes	****	<0.0001
Carboxyl:50 nm vs. NF:50 nm	13.43	10.25 to 16.60	Yes	****	<0.0001
Carboxyl:50 nm vs. NF:100 nm	30.93	27.75 to 34.10	Yes	****	<0.0001
Carboxyl:50 nm vs. NF:200 nm	36.25	33.07 to 39.43	Yes	****	<0.0001
Carboxyl:50 nm vs. NF:500 nm	39.56	36.38 to 42.74	Yes	****	<0.0001
Carboxyl:50 nm vs. NF:1000 nm	43.54	40.36 to 46.72	Yes	****	<0.0001
Carboxyl:50 nm vs. NF:5000 nm	42.22	39.04 to 45.40	Yes	****	<0.0001
Carboxyl:100 nm vs. Carboxyl:200 nm	4.615	1.436 to 7.794	Yes	***	0.0003
Carboxyl:100 nm vs. Carboxyl:500 nm	6.473	3.294 to 9.651	Yes	****	<0.0001
Carboxyl:100 nm vs. Carboxyl:1000 nm	10.60	7.421 to 13.78	Yes	****	<0.0001
Carboxyl:100 nm vs. Carboxyl:5000 nm	5.125	1.946 to 8.304	Yes	****	<0.0001
Carboxyl:100 nm vs. NF:50 nm	-20.50	-23.68 to -17.32	Yes	****	<0.0001
Carboxyl:100 nm vs. NF:100 nm	-3.000	-6.179 to 0.1786	No	ns	0.0849
Carboxyl:100 nm vs. NF:200 nm	2.325	-0.8536 to 5.504	No	ns	0.4187
Carboxyl:100 nm vs. NF:500 nm	5.638	2.459 to 8.816	Yes	****	<0.0001
Carboxyl:100 nm vs. NF:1000 nm	9.615	6.436 to 12.79	Yes	****	<0.0001
Carboxyl:100 nm vs. NF:5000 nm	8.295	5.116 to 11.47	Yes	****	<0.0001
Carboxyl:200 nm vs. Carboxyl:500 nm	1.858	-1.321 to 5.036	No	ns	0.7813
Carboxyl:200 nm vs. Carboxyl:1000 nm	5.985	2.806 to 9.164	Yes	****	<0.0001
Carboxyl:200 nm vs. Carboxyl:5000 nm	0.5100	-2.669 to 3.689	No	ns	>0.9999
Carboxyl:200 nm vs. NF:50 nm	-25.12	-28.29 to -21.94	Yes	****	<0.0001
Carboxyl:200 nm vs. NF:100 nm	-7.615	-10.79 to -4.436	Yes	****	<0.0001
Carboxyl:200 nm vs. NF:200 nm	-2.290	-5.469 to 0.8886	No	ns	0.4453

Carboxyl:200 nm vs. NF:500 nm	1.023	-2.156 to 4.201	No	ns	0.9991
Carboxyl:200 nm vs. NF:1000 nm	5.000	1.821 to 8.179	Yes	****	<0.0001
Carboxyl:200 nm vs. NF:5000 nm	3.680	0.5014 to 6.859	Yes	**	0.0095
Carboxyl:500 nm vs. Carboxyl:1000 nm	4.128	0.9489 to 7.306	Yes	**	0.0018
Carboxyl:500 nm vs. Carboxyl:5000 nm	-1.348	-4.526 to 1.831	No	ns	0.9818
Carboxyl:500 nm vs. NF:50 nm	-26.97	-30.15 to -23.79	Yes	****	<0.0001
Carboxyl:500 nm vs. NF:100 nm	-9.473	-12.65 to -6.294	Yes	****	<0.0001
Carboxyl:500 nm vs. NF:200 nm	-4.148	-7.326 to -0.9689	Yes	**	0.0017
Carboxyl:500 nm vs. NF:500 nm	-0.8350	-4.014 to 2.344	No	ns	>0.9999
Carboxyl:500 nm vs. NF:1000 nm	3.143	-0.03611 to 6.321	No	ns	0.0558
Carboxyl:500 nm vs. NF:5000 nm	1.823	-1.356 to 5.001	No	ns	0.8045
Carboxyl:1000 nm vs. Carboxyl:5000 nm	-5.475	-8.654 to -2.296	Yes	****	<0.0001
Carboxyl:1000 nm vs. NF:50 nm	-31.10	-34.28 to -27.92	Yes	****	<0.0001
Carboxyl:1000 nm vs. NF:100 nm	-13.60	-16.78 to -10.42	Yes	****	<0.0001
Carboxyl:1000 nm vs. NF:200 nm	-8.275	-11.45 to -5.096	Yes	****	<0.0001
Carboxyl:1000 nm vs. NF:500 nm	-4.963	-8.141 to -1.784	Yes	****	<0.0001
Carboxyl:1000 nm vs. NF:1000 nm	-0.9850	-4.164 to 2.194	No	ns	0.9994
Carboxyl:1000 nm vs. NF:5000 nm	-2.305	-5.484 to 0.8736	No	ns	0.4338
Carboxyl:5000 nm vs. NF:50 nm	-25.63	-28.80 to -22.45	Yes	****	<0.0001
Carboxyl:5000 nm vs. NF:100 nm	-8.125	-11.30 to -4.946	Yes	****	<0.0001
Carboxyl:5000 nm vs. NF:200 nm	-2.800	-5.979 to 0.3786	No	ns	0.1465
Carboxyl:5000 nm vs. NF:500 nm	0.5125	-2.666 to 3.691	No	ns	>0.9999
Carboxyl:5000 nm vs. NF:1000 nm	4.490	1.311 to 7.669	Yes	***	0.0004
Carboxyl:5000 nm vs. NF:5000 nm	3.170	-0.008609 to 6.349	No	ns	0.0513
NF:50 nm vs. NF:100 nm	17.50	14.32 to 20.68	Yes	****	<0.0001
NF:50 nm vs. NF:200 nm	22.83	19.65 to 26.00	Yes	****	<0.0001
NF:50 nm vs. NF:500 nm	26.14	22.96 to 29.32	Yes	****	<0.0001
NF:50 nm vs. NF:1000 nm	30.12	26.94 to 33.29	Yes	****	<0.0001
NF:50 nm vs. NF:5000 nm	28.80	25.62 to 31.97	Yes	****	<0.0001
NF:100 nm vs. NF:200 nm	5.325	2.146 to 8.504	Yes	****	<0.0001
NF:100 nm vs. NF:500 nm	8.638	5.459 to 11.82	Yes	****	<0.0001
NF:100 nm vs. NF:1000 nm	12.62	9.436 to 15.79	Yes	****	<0.0001
NF:100 nm vs. NF:5000 nm	11.30	8.116 to 14.47	Yes	****	<0.0001
NF:200 nm vs. NF:500 nm	3.313	0.1339 to 6.491	Yes	*	0.0328
NF:200 nm vs. NF:1000 nm	7.290	4.111 to 10.47	Yes	****	<0.0001
NF:200 nm vs. NF:5000 nm	5.970	2.791 to 9.149	Yes	****	<0.0001
NF:500 nm vs. NF:1000 nm	3.978	0.7989 to 7.156	Yes	**	0.0032
NF:500 nm vs. NF:5000 nm	2.658	-0.5211 to 5.836	No	ns	0.2087
NF:1000 nm vs. NF:5000 nm	-1.320	-4.499 to 1.859	No	ns	0.9851
