# Supplementary material for: Uptake and toxicity of polystyrene micro/nanoplastics in gastric cells: Effects of particle size and surface functionalization
Source: PLoS One. 2021 Dec 31;16(12):e0260803. doi: 10.1371/journal.pone.0260803 (PMC8719689; doi:10.1371/journal.pone.0260803)
Supplement: S8 Table — (PDF) [file pone.0260803.s020.pdf]

Tukey's multiple comparisons test	Mean Diff.	95.00% CI of diff.	Below threshold?	Summary	Adjusted P Value
50:Amine 4 h vs. 50:Carboxyl 4 h	12.43	8.090 to 16.76	Yes	****	<0.0001
50:Amine 4 h vs. 50:NF 4 h	15.18	10.84 to 19.51	Yes	****	<0.0001
50:Amine 4 h vs. 50:Amine 24 h	12.93	8.590 to 17.26	Yes	****	<0.0001
50:Amine 4 h vs. 50:Carboxyl 24 h	-19.53	-23.86 to -15.19	Yes	****	<0.0001
50:Amine 4 h vs. 50:NF 24 h	-6.100	-10.43 to -1.765	Yes	***	0.0001
50:Amine 4 h vs. 100:Amine 4 h	23.98	19.64 to 28.31	Yes	****	<0.0001
50:Amine 4 h vs. 100:Carboxyl 4 h	22.70	18.37 to 27.04	Yes	****	<0.0001
50:Amine 4 h vs. 100:NF 4 h	21.63	17.29 to 25.96	Yes	****	<0.0001
50:Amine 4 h vs. 100:Amine 24 h	18.50	14.17 to 22.83	Yes	****	<0.0001
50:Amine 4 h vs. 100:Carboxyl 24 h	14.40	10.07 to 18.73	Yes	****	<0.0001
50:Amine 4 h vs. 100:NF 24 h	11.40	7.065 to 15.73	Yes	****	<0.0001
50:Amine 4 h vs. 200:Amine 4 h	20.73	16.39 to 25.06	Yes	****	<0.0001
50:Amine 4 h vs. 200:Carboxyl 4 h	15.93	11.59 to 20.26	Yes	****	<0.0001
50:Amine 4 h vs. 200:NF 4 h	16.55	12.22 to 20.88	Yes	****	<0.0001
50:Amine 4 h vs. 200:Amine 24 h	14.08	9.740 to 18.41	Yes	****	<0.0001
50:Amine 4 h vs. 200:Carboxyl 24 h	19.02	14.68 to 23.35	Yes	****	<0.0001
50:Amine 4 h vs. 200:NF 24 h	16.73	12.39 to 21.06	Yes	****	<0.0001
50:Amine 4 h vs. 500:Amine 4 h	18.82	14.49 to 23.15	Yes	****	<0.0001
50:Amine 4 h vs. 500:Carboxyl 4 h	16.98	12.64 to 21.31	Yes	****	<0.0001
50:Amine 4 h vs. 500:NF 4 h	16.38	12.04 to 20.71	Yes	****	<0.0001
50:Amine 4 h vs. 500:Amine 24 h	14.98	10.64 to 19.31	Yes	****	<0.0001
50:Amine 4 h vs. 500:Carboxyl 24 h	20.87	16.54 to 25.21	Yes	****	<0.0001
50:Amine 4 h vs. 500:NF 24 h	20.04	15.70 to 24.37	Yes	****	<0.0001
50:Amine 4 h vs. 1000:Amine 4 h	17.80	13.47 to 22.13	Yes	****	<0.0001
50:Amine 4 h vs. 1000:Carboxyl 4 h	22.62	18.28 to 26.95	Yes	****	<0.0001
50:Amine 4 h vs. 1000:NF 4 h	25.65	21.31 to 29.98	Yes	****	<0.0001
50:Amine 4 h vs. 1000:Amine 24 h	23.86	19.53 to 28.20	Yes	****	<0.0001
50:Amine 4 h vs. 1000:Carboxyl 24 h	25.00	20.67 to 29.33	Yes	****	<0.0001
50:Amine 4 h vs. 1000:NF 24 h	24.02	19.68 to 28.35	Yes	****	<0.0001
50:Amine 4 h vs. 5000:Amine 4 h	13.90	9.565 to 18.23	Yes	****	<0.0001
50:Amine 4 h vs. 5000:Carboxyl 4 h	20.22	15.89 to 24.55	Yes	****	<0.0001
50:Amine 4 h vs. 5000:NF 4 h	23.88	19.55 to 28.22	Yes	****	<0.0001
50:Amine 4 h vs. 5000:Amine 24 h	17.50	13.17 to 21.83	Yes	****	<0.0001
50:Amine 4 h vs. 5000:Carboxyl 24 h	19.53	15.19 to 23.86	Yes	****	<0.0001
50:Amine 4 h vs. 5000:NF 24 h	22.70	18.36 to 27.03	Yes	****	<0.0001
50:Carboxyl 4 h vs. 50:NF 4 h	2.750	-1.585 to 7.085	No	ns	0.8379
50:Carboxyl 4 h vs. 50:Amine 24 h	0.5000	-3.835 to 4.835	No	ns	>0.9999
50:Carboxyl 4 h vs. 50:Carboxyl 24 h	-31.95	-36.28 to -27.62	Yes	****	<0.0001
50:Carboxyl 4 h vs. 50:NF 24 h	-18.53	-22.86 to -14.19	Yes	****	<0.0001
50:Carboxyl 4 h vs. 100:Amine 4 h	11.55	7.215 to 15.88	Yes	****	<0.0001
50:Carboxyl 4 h vs. 100:Carboxyl 4 h	10.28	5.943 to 14.61	Yes	****	<0.0001
50:Carboxyl 4 h vs. 100:NF 4 h	9.203	4.868 to 13.54	Yes	****	<0.0001
50:Carboxyl 4 h vs. 100:Amine 24 h	6.075	1.740 to 10.41	Yes	***	0.0001
50:Carboxyl 4 h vs. 100:Carboxyl 24 h	1.975	-2.360 to 6.310	No	ns	0.9977
50:Carboxyl 4 h vs. 100:NF 24 h	-1.025	-5.360 to 3.310	No	ns	>0.9999
50:Carboxyl 4 h vs. 200:Amine 4 h	8.300	3.965 to 12.63	Yes	****	<0.0001
50:Carboxyl 4 h vs. 200:Carboxyl 4 h	3.500	-0.8346 to 7.835	No	ns	0.3444
50:Carboxyl 4 h vs. 200:NF 4 h	4.125	-0.2096 to 8.460	No	ns	0.0877
50:Carboxyl 4 h vs. 200:Amine 24 h	1.650	-2.685 to 5.985	No	ns	>0.9999
50:Carboxyl 4 h vs. 200:Carboxyl 24 h	6.590	2.255 to 10.92	Yes	****	<0.0001
50:Carboxyl 4 h vs. 200:NF 24 h	4.300	-0.03463 to 8.635	No	ns	0.0550
50:Carboxyl 4 h vs. 500:Amine 4 h	6.395	2.060 to 10.73	Yes	****	<0.0001
50:Carboxyl 4 h vs. 500:Carboxyl 4 h	4.550	0.2154 to 8.885	Yes	*	0.0268
50:Carboxyl 4 h vs. 500:NF 4 h	3.950	-0.3846 to 8.285	No	ns	0.1352
50:Carboxyl 4 h vs. 500:Amine 24 h	2.550	-1.785 to 6.885	No	ns	0.9214
50:Carboxyl 4 h vs. 500:Carboxyl 24 h	8.448	4.113 to 12.78	Yes	****	<0.0001

50:Carboxyl 4 h vs. 500:NF 24 h	7.613	3.278 to 11.95	Yes	****	<0.0001
50:Carboxyl 4 h vs. 1000:Amine 4 h	5.375	1.040 to 9.710	Yes	**	0.0018
50:Carboxyl 4 h vs. 1000:Carboxyl 4 h	10.19	5.858 to 14.53	Yes	****	<0.0001
50:Carboxyl 4 h vs. 1000:NF 4 h	13.22	8.888 to 17.56	Yes	****	<0.0001
50:Carboxyl 4 h vs. 1000:Amine 24 h	11.44	7.103 to 15.77	Yes	****	<0.0001
50:Carboxyl 4 h vs. 1000:Carboxyl 24 h	12.58	8.240 to 16.91	Yes	****	<0.0001
50:Carboxyl 4 h vs. 1000:NF 24 h	11.59	7.255 to 15.92	Yes	****	<0.0001
50:Carboxyl 4 h vs. 5000:Amine 4 h	1.475	-2.860 to 5.810	No	ns	>0.9999
50:Carboxyl 4 h vs. 5000:Carboxyl 4 h	7.795	3.460 to 12.13	Yes	****	<0.0001
50:Carboxyl 4 h vs. 5000:NF 4 h	11.46	7.123 to 15.79	Yes	****	<0.0001
50:Carboxyl 4 h vs. 5000:Amine 24 h	5.075	0.7404 to 9.410	Yes	**	0.0050
50:Carboxyl 4 h vs. 5000:Carboxyl 24 h	7.100	2.765 to 11.43	Yes	****	<0.0001
50:Carboxyl 4 h vs. 5000:NF 24 h	10.27	5.935 to 14.60	Yes	****	<0.0001
50:NF 4 h vs. 50:Amine 24 h	-2.250	-6.585 to 2.085	No	ns	0.9829
50:NF 4 h vs. 50:Carboxyl 24 h	-34.70	-39.03 to -30.37	Yes	****	<0.0001
50:NF 4 h vs. 50:NF 24 h	-21.28	-25.61 to -16.94	Yes	****	<0.0001
50:NF 4 h vs. 100:Amine 4 h	8.800	4.465 to 13.13	Yes	****	<0.0001
50:NF 4 h vs. 100:Carboxyl 4 h	7.528	3.193 to 11.86	Yes	****	<0.0001
50:NF 4 h vs. 100:NF 4 h	6.453	2.118 to 10.79	Yes	****	<0.0001
50:NF 4 h vs. 100:Amine 24 h	3.325	-1.010 to 7.660	No	ns	0.4585
50:NF 4 h vs. 100:Carboxyl 24 h	-0.7750	-5.110 to 3.560	No	ns	>0.9999
50:NF 4 h vs. 100:NF 24 h	-3.775	-8.110 to 0.5596	No	ns	0.2008
50:NF 4 h vs. 200:Amine 4 h	5.550	1.215 to 9.885	Yes	***	0.0009
50:NF 4 h vs. 200:Carboxyl 4 h	0.7500	-3.585 to 5.085	No	ns	>0.9999
50:NF 4 h vs. 200:NF 4 h	1.375	-2.960 to 5.710	No	ns	>0.9999
50:NF 4 h vs. 200:Amine 24 h	-1.100	-5.435 to 3.235	No	ns	>0.9999
50:NF 4 h vs. 200:Carboxyl 24 h	3.840	-0.4946 to 8.175	No	ns	0.1741
50:NF 4 h vs. 200:NF 24 h	1.550	-2.785 to 5.885	No	ns	>0.9999
50:NF 4 h vs. 500:Amine 4 h	3.645	-0.6896 to 7.980	No	ns	0.2625
50:NF 4 h vs. 500:Carboxyl 4 h	1.800	-2.535 to 6.135	No	ns	0.9996
50:NF 4 h vs. 500:NF 4 h	1.200	-3.135 to 5.535	No	ns	>0.9999
50:NF 4 h vs. 500:Amine 24 h	-0.2000	-4.535 to 4.135	No	ns	>0.9999
50:NF 4 h vs. 500:Carboxyl 24 h	5.698	1.363 to 10.03	Yes	***	0.0005
50:NF 4 h vs. 500:NF 24 h	4.863	0.5279 to 9.197	Yes	*	0.0101
50:NF 4 h vs. 1000:Amine 4 h	2.625	-1.710 to 6.960	No	ns	0.8944
50:NF 4 h vs. 1000:Carboxyl 4 h	7.443	3.108 to 11.78	Yes	****	<0.0001
50:NF 4 h vs. 1000:NF 4 h	10.47	6.138 to 14.81	Yes	****	<0.0001
50:NF 4 h vs. 1000:Amine 24 h	8.688	4.353 to 13.02	Yes	****	<0.0001
50:NF 4 h vs. 1000:Carboxyl 24 h	9.825	5.490 to 14.16	Yes	****	<0.0001
50:NF 4 h vs. 1000:NF 24 h	8.840	4.505 to 13.17	Yes	****	<0.0001
50:NF 4 h vs. 5000:Amine 4 h	-1.275	-5.610 to 3.060	No	ns	>0.9999
50:NF 4 h vs. 5000:Carboxyl 4 h	5.045	0.7104 to 9.380	Yes	**	0.0055
50:NF 4 h vs. 5000:NF 4 h	8.708	4.373 to 13.04	Yes	****	<0.0001
50:NF 4 h vs. 5000:Amine 24 h	2.325	-2.010 to 6.660	No	ns	0.9735
50:NF 4 h vs. 5000:Carboxyl 24 h	4.350	0.01537 to 8.685	Yes	*	0.0479
50:NF 4 h vs. 5000:NF 24 h	7.520	3.185 to 11.85	Yes	****	<0.0001
50:Amine 24 h vs. 50:Carboxyl 24 h	-32.45	-36.78 to -28.12	Yes	****	<0.0001
50:Amine 24 h vs. 50:NF 24 h	-19.03	-23.36 to -14.69	Yes	****	<0.0001
50:Amine 24 h vs. 100:Amine 4 h	11.05	6.715 to 15.38	Yes	****	<0.0001
50:Amine 24 h vs. 100:Carboxyl 4 h	9.778	5.443 to 14.11	Yes	****	<0.0001
50:Amine 24 h vs. 100:NF 4 h	8.703	4.368 to 13.04	Yes	****	<0.0001
50:Amine 24 h vs. 100:Amine 24 h	5.575	1.240 to 9.910	Yes	***	0.0008
50:Amine 24 h vs. 100:Carboxyl 24 h	1.475	-2.860 to 5.810	No	ns	>0.9999
50:Amine 24 h vs. 100:NF 24 h	-1.525	-5.860 to 2.810	No	ns	>0.9999
50:Amine 24 h vs. 200:Amine 4 h	7.800	3.465 to 12.13	Yes	****	<0.0001
50:Amine 24 h vs. 200:Carboxyl 4 h	3.000	-1.335 to 7.335	No	ns	0.6872
50:Amine 24 h vs. 200:NF 4 h	3.625	-0.7096 to 7.960	No	ns	0.2730
50:Amine 24 h vs. 200:Amine 24 h	1.150	-3.185 to 5.485	No	ns	>0.9999

50:Amine 24 h vs. 200:Carboxyl 24 h	6.090	1.755 to 10.42	Yes	***	0.0001
50:Amine 24 h vs. 200:NF 24 h	3.800	-0.5346 to 8.135	No	ns	0.1902
50:Amine 24 h vs. 500:Amine 4 h	5.895	1.560 to 10.23	Yes	***	0.0003
50:Amine 24 h vs. 500:Carboxyl 4 h	4.050	-0.2846 to 8.385	No	ns	0.1060
50:Amine 24 h vs. 500:NF 4 h	3.450	-0.8846 to 7.785	No	ns	0.3755
50:Amine 24 h vs. 500:Amine 24 h	2.050	-2.285 to 6.385	No	ns	0.9958
50:Amine 24 h vs. 500:Carboxyl 24 h	7.948	3.613 to 12.28	Yes	****	<0.0001
50:Amine 24 h vs. 500:NF 24 h	7.113	2.778 to 11.45	Yes	****	<0.0001
50:Amine 24 h vs. 1000:Amine 4 h	4.875	0.5404 to 9.210	Yes	**	0.0097
50:Amine 24 h vs. 1000:Carboxyl 4 h	9.693	5.358 to 14.03	Yes	****	<0.0001
50:Amine 24 h vs. 1000:NF 4 h	12.72	8.388 to 17.06	Yes	****	<0.0001
50:Amine 24 h vs. 1000:Amine 24 h	10.94	6.603 to 15.27	Yes	****	<0.0001
50:Amine 24 h vs. 1000:Carboxyl 24 h	12.08	7.740 to 16.41	Yes	****	<0.0001
50:Amine 24 h vs. 1000:NF 24 h	11.09	6.755 to 15.42	Yes	****	<0.0001
50:Amine 24 h vs. 5000:Amine 4 h	0.9750	-3.360 to 5.310	No	ns	>0.9999
50:Amine 24 h vs. 5000:Carboxyl 4 h	7.295	2.960 to 11.63	Yes	****	<0.0001
50:Amine 24 h vs. 5000:NF 4 h	10.96	6.623 to 15.29	Yes	****	<0.0001
50:Amine 24 h vs. 5000:Amine 24 h	4.575	0.2404 to 8.910	Yes	*	0.0249
50:Amine 24 h vs. 5000:Carboxyl 24 h	6.600	2.265 to 10.93	Yes	****	<0.0001
50:Amine 24 h vs. 5000:NF 24 h	9.770	5.435 to 14.10	Yes	****	<0.0001
50:Carboxyl 24 h vs. 50:NF 24 h	13.43	9.090 to 17.76	Yes	****	<0.0001
50:Carboxyl 24 h vs. 100:Amine 4 h	43.50	39.17 to 47.83	Yes	****	<0.0001
50:Carboxyl 24 h vs. 100:Carboxyl 4 h	42.23	37.89 to 46.56	Yes	****	<0.0001
50:Carboxyl 24 h vs. 100:NF 4 h	41.15	36.82 to 45.49	Yes	****	<0.0001
50:Carboxyl 24 h vs. 100:Amine 24 h	38.03	33.69 to 42.36	Yes	****	<0.0001
50:Carboxyl 24 h vs. 100:Carboxyl 24 h	33.93	29.59 to 38.26	Yes	****	<0.0001
50:Carboxyl 24 h vs. 100:NF 24 h	30.93	26.59 to 35.26	Yes	****	<0.0001
50:Carboxyl 24 h vs. 200:Amine 4 h	40.25	35.92 to 44.58	Yes	****	<0.0001
50:Carboxyl 24 h vs. 200:Carboxyl 4 h	35.45	31.12 to 39.78	Yes	****	<0.0001
50:Carboxyl 24 h vs. 200:NF 4 h	36.08	31.74 to 40.41	Yes	****	<0.0001
50:Carboxyl 24 h vs. 200:Amine 24 h	33.60	29.27 to 37.93	Yes	****	<0.0001
50:Carboxyl 24 h vs. 200:Carboxyl 24 h	38.54	34.21 to 42.87	Yes	****	<0.0001
50:Carboxyl 24 h vs. 200:NF 24 h	36.25	31.92 to 40.58	Yes	****	<0.0001
50:Carboxyl 24 h vs. 500:Amine 4 h	38.35	34.01 to 42.68	Yes	****	<0.0001
50:Carboxyl 24 h vs. 500:Carboxyl 4 h	36.50	32.17 to 40.83	Yes	****	<0.0001
50:Carboxyl 24 h vs. 500:NF 4 h	35.90	31.57 to 40.23	Yes	****	<0.0001
50:Carboxyl 24 h vs. 500:Amine 24 h	34.50	30.17 to 38.83	Yes	****	<0.0001
50:Carboxyl 24 h vs. 500:Carboxyl 24 h	40.40	36.06 to 44.73	Yes	****	<0.0001
50:Carboxyl 24 h vs. 500:NF 24 h	39.56	35.23 to 43.90	Yes	****	<0.0001
50:Carboxyl 24 h vs. 1000:Amine 4 h	37.33	32.99 to 41.66	Yes	****	<0.0001
50:Carboxyl 24 h vs. 1000:Carboxyl 4 h	42.14	37.81 to 46.48	Yes	****	<0.0001
50:Carboxyl 24 h vs. 1000:NF 4 h	45.17	40.84 to 49.51	Yes	****	<0.0001
50:Carboxyl 24 h vs. 1000:Amine 24 h	43.39	39.05 to 47.72	Yes	****	<0.0001
50:Carboxyl 24 h vs. 1000:Carboxyl 24 h	44.53	40.19 to 48.86	Yes	****	<0.0001
50:Carboxyl 24 h vs. 1000:NF 24 h	43.54	39.21 to 47.87	Yes	****	<0.0001
50:Carboxyl 24 h vs. 5000:Amine 4 h	33.43	29.09 to 37.76	Yes	****	<0.0001
50:Carboxyl 24 h vs. 5000:Carboxyl 4 h	39.75	35.41 to 44.08	Yes	****	<0.0001
50:Carboxyl 24 h vs. 5000:NF 4 h	43.41	39.07 to 47.74	Yes	****	<0.0001
50:Carboxyl 24 h vs. 5000:Amine 24 h	37.03	32.69 to 41.36	Yes	****	<0.0001
50:Carboxyl 24 h vs. 5000:Carboxyl 24 h	39.05	34.72 to 43.38	Yes	****	<0.0001
50:Carboxyl 24 h vs. 5000:NF 24 h	42.22	37.89 to 46.55	Yes	****	<0.0001
50:NF 24 h vs. 100:Amine 4 h	30.08	25.74 to 34.41	Yes	****	<0.0001
50:NF 24 h vs. 100:Carboxyl 4 h	28.80	24.47 to 33.14	Yes	****	<0.0001
50:NF 24 h vs. 100:NF 4 h	27.73	23.39 to 32.06	Yes	****	<0.0001
50:NF 24 h vs. 100:Amine 24 h	24.60	20.27 to 28.93	Yes	****	<0.0001
50:NF 24 h vs. 100:Carboxyl 24 h	20.50	16.17 to 24.83	Yes	****	<0.0001
50:NF 24 h vs. 100:NF 24 h	17.50	13.17 to 21.83	Yes	****	<0.0001
50:NF 24 h vs. 200:Amine 4 h	26.83	22.49 to 31.16	Yes	****	<0.0001

50:NF 24 h vs. 200:Carboxyl 4 h	22.03	17.69 to 26.36	Yes	****	<0.0001
50:NF 24 h vs. 200:NF 4 h	22.65	18.32 to 26.98	Yes	****	<0.0001
50:NF 24 h vs. 200:Amine 24 h	20.18	15.84 to 24.51	Yes	****	<0.0001
50:NF 24 h vs. 200:Carboxyl 24 h	25.12	20.78 to 29.45	Yes	****	<0.0001
50:NF 24 h vs. 200:NF 24 h	22.83	18.49 to 27.16	Yes	****	<0.0001
50:NF 24 h vs. 500:Amine 4 h	24.92	20.59 to 29.25	Yes	****	<0.0001
50:NF 24 h vs. 500:Carboxyl 4 h	23.08	18.74 to 27.41	Yes	****	<0.0001
50:NF 24 h vs. 500:NF 4 h	22.48	18.14 to 26.81	Yes	****	<0.0001
50:NF 24 h vs. 500:Amine 24 h	21.08	16.74 to 25.41	Yes	****	<0.0001
50:NF 24 h vs. 500:Carboxyl 24 h	26.97	22.64 to 31.31	Yes	****	<0.0001
50:NF 24 h vs. 500:NF 24 h	26.14	21.80 to 30.47	Yes	****	<0.0001
50:NF 24 h vs. 1000:Amine 4 h	23.90	19.57 to 28.23	Yes	****	<0.0001
50:NF 24 h vs. 1000:Carboxyl 4 h	28.72	24.38 to 33.05	Yes	****	<0.0001
50:NF 24 h vs. 1000:NF 4 h	31.75	27.41 to 36.08	Yes	****	<0.0001
50:NF 24 h vs. 1000:Amine 24 h	29.96	25.63 to 34.30	Yes	****	<0.0001
50:NF 24 h vs. 1000:Carboxyl 24 h	31.10	26.77 to 35.43	Yes	****	<0.0001
50:NF 24 h vs. 1000:NF 24 h	30.12	25.78 to 34.45	Yes	****	<0.0001
50:NF 24 h vs. 5000:Amine 4 h	20.00	15.67 to 24.33	Yes	****	<0.0001
50:NF 24 h vs. 5000:Carboxyl 4 h	26.32	21.99 to 30.65	Yes	****	<0.0001
50:NF 24 h vs. 5000:NF 4 h	29.98	25.65 to 34.32	Yes	****	<0.0001
50:NF 24 h vs. 5000:Amine 24 h	23.60	19.27 to 27.93	Yes	****	<0.0001
50:NF 24 h vs. 5000:Carboxyl 24 h	25.63	21.29 to 29.96	Yes	****	<0.0001
50:NF 24 h vs. 5000:NF 24 h	28.80	24.46 to 33.13	Yes	****	<0.0001
100:Amine 4 h vs. 100:Carboxyl 4 h	-1.273	-5.607 to 3.062	No	ns	>0.9999
100:Amine 4 h vs. 100:NF 4 h	-2.348	-6.682 to 1.987	No	ns	0.9700
100:Amine 4 h vs. 100:Amine 24 h	-5.475	-9.810 to -1.140	Yes	**	0.0012
100:Amine 4 h vs. 100:Carboxyl 24 h	-9.575	-13.91 to -5.240	Yes	****	<0.0001
100:Amine 4 h vs. 100:NF 24 h	-12.58	-16.91 to -8.240	Yes	****	<0.0001
100:Amine 4 h vs. 200:Amine 4 h	-3.250	-7.585 to 1.085	No	ns	0.5110
100:Amine 4 h vs. 200:Carboxyl 4 h	-8.050	-12.38 to -3.715	Yes	****	<0.0001
100:Amine 4 h vs. 200:NF 4 h	-7.425	-11.76 to -3.090	Yes	****	<0.0001
100:Amine 4 h vs. 200:Amine 24 h	-9.900	-14.23 to -5.565	Yes	****	<0.0001
100:Amine 4 h vs. 200:Carboxyl 24 h	-4.960	-9.295 to -0.6254	Yes	**	0.0074
100:Amine 4 h vs. 200:NF 24 h	-7.250	-11.58 to -2.915	Yes	****	<0.0001
100:Amine 4 h vs. 500:Amine 4 h	-5.155	-9.490 to -0.8204	Yes	**	0.0038
100:Amine 4 h vs. 500:Carboxyl 4 h	-7.000	-11.33 to -2.665	Yes	****	<0.0001
100:Amine 4 h vs. 500:NF 4 h	-7.600	-11.93 to -3.265	Yes	****	<0.0001
100:Amine 4 h vs. 500:Amine 24 h	-9.000	-13.33 to -4.665	Yes	****	<0.0001
100:Amine 4 h vs. 500:Carboxyl 24 h	-3.103	-7.437 to 1.232	No	ns	0.6160
100:Amine 4 h vs. 500:NF 24 h	-3.938	-8.272 to 0.3971	No	ns	0.1392
100:Amine 4 h vs. 1000:Amine 4 h	-6.175	-10.51 to -1.840	Yes	****	<0.0001
100:Amine 4 h vs. 1000:Carboxyl 4 h	-1.358	-5.692 to 2.977	No	ns	>0.9999
100:Amine 4 h vs. 1000:NF 4 h	1.673	-2.662 to 6.007	No	ns	>0.9999
100:Amine 4 h vs. 1000:Amine 24 h	-0.1125	-4.447 to 4.222	No	ns	>0.9999
100:Amine 4 h vs. 1000:Carboxyl 24 h	1.025	-3.310 to 5.360	No	ns	>0.9999
100:Amine 4 h vs. 1000:NF 24 h	0.04000	-4.295 to 4.375	No	ns	>0.9999
100:Amine 4 h vs. 5000:Amine 4 h	-10.08	-14.41 to -5.740	Yes	****	<0.0001
100:Amine 4 h vs. 5000:Carboxyl 4 h	-3.755	-8.090 to 0.5796	No	ns	0.2095
100:Amine 4 h vs. 5000:NF 4 h	-0.09250	-4.427 to 4.242	No	ns	>0.9999
100:Amine 4 h vs. 5000:Amine 24 h	-6.475	-10.81 to -2.140	Yes	****	<0.0001
100:Amine 4 h vs. 5000:Carboxyl 24 h	-4.450	-8.785 to -0.1154	Yes	*	0.0360
100:Amine 4 h vs. 5000:NF 24 h	-1.280	-5.615 to 3.055	No	ns	>0.9999
100:Carboxyl 4 h vs. 100:NF 4 h	-1.075	-5.410 to 3.260	No	ns	>0.9999
100:Carboxyl 4 h vs. 100:Amine 24 h	-4.203	-8.537 to 0.1321	No	ns	0.0716
100:Carboxyl 4 h vs. 100:Carboxyl 24 h	-8.303	-12.64 to -3.968	Yes	****	<0.0001
100:Carboxyl 4 h vs. 100:NF 24 h	-11.30	-15.64 to -6.968	Yes	****	<0.0001
100:Carboxyl 4 h vs. 200:Amine 4 h	-1.978	-6.312 to 2.357	No	ns	0.9977
100:Carboxyl 4 h vs. 200:Carboxyl 4 h	-6.778	-11.11 to -2.443	Yes	****	<0.0001

100:Carboxyl 4 h vs. 200:NF 4 h	-6.153	-10.49 to -1.818	Yes	****	<0.0001
100:Carboxyl 4 h vs. 200:Amine 24 h	-8.628	-12.96 to -4.293	Yes	****	<0.0001
100:Carboxyl 4 h vs. 200:Carboxyl 24 h	-3.688	-8.022 to 0.6471	No	ns	0.2411
100:Carboxyl 4 h vs. 200:NF 24 h	-5.978	-10.31 to -1.643	Yes	***	0.0002
100:Carboxyl 4 h vs. 500:Amine 4 h	-3.883	-8.217 to 0.4521	No	ns	0.1582
100:Carboxyl 4 h vs. 500:Carboxyl 4 h	-5.728	-10.06 to -1.393	Yes	***	0.0005
100:Carboxyl 4 h vs. 500:NF 4 h	-6.328	-10.66 to -1.993	Yes	****	<0.0001
100:Carboxyl 4 h vs. 500:Amine 24 h	-7.728	-12.06 to -3.393	Yes	****	<0.0001
100:Carboxyl 4 h vs. 500:Carboxyl 24 h	-1.830	-6.165 to 2.505	No	ns	0.9994
100:Carboxyl 4 h vs. 500:NF 24 h	-2.665	-7.000 to 1.670	No	ns	0.8779
100:Carboxyl 4 h vs. 1000:Amine 4 h	-4.903	-9.237 to -0.5679	Yes	**	0.0089
100:Carboxyl 4 h vs. 1000:Carboxyl 4 h	-0.08500	-4.420 to 4.250	No	ns	>0.9999
100:Carboxyl 4 h vs. 1000:NF 4 h	2.945	-1.390 to 7.280	No	ns	0.7238
100:Carboxyl 4 h vs. 1000:Amine 24 h	1.160	-3.175 to 5.495	No	ns	>0.9999
100:Carboxyl 4 h vs. 1000:Carboxyl 24 h	2.298	-2.037 to 6.632	No	ns	0.9773
100:Carboxyl 4 h vs. 1000:NF 24 h	1.313	-3.022 to 5.647	No	ns	>0.9999
100:Carboxyl 4 h vs. 5000:Amine 4 h	-8.803	-13.14 to -4.468	Yes	****	<0.0001
100:Carboxyl 4 h vs. 5000:Carboxyl 4 h	-2.483	-6.817 to 1.852	No	ns	0.9414
100:Carboxyl 4 h vs. 5000:NF 4 h	1.180	-3.155 to 5.515	No	ns	>0.9999
100:Carboxyl 4 h vs. 5000:Amine 24 h	-5.203	-9.537 to -0.8679	Yes	**	0.0032
100:Carboxyl 4 h vs. 5000:Carboxyl 24 h	-3.178	-7.512 to 1.157	No	ns	0.5626
100:Carboxyl 4 h vs. 5000:NF 24 h	-0.007500	-4.342 to 4.327	No	ns	>0.9999
100:NF 4 h vs. 100:Amine 24 h	-3.128	-7.462 to 1.207	No	ns	0.5982
100:NF 4 h vs. 100:Carboxyl 24 h	-7.228	-11.56 to -2.893	Yes	****	<0.0001
100:NF 4 h vs. 100:NF 24 h	-10.23	-14.56 to -5.893	Yes	****	<0.0001
100:NF 4 h vs. 200:Amine 4 h	-0.9025	-5.237 to 3.432	No	ns	>0.9999
100:NF 4 h vs. 200:Carboxyl 4 h	-5.703	-10.04 to -1.368	Yes	***	0.0005
100:NF 4 h vs. 200:NF 4 h	-5.078	-9.412 to -0.7429	Yes	**	0.0050
100:NF 4 h vs. 200:Amine 24 h	-7.553	-11.89 to -3.218	Yes	****	<0.0001
100:NF 4 h vs. 200:Carboxyl 24 h	-2.613	-6.947 to 1.722	No	ns	0.8993
100:NF 4 h vs. 200:NF 24 h	-4.903	-9.237 to -0.5679	Yes	**	0.0089
100:NF 4 h vs. 500:Amine 4 h	-2.808	-7.142 to 1.527	No	ns	0.8072
100:NF 4 h vs. 500:Carboxyl 4 h	-4.653	-8.987 to -0.3179	Yes	*	0.0197
100:NF 4 h vs. 500:NF 4 h	-5.253	-9.587 to -0.9179	Yes	**	0.0027
100:NF 4 h vs. 500:Amine 24 h	-6.653	-10.99 to -2.318	Yes	****	<0.0001
100:NF 4 h vs. 500:Carboxyl 24 h	-0.7550	-5.090 to 3.580	No	ns	>0.9999
100:NF 4 h vs. 500:NF 24 h	-1.590	-5.925 to 2.745	No	ns	>0.9999
100:NF 4 h vs. 1000:Amine 4 h	-3.828	-8.162 to 0.5071	No	ns	0.1790
100:NF 4 h vs. 1000:Carboxyl 4 h	0.9900	-3.345 to 5.325	No	ns	>0.9999
100:NF 4 h vs. 1000:NF 4 h	4.020	-0.3146 to 8.355	No	ns	0.1142
100:NF 4 h vs. 1000:Amine 24 h	2.235	-2.100 to 6.570	No	ns	0.9844
100:NF 4 h vs. 1000:Carboxyl 24 h	3.373	-0.9621 to 7.707	No	ns	0.4262
100:NF 4 h vs. 1000:NF 24 h	2.388	-1.947 to 6.722	No	ns	0.9630
100:NF 4 h vs. 5000:Amine 4 h	-7.728	-12.06 to -3.393	Yes	****	<0.0001
100:NF 4 h vs. 5000:Carboxyl 4 h	-1.408	-5.742 to 2.927	No	ns	>0.9999
100:NF 4 h vs. 5000:NF 4 h	2.255	-2.080 to 6.590	No	ns	0.9824
100:NF 4 h vs. 5000:Amine 24 h	-4.128	-8.462 to 0.2071	No	ns	0.0871
100:NF 4 h vs. 5000:Carboxyl 24 h	-2.103	-6.437 to 2.232	No	ns	0.9937
100:NF 4 h vs. 5000:NF 24 h	1.068	-3.267 to 5.402	No	ns	>0.9999
100:Amine 24 h vs. 100:Carboxyl 24 h	-4.100	-8.435 to 0.2346	No	ns	0.0935
100:Amine 24 h vs. 100:NF 24 h	-7.100	-11.43 to -2.765	Yes	****	<0.0001
100:Amine 24 h vs. 200:Amine 4 h	2.225	-2.110 to 6.560	No	ns	0.9854
100:Amine 24 h vs. 200:Carboxyl 4 h	-2.575	-6.910 to 1.760	No	ns	0.9130
100:Amine 24 h vs. 200:NF 4 h	-1.950	-6.285 to 2.385	No	ns	0.9982
100:Amine 24 h vs. 200:Amine 24 h	-4.425	-8.760 to -0.09037	Yes	*	0.0387
100:Amine 24 h vs. 200:Carboxyl 24 h	0.5150	-3.820 to 4.850	No	ns	>0.9999
100:Amine 24 h vs. 200:NF 24 h	-1.775	-6.110 to 2.560	No	ns	0.9997

100:Amine 24 h vs. 500:Amine 4 h	0.3200	-4.015 to 4.655	No	ns	>0.9999
100:Amine 24 h vs. 500:Carboxyl 4 h	-1.525	-5.860 to 2.810	No	ns	>0.9999
100:Amine 24 h vs. 500:NF 4 h	-2.125	-6.460 to 2.210	No	ns	0.9926
100:Amine 24 h vs. 500:Amine 24 h	-3.525	-7.860 to 0.8096	No	ns	0.3294
100:Amine 24 h vs. 500:Carboxyl 24 h	2.373	-1.962 to 6.707	No	ns	0.9657
100:Amine 24 h vs. 500:NF 24 h	1.538	-2.797 to 5.872	No	ns	>0.9999
100:Amine 24 h vs. 1000:Amine 4 h	-0.7000	-5.035 to 3.635	No	ns	>0.9999
100:Amine 24 h vs. 1000:Carboxyl 4 h	4.118	-0.2171 to 8.452	No	ns	0.0894
100:Amine 24 h vs. 1000:NF 4 h	7.148	2.813 to 11.48	Yes	****	<0.0001
100:Amine 24 h vs. 1000:Amine 24 h	5.363	1.028 to 9.697	Yes	**	0.0018
100:Amine 24 h vs. 1000:Carboxyl 24 h	6.500	2.165 to 10.83	Yes	****	<0.0001
100:Amine 24 h vs. 1000:NF 24 h	5.515	1.180 to 9.850	Yes	**	0.0011
100:Amine 24 h vs. 5000:Amine 4 h	-4.600	-8.935 to -0.2654	Yes	*	0.0231
100:Amine 24 h vs. 5000:Carboxyl 4 h	1.720	-2.615 to 6.055	No	ns	0.9998
100:Amine 24 h vs. 5000:NF 4 h	5.383	1.048 to 9.717	Yes	**	0.0017
100:Amine 24 h vs. 5000:Amine 24 h	-1.000	-5.335 to 3.335	No	ns	>0.9999
100:Amine 24 h vs. 5000:Carboxyl 24 h	1.025	-3.310 to 5.360	No	ns	>0.9999
100:Amine 24 h vs. 5000:NF 24 h	4.195	-0.1396 to 8.530	No	ns	0.0731
100:Carboxyl 24 h vs. 100:NF 24 h	-3.000	-7.335 to 1.335	No	ns	0.6872
100:Carboxyl 24 h vs. 200:Amine 4 h	6.325	1.990 to 10.66	Yes	****	<0.0001
100:Carboxyl 24 h vs. 200:Carboxyl 4 h	1.525	-2.810 to 5.860	No	ns	>0.9999
100:Carboxyl 24 h vs. 200:NF 4 h	2.150	-2.185 to 6.485	No	ns	0.9911
100:Carboxyl 24 h vs. 200:Amine 24 h	-0.3250	-4.660 to 4.010	No	ns	>0.9999
100:Carboxyl 24 h vs. 200:Carboxyl 24 h	4.615	0.2804 to 8.950	Yes	*	0.0221
100:Carboxyl 24 h vs. 200:NF 24 h	2.325	-2.010 to 6.660	No	ns	0.9735
100:Carboxyl 24 h vs. 500:Amine 4 h	4.420	0.08537 to 8.755	Yes	*	0.0393
100:Carboxyl 24 h vs. 500:Carboxyl 4 h	2.575	-1.760 to 6.910	No	ns	0.9130
100:Carboxyl 24 h vs. 500:NF 4 h	1.975	-2.360 to 6.310	No	ns	0.9977
100:Carboxyl 24 h vs. 500:Amine 24 h	0.5750	-3.760 to 4.910	No	ns	>0.9999
100:Carboxyl 24 h vs. 500:Carboxyl 24 h	6.473	2.138 to 10.81	Yes	****	<0.0001
100:Carboxyl 24 h vs. 500:NF 24 h	5.638	1.303 to 9.972	Yes	***	0.0007
100:Carboxyl 24 h vs. 1000:Amine 4 h	3.400	-0.9346 to 7.735	No	ns	0.4079
100:Carboxyl 24 h vs. 1000:Carboxyl 4 h	8.218	3.883 to 12.55	Yes	****	<0.0001
100:Carboxyl 24 h vs. 1000:NF 4 h	11.25	6.913 to 15.58	Yes	****	<0.0001
100:Carboxyl 24 h vs. 1000:Amine 24 h	9.463	5.128 to 13.80	Yes	****	<0.0001
100:Carboxyl 24 h vs. 1000:Carboxyl 24 h	10.60	6.265 to 14.93	Yes	****	<0.0001
100:Carboxyl 24 h vs. 1000:NF 24 h	9.615	5.280 to 13.95	Yes	****	<0.0001
100:Carboxyl 24 h vs. 5000:Amine 4 h	-0.5000	-4.835 to 3.835	No	ns	>0.9999
100:Carboxyl 24 h vs. 5000:Carboxyl 4 h	5.820	1.485 to 10.15	Yes	***	0.0003
100:Carboxyl 24 h vs. 5000:NF 4 h	9.483	5.148 to 13.82	Yes	****	<0.0001
100:Carboxyl 24 h vs. 5000:Amine 24 h	3.100	-1.235 to 7.435	No	ns	0.6177
100:Carboxyl 24 h vs. 5000:Carboxyl 24 h	5.125	0.7904 to 9.460	Yes	**	0.0042
100:Carboxyl 24 h vs. 5000:NF 24 h	8.295	3.960 to 12.63	Yes	****	<0.0001
100:NF 24 h vs. 200:Amine 4 h	9.325	4.990 to 13.66	Yes	****	<0.0001
100:NF 24 h vs. 200:Carboxyl 4 h	4.525	0.1904 to 8.860	Yes	*	0.0289
100:NF 24 h vs. 200:NF 4 h	5.150	0.8154 to 9.485	Yes	**	0.0039
100:NF 24 h vs. 200:Amine 24 h	2.675	-1.660 to 7.010	No	ns	0.8735
100:NF 24 h vs. 200:Carboxyl 24 h	7.615	3.280 to 11.95	Yes	****	<0.0001
100:NF 24 h vs. 200:NF 24 h	5.325	0.9904 to 9.660	Yes	**	0.0021
100:NF 24 h vs. 500:Amine 4 h	7.420	3.085 to 11.75	Yes	****	<0.0001
100:NF 24 h vs. 500:Carboxyl 4 h	5.575	1.240 to 9.910	Yes	***	0.0008
100:NF 24 h vs. 500:NF 4 h	4.975	0.6404 to 9.310	Yes	**	0.0070
100:NF 24 h vs. 500:Amine 24 h	3.575	-0.7596 to 7.910	No	ns	0.3004
100:NF 24 h vs. 500:Carboxyl 24 h	9.473	5.138 to 13.81	Yes	****	<0.0001
100:NF 24 h vs. 500:NF 24 h	8.638	4.303 to 12.97	Yes	****	<0.0001
100:NF 24 h vs. 1000:Amine 4 h	6.400	2.065 to 10.73	Yes	****	<0.0001
100:NF 24 h vs. 1000:Carboxyl 4 h	11.22	6.883 to 15.55	Yes	****	<0.0001
100:NF 24 h vs. 1000:NF 4 h	14.25	9.913 to 18.58	Yes	****	<0.0001

100:NF 24 h vs. 1000:Amine 24 h	12.46	8.128 to 16.80	Yes	****	<0.0001
100:NF 24 h vs. 1000:Carboxyl 24 h	13.60	9.265 to 17.93	Yes	****	<0.0001
100:NF 24 h vs. 1000:NF 24 h	12.62	8.280 to 16.95	Yes	****	<0.0001
100:NF 24 h vs. 5000:Amine 4 h	2.500	-1.835 to 6.835	No	ns	0.9366
100:NF 24 h vs. 5000:Carboxyl 4 h	8.820	4.485 to 13.15	Yes	****	<0.0001
100:NF 24 h vs. 5000:NF 4 h	12.48	8.148 to 16.82	Yes	****	<0.0001
100:NF 24 h vs. 5000:Amine 24 h	6.100	1.765 to 10.43	Yes	***	0.0001
100:NF 24 h vs. 5000:Carboxyl 24 h	8.125	3.790 to 12.46	Yes	****	<0.0001
100:NF 24 h vs. 5000:NF 24 h	11.30	6.960 to 15.63	Yes	****	<0.0001
200:Amine 4 h vs. 200:Carboxyl 4 h	-4.800	-9.135 to -0.4654	Yes	*	0.0124
200:Amine 4 h vs. 200:NF 4 h	-4.175	-8.510 to 0.1596	No	ns	0.0770
200:Amine 4 h vs. 200:Amine 24 h	-6.650	-10.98 to -2.315	Yes	****	<0.0001
200:Amine 4 h vs. 200:Carboxyl 24 h	-1.710	-6.045 to 2.625	No	ns	0.9998
200:Amine 4 h vs. 200:NF 24 h	-4.000	-8.335 to 0.3346	No	ns	0.1199
200:Amine 4 h vs. 500:Amine 4 h	-1.905	-6.240 to 2.430	No	ns	0.9988
200:Amine 4 h vs. 500:Carboxyl 4 h	-3.750	-8.085 to 0.5846	No	ns	0.2118
200:Amine 4 h vs. 500:NF 4 h	-4.350	-8.685 to -0.01537	Yes	*	0.0479
200:Amine 4 h vs. 500:Amine 24 h	-5.750	-10.08 to -1.415	Yes	***	0.0004
200:Amine 4 h vs. 500:Carboxyl 24 h	0.1475	-4.187 to 4.482	No	ns	>0.9999
200:Amine 4 h vs. 500:NF 24 h	-0.6875	-5.022 to 3.647	No	ns	>0.9999
200:Amine 4 h vs. 1000:Amine 4 h	-2.925	-7.260 to 1.410	No	ns	0.7367
200:Amine 4 h vs. 1000:Carboxyl 4 h	1.893	-2.442 to 6.227	No	ns	0.9989
200:Amine 4 h vs. 1000:NF 4 h	4.923	0.5879 to 9.257	Yes	**	0.0083
200:Amine 4 h vs. 1000:Amine 24 h	3.138	-1.197 to 7.472	No	ns	0.5911
200:Amine 4 h vs. 1000:Carboxyl 24 h	4.275	-0.05963 to 8.610	No	ns	0.0589
200:Amine 4 h vs. 1000:NF 24 h	3.290	-1.045 to 7.625	No	ns	0.4828
200:Amine 4 h vs. 5000:Amine 4 h	-6.825	-11.16 to -2.490	Yes	****	<0.0001
200:Amine 4 h vs. 5000:Carboxyl 4 h	-0.5050	-4.840 to 3.830	No	ns	>0.9999
200:Amine 4 h vs. 5000:NF 4 h	3.158	-1.177 to 7.492	No	ns	0.5768
200:Amine 4 h vs. 5000:Amine 24 h	-3.225	-7.560 to 1.110	No	ns	0.5287
200:Amine 4 h vs. 5000:Carboxyl 24 h	-1.200	-5.535 to 3.135	No	ns	>0.9999
200:Amine 4 h vs. 5000:NF 24 h	1.970	-2.365 to 6.305	No	ns	0.9978
200:Carboxyl 4 h vs. 200:NF 4 h	0.6250	-3.710 to 4.960	No	ns	>0.9999
200:Carboxyl 4 h vs. 200:Amine 24 h	-1.850	-6.185 to 2.485	No	ns	0.9993
200:Carboxyl 4 h vs. 200:Carboxyl 24 h	3.090	-1.245 to 7.425	No	ns	0.6248
200:Carboxyl 4 h vs. 200:NF 24 h	0.8000	-3.535 to 5.135	No	ns	>0.9999
200:Carboxyl 4 h vs. 500:Amine 4 h	2.895	-1.440 to 7.230	No	ns	0.7556
200:Carboxyl 4 h vs. 500:Carboxyl 4 h	1.050	-3.285 to 5.385	No	ns	>0.9999
200:Carboxyl 4 h vs. 500:NF 4 h	0.4500	-3.885 to 4.785	No	ns	>0.9999
200:Carboxyl 4 h vs. 500:Amine 24 h	-0.9500	-5.285 to 3.385	No	ns	>0.9999
200:Carboxyl 4 h vs. 500:Carboxyl 24 h	4.948	0.6129 to 9.282	Yes	**	0.0077
200:Carboxyl 4 h vs. 500:NF 24 h	4.113	-0.2221 to 8.447	No	ns	0.0906
200:Carboxyl 4 h vs. 1000:Amine 4 h	1.875	-2.460 to 6.210	No	ns	0.9991
200:Carboxyl 4 h vs. 1000:Carboxyl 4 h	6.693	2.358 to 11.03	Yes	****	<0.0001
200:Carboxyl 4 h vs. 1000:NF 4 h	9.723	5.388 to 14.06	Yes	****	<0.0001
200:Carboxyl 4 h vs. 1000:Amine 24 h	7.938	3.603 to 12.27	Yes	****	<0.0001
200:Carboxyl 4 h vs. 1000:Carboxyl 24 h	9.075	4.740 to 13.41	Yes	****	<0.0001
200:Carboxyl 4 h vs. 1000:NF 24 h	8.090	3.755 to 12.42	Yes	****	<0.0001
200:Carboxyl 4 h vs. 5000:Amine 4 h	-2.025	-6.360 to 2.310	No	ns	0.9965
200:Carboxyl 4 h vs. 5000:Carboxyl 4 h	4.295	-0.03963 to 8.630	No	ns	0.0558
200:Carboxyl 4 h vs. 5000:NF 4 h	7.958	3.623 to 12.29	Yes	****	<0.0001
200:Carboxyl 4 h vs. 5000:Amine 24 h	1.575	-2.760 to 5.910	No	ns	>0.9999
200:Carboxyl 4 h vs. 5000:Carboxyl 24 h	3.600	-0.7346 to 7.935	No	ns	0.2865
200:Carboxyl 4 h vs. 5000:NF 24 h	6.770	2.435 to 11.10	Yes	****	<0.0001
200:NF 4 h vs. 200:Amine 24 h	-2.475	-6.810 to 1.860	No	ns	0.9434
200:NF 4 h vs. 200:Carboxyl 24 h	2.465	-1.870 to 6.800	No	ns	0.9459
200:NF 4 h vs. 200:NF 24 h	0.1750	-4.160 to 4.510	No	ns	>0.9999
200:NF 4 h vs. 500:Amine 4 h	2.270	-2.065 to 6.605	No	ns	0.9807

200:NF 4 h vs. 500:Carboxyl 4 h	0.4250	-3.910 to 4.760	No	ns	>0.9999
200:NF 4 h vs. 500:NF 4 h	-0.1750	-4.510 to 4.160	No	ns	>0.9999
200:NF 4 h vs. 500:Amine 24 h	-1.575	-5.910 to 2.760	No	ns	>0.9999
200:NF 4 h vs. 500:Carboxyl 24 h	4.323	-0.01213 to 8.657	No	ns	0.0517
200:NF 4 h vs. 500:NF 24 h	3.488	-0.8471 to 7.822	No	ns	0.3520
200:NF 4 h vs. 1000:Amine 4 h	1.250	-3.085 to 5.585	No	ns	>0.9999
200:NF 4 h vs. 1000:Carboxyl 4 h	6.068	1.733 to 10.40	Yes	***	0.0001
200:NF 4 h vs. 1000:NF 4 h	9.098	4.763 to 13.43	Yes	****	<0.0001
200:NF 4 h vs. 1000:Amine 24 h	7.313	2.978 to 11.65	Yes	****	<0.0001
200:NF 4 h vs. 1000:Carboxyl 24 h	8.450	4.115 to 12.78	Yes	****	<0.0001
200:NF 4 h vs. 1000:NF 24 h	7.465	3.130 to 11.80	Yes	****	<0.0001
200:NF 4 h vs. 5000:Amine 4 h	-2.650	-6.985 to 1.685	No	ns	0.8843
200:NF 4 h vs. 5000:Carboxyl 4 h	3.670	-0.6646 to 8.005	No	ns	0.2498
200:NF 4 h vs. 5000:NF 4 h	7.333	2.998 to 11.67	Yes	****	<0.0001
200:NF 4 h vs. 5000:Amine 24 h	0.9500	-3.385 to 5.285	No	ns	>0.9999
200:NF 4 h vs. 5000:Carboxyl 24 h	2.975	-1.360 to 7.310	No	ns	0.7040
200:NF 4 h vs. 5000:NF 24 h	6.145	1.810 to 10.48	Yes	****	<0.0001
200:Amine 24 h vs. 200:Carboxyl 24 h	4.940	0.6054 to 9.275	Yes	**	0.0079
200:Amine 24 h vs. 200:NF 24 h	2.650	-1.685 to 6.985	No	ns	0.8843
200:Amine 24 h vs. 500:Amine 4 h	4.745	0.4104 to 9.080	Yes	*	0.0147
200:Amine 24 h vs. 500:Carboxyl 4 h	2.900	-1.435 to 7.235	No	ns	0.7525
200:Amine 24 h vs. 500:NF 4 h	2.300	-2.035 to 6.635	No	ns	0.9770
200:Amine 24 h vs. 500:Amine 24 h	0.9000	-3.435 to 5.235	No	ns	>0.9999
200:Amine 24 h vs. 500:Carboxyl 24 h	6.798	2.463 to 11.13	Yes	****	<0.0001
200:Amine 24 h vs. 500:NF 24 h	5.963	1.628 to 10.30	Yes	***	0.0002
200:Amine 24 h vs. 1000:Amine 4 h	3.725	-0.6096 to 8.060	No	ns	0.2232
200:Amine 24 h vs. 1000:Carboxyl 4 h	8.543	4.208 to 12.88	Yes	****	<0.0001
200:Amine 24 h vs. 1000:NF 4 h	11.57	7.238 to 15.91	Yes	****	<0.0001
200:Amine 24 h vs. 1000:Amine 24 h	9.788	5.453 to 14.12	Yes	****	<0.0001
200:Amine 24 h vs. 1000:Carboxyl 24 h	10.93	6.590 to 15.26	Yes	****	<0.0001
200:Amine 24 h vs. 1000:NF 24 h	9.940	5.605 to 14.27	Yes	****	<0.0001
200:Amine 24 h vs. 5000:Amine 4 h	-0.1750	-4.510 to 4.160	No	ns	>0.9999
200:Amine 24 h vs. 5000:Carboxyl 4 h	6.145	1.810 to 10.48	Yes	****	<0.0001
200:Amine 24 h vs. 5000:NF 4 h	9.808	5.473 to 14.14	Yes	****	<0.0001
200:Amine 24 h vs. 5000:Amine 24 h	3.425	-0.9096 to 7.760	No	ns	0.3916
200:Amine 24 h vs. 5000:Carboxyl 24 h	5.450	1.115 to 9.785	Yes	**	0.0013
200:Amine 24 h vs. 5000:NF 24 h	8.620	4.285 to 12.95	Yes	****	<0.0001
200:Carboxyl 24 h vs. 200:NF 24 h	-2.290	-6.625 to 2.045	No	ns	0.9783
200:Carboxyl 24 h vs. 500:Amine 4 h	-0.1950	-4.530 to 4.140	No	ns	>0.9999
200:Carboxyl 24 h vs. 500:Carboxyl 4 h	-2.040	-6.375 to 2.295	No	ns	0.9961
200:Carboxyl 24 h vs. 500:NF 4 h	-2.640	-6.975 to 1.695	No	ns	0.8884
200:Carboxyl 24 h vs. 500:Amine 24 h	-4.040	-8.375 to 0.2946	No	ns	0.1087
200:Carboxyl 24 h vs. 500:Carboxyl 24 h	1.858	-2.477 to 6.192	No	ns	0.9992
200:Carboxyl 24 h vs. 500:NF 24 h	1.023	-3.312 to 5.357	No	ns	>0.9999
200:Carboxyl 24 h vs. 1000:Amine 4 h	-1.215	-5.550 to 3.120	No	ns	>0.9999
200:Carboxyl 24 h vs. 1000:Carboxyl 4 h	3.603	-0.7321 to 7.937	No	ns	0.2851
200:Carboxyl 24 h vs. 1000:NF 4 h	6.633	2.298 to 10.97	Yes	****	<0.0001
200:Carboxyl 24 h vs. 1000:Amine 24 h	4.848	0.5129 to 9.182	Yes	*	0.0106
200:Carboxyl 24 h vs. 1000:Carboxyl 24 h	5.985	1.650 to 10.32	Yes	***	0.0002
200:Carboxyl 24 h vs. 1000:NF 24 h	5.000	0.6654 to 9.335	Yes	**	0.0064
200:Carboxyl 24 h vs. 5000:Amine 4 h	-5.115	-9.450 to -0.7804	Yes	**	0.0044
200:Carboxyl 24 h vs. 5000:Carboxyl 4 h	1.205	-3.130 to 5.540	No	ns	>0.9999
200:Carboxyl 24 h vs. 5000:NF 4 h	4.868	0.5329 to 9.202	Yes	**	0.0100
200:Carboxyl 24 h vs. 5000:Amine 24 h	-1.515	-5.850 to 2.820	No	ns	>0.9999
200:Carboxyl 24 h vs. 5000:Carboxyl 24 h	0.5100	-3.825 to 4.845	No	ns	>0.9999
200:Carboxyl 24 h vs. 5000:NF 24 h	3.680	-0.6546 to 8.015	No	ns	0.2448
200:NF 24 h vs. 500:Amine 4 h	2.095	-2.240 to 6.430	No	ns	0.9940
200:NF 24 h vs. 500:Carboxyl 4 h	0.2500	-4.085 to 4.585	No	ns	>0.9999

200:NF 24 h vs. 500:NF 4 h	-0.3500	-4.685 to 3.985	No	ns	>0.9999
200:NF 24 h vs. 500:Amine 24 h	-1.750	-6.085 to 2.585	No	ns	0.9998
200:NF 24 h vs. 500:Carboxyl 24 h	4.148	-0.1871 to 8.482	No	ns	0.0828
200:NF 24 h vs. 500:NF 24 h	3.313	-1.022 to 7.647	No	ns	0.4672
200:NF 24 h vs. 1000:Amine 4 h	1.075	-3.260 to 5.410	No	ns	>0.9999
200:NF 24 h vs. 1000:Carboxyl 4 h	5.893	1.558 to 10.23	Yes	***	0.0003
200:NF 24 h vs. 1000:NF 4 h	8.923	4.588 to 13.26	Yes	****	<0.0001
200:NF 24 h vs. 1000:Amine 24 h	7.138	2.803 to 11.47	Yes	****	<0.0001
200:NF 24 h vs. 1000:Carboxyl 24 h	8.275	3.940 to 12.61	Yes	****	<0.0001
200:NF 24 h vs. 1000:NF 24 h	7.290	2.955 to 11.62	Yes	****	<0.0001
200:NF 24 h vs. 5000:Amine 4 h	-2.825	-7.160 to 1.510	No	ns	0.7973
200:NF 24 h vs. 5000:Carboxyl 4 h	3.495	-0.8396 to 7.830	No	ns	0.3474
200:NF 24 h vs. 5000:NF 4 h	7.158	2.823 to 11.49	Yes	****	<0.0001
200:NF 24 h vs. 5000:Amine 24 h	0.7750	-3.560 to 5.110	No	ns	>0.9999
200:NF 24 h vs. 5000:Carboxyl 24 h	2.800	-1.535 to 7.135	No	ns	0.8114
200:NF 24 h vs. 5000:NF 24 h	5.970	1.635 to 10.30	Yes	***	0.0002
500:Amine 4 h vs. 500:Carboxyl 4 h	-1.845	-6.180 to 2.490	No	ns	0.9993
500:Amine 4 h vs. 500:NF 4 h	-2.445	-6.780 to 1.890	No	ns	0.9508
500:Amine 4 h vs. 500:Amine 24 h	-3.845	-8.180 to 0.4896	No	ns	0.1722
500:Amine 4 h vs. 500:Carboxyl 24 h	2.053	-2.282 to 6.387	No	ns	0.9957
500:Amine 4 h vs. 500:NF 24 h	1.218	-3.117 to 5.552	No	ns	>0.9999
500:Amine 4 h vs. 1000:Amine 4 h	-1.020	-5.355 to 3.315	No	ns	>0.9999
500:Amine 4 h vs. 1000:Carboxyl 4 h	3.798	-0.5371 to 8.132	No	ns	0.1912
500:Amine 4 h vs. 1000:NF 4 h	6.828	2.493 to 11.16	Yes	****	<0.0001
500:Amine 4 h vs. 1000:Amine 24 h	5.043	0.7079 to 9.377	Yes	**	0.0056
500:Amine 4 h vs. 1000:Carboxyl 24 h	6.180	1.845 to 10.51	Yes	****	<0.0001
500:Amine 4 h vs. 1000:NF 24 h	5.195	0.8604 to 9.530	Yes	**	0.0033
500:Amine 4 h vs. 5000:Amine 4 h	-4.920	-9.255 to -0.5854	Yes	**	0.0084
500:Amine 4 h vs. 5000:Carboxyl 4 h	1.400	-2.935 to 5.735	No	ns	>0.9999
500:Amine 4 h vs. 5000:NF 4 h	5.063	0.7279 to 9.397	Yes	**	0.0052
500:Amine 4 h vs. 5000:Amine 24 h	-1.320	-5.655 to 3.015	No	ns	>0.9999
500:Amine 4 h vs. 5000:Carboxyl 24 h	0.7050	-3.630 to 5.040	No	ns	>0.9999
500:Amine 4 h vs. 5000:NF 24 h	3.875	-0.4596 to 8.210	No	ns	0.1609
500:Carboxyl 4 h vs. 500:NF 4 h	-0.6000	-4.935 to 3.735	No	ns	>0.9999
500:Carboxyl 4 h vs. 500:Amine 24 h	-2.000	-6.335 to 2.335	No	ns	0.9972
500:Carboxyl 4 h vs. 500:Carboxyl 24 h	3.898	-0.4371 to 8.232	No	ns	0.1528
500:Carboxyl 4 h vs. 500:NF 24 h	3.063	-1.272 to 7.397	No	ns	0.6441
500:Carboxyl 4 h vs. 1000:Amine 4 h	0.8250	-3.510 to 5.160	No	ns	>0.9999
500:Carboxyl 4 h vs. 1000:Carboxyl 4 h	5.643	1.308 to 9.977	Yes	***	0.0007
500:Carboxyl 4 h vs. 1000:NF 4 h	8.673	4.338 to 13.01	Yes	****	<0.0001
500:Carboxyl 4 h vs. 1000:Amine 24 h	6.888	2.553 to 11.22	Yes	****	<0.0001
500:Carboxyl 4 h vs. 1000:Carboxyl 24 h	8.025	3.690 to 12.36	Yes	****	<0.0001
500:Carboxyl 4 h vs. 1000:NF 24 h	7.040	2.705 to 11.37	Yes	****	<0.0001
500:Carboxyl 4 h vs. 5000:Amine 4 h	-3.075	-7.410 to 1.260	No	ns	0.6354
500:Carboxyl 4 h vs. 5000:Carboxyl 4 h	3.245	-1.090 to 7.580	No	ns	0.5145
500:Carboxyl 4 h vs. 5000:NF 4 h	6.908	2.573 to 11.24	Yes	****	<0.0001
500:Carboxyl 4 h vs. 5000:Amine 24 h	0.5250	-3.810 to 4.860	No	ns	>0.9999
500:Carboxyl 4 h vs. 5000:Carboxyl 24 h	2.550	-1.785 to 6.885	No	ns	0.9214
500:Carboxyl 4 h vs. 5000:NF 24 h	5.720	1.385 to 10.05	Yes	***	0.0005
500:NF 4 h vs. 500:Amine 24 h	-1.400	-5.735 to 2.935	No	ns	>0.9999
500:NF 4 h vs. 500:Carboxyl 24 h	4.498	0.1629 to 8.832	Yes	*	0.0314
500:NF 4 h vs. 500:NF 24 h	3.663	-0.6721 to 7.997	No	ns	0.2536
500:NF 4 h vs. 1000:Amine 4 h	1.425	-2.910 to 5.760	No	ns	>0.9999
500:NF 4 h vs. 1000:Carboxyl 4 h	6.243	1.908 to 10.58	Yes	****	<0.0001
500:NF 4 h vs. 1000:NF 4 h	9.273	4.938 to 13.61	Yes	****	<0.0001
500:NF 4 h vs. 1000:Amine 24 h	7.488	3.153 to 11.82	Yes	****	<0.0001
500:NF 4 h vs. 1000:Carboxyl 24 h	8.625	4.290 to 12.96	Yes	****	<0.0001
500:NF 4 h vs. 1000:NF 24 h	7.640	3.305 to 11.97	Yes	****	<0.0001

500:NF 4 h vs. 5000:Amine 4 h	-2.475	-6.810 to 1.860	No	ns	0.9434
500:NF 4 h vs. 5000:Carboxyl 4 h	3.845	-0.4896 to 8.180	No	ns	0.1722
500:NF 4 h vs. 5000:NF 4 h	7.508	3.173 to 11.84	Yes	****	<0.0001
500:NF 4 h vs. 5000:Amine 24 h	1.125	-3.210 to 5.460	No	ns	>0.9999
500:NF 4 h vs. 5000:Carboxyl 24 h	3.150	-1.185 to 7.485	No	ns	0.5822
500:NF 4 h vs. 5000:NF 24 h	6.320	1.985 to 10.65	Yes	****	<0.0001
500:Amine 24 h vs. 500:Carboxyl 24 h	5.898	1.563 to 10.23	Yes	***	0.0003
500:Amine 24 h vs. 500:NF 24 h	5.063	0.7279 to 9.397	Yes	**	0.0052
500:Amine 24 h vs. 1000:Amine 4 h	2.825	-1.510 to 7.160	No	ns	0.7973
500:Amine 24 h vs. 1000:Carboxyl 4 h	7.643	3.308 to 11.98	Yes	****	<0.0001
500:Amine 24 h vs. 1000:NF 4 h	10.67	6.338 to 15.01	Yes	****	<0.0001
500:Amine 24 h vs. 1000:Amine 24 h	8.888	4.553 to 13.22	Yes	****	<0.0001
500:Amine 24 h vs. 1000:Carboxyl 24 h	10.03	5.690 to 14.36	Yes	****	<0.0001
500:Amine 24 h vs. 1000:NF 24 h	9.040	4.705 to 13.37	Yes	****	<0.0001
500:Amine 24 h vs. 5000:Amine 4 h	-1.075	-5.410 to 3.260	No	ns	>0.9999
500:Amine 24 h vs. 5000:Carboxyl 4 h	5.245	0.9104 to 9.580	Yes	**	0.0028
500:Amine 24 h vs. 5000:NF 4 h	8.908	4.573 to 13.24	Yes	****	<0.0001
500:Amine 24 h vs. 5000:Amine 24 h	2.525	-1.810 to 6.860	No	ns	0.9293
500:Amine 24 h vs. 5000:Carboxyl 24 h	4.550	0.2154 to 8.885	Yes	*	0.0268
500:Amine 24 h vs. 5000:NF 24 h	7.720	3.385 to 12.05	Yes	****	<0.0001
500:Carboxyl 24 h vs. 500:NF 24 h	-0.8350	-5.170 to 3.500	No	ns	>0.9999
500:Carboxyl 24 h vs. 1000:Amine 4 h	-3.073	-7.407 to 1.262	No	ns	0.6371
500:Carboxyl 24 h vs. 1000:Carboxyl 4 h	1.745	-2.590 to 6.080	No	ns	0.9998
500:Carboxyl 24 h vs. 1000:NF 4 h	4.775	0.4404 to 9.110	Yes	*	0.0134
500:Carboxyl 24 h vs. 1000:Amine 24 h	2.990	-1.345 to 7.325	No	ns	0.6940
500:Carboxyl 24 h vs. 1000:Carboxyl 24 h	4.128	-0.2071 to 8.462	No	ns	0.0871
500:Carboxyl 24 h vs. 1000:NF 24 h	3.143	-1.192 to 7.477	No	ns	0.5875
500:Carboxyl 24 h vs. 5000:Amine 4 h	-6.973	-11.31 to -2.638	Yes	****	<0.0001
500:Carboxyl 24 h vs. 5000:Carboxyl 4 h	-0.6525	-4.987 to 3.682	No	ns	>0.9999
500:Carboxyl 24 h vs. 5000:NF 4 h	3.010	-1.325 to 7.345	No	ns	0.6804
500:Carboxyl 24 h vs. 5000:Amine 24 h	-3.373	-7.707 to 0.9621	No	ns	0.4262
500:Carboxyl 24 h vs. 5000:Carboxyl 24 h	-1.348	-5.682 to 2.987	No	ns	>0.9999
500:Carboxyl 24 h vs. 5000:NF 24 h	1.823	-2.512 to 6.157	No	ns	0.9995
500:NF 24 h vs. 1000:Amine 4 h	-2.238	-6.572 to 2.097	No	ns	0.9842
500:NF 24 h vs. 1000:Carboxyl 4 h	2.580	-1.755 to 6.915	No	ns	0.9113
500:NF 24 h vs. 1000:NF 4 h	5.610	1.275 to 9.945	Yes	***	0.0007
500:NF 24 h vs. 1000:Amine 24 h	3.825	-0.5096 to 8.160	No	ns	0.1800
500:NF 24 h vs. 1000:Carboxyl 24 h	4.963	0.6279 to 9.297	Yes	**	0.0073
500:NF 24 h vs. 1000:NF 24 h	3.978	-0.3571 to 8.312	No	ns	0.1266
500:NF 24 h vs. 5000:Amine 4 h	-6.138	-10.47 to -1.803	Yes	****	<0.0001
500:NF 24 h vs. 5000:Carboxyl 4 h	0.1825	-4.152 to 4.517	No	ns	>0.9999
500:NF 24 h vs. 5000:NF 4 h	3.845	-0.4896 to 8.180	No	ns	0.1722
500:NF 24 h vs. 5000:Amine 24 h	-2.538	-6.872 to 1.797	No	ns	0.9254
500:NF 24 h vs. 5000:Carboxyl 24 h	-0.5125	-4.847 to 3.822	No	ns	>0.9999
500:NF 24 h vs. 5000:NF 24 h	2.658	-1.677 to 6.992	No	ns	0.8811
1000:Amine 4 h vs. 1000:Carboxyl 4 h	4.818	0.4829 to 9.152	Yes	*	0.0117
1000:Amine 4 h vs. 1000:NF 4 h	7.848	3.513 to 12.18	Yes	****	<0.0001
1000:Amine 4 h vs. 1000:Amine 24 h	6.063	1.728 to 10.40	Yes	***	0.0001
1000:Amine 4 h vs. 1000:Carboxyl 24 h	7.200	2.865 to 11.53	Yes	****	<0.0001
1000:Amine 4 h vs. 1000:NF 24 h	6.215	1.880 to 10.55	Yes	****	<0.0001
1000:Amine 4 h vs. 5000:Amine 4 h	-3.900	-8.235 to 0.4346	No	ns	0.1519
1000:Amine 4 h vs. 5000:Carboxyl 4 h	2.420	-1.915 to 6.755	No	ns	0.9564
1000:Amine 4 h vs. 5000:NF 4 h	6.083	1.748 to 10.42	Yes	***	0.0001
1000:Amine 4 h vs. 5000:Amine 24 h	-0.3000	-4.635 to 4.035	No	ns	>0.9999
1000:Amine 4 h vs. 5000:Carboxyl 24 h	1.725	-2.610 to 6.060	No	ns	0.9998
1000:Amine 4 h vs. 5000:NF 24 h	4.895	0.5604 to 9.230	Yes	**	0.0091
1000:Carboxyl 4 h vs. 1000:NF 4 h	3.030	-1.305 to 7.365	No	ns	0.6667
1000:Carboxyl 4 h vs. 1000:Amine 24 h	1.245	-3.090 to 5.580	No	ns	>0.9999

1000:Carboxyl 4 h vs. 1000:Carboxyl 24 h	2.383	-1.952 to 6.717	No	ns	0.9639
1000:Carboxyl 4 h vs. 1000:NF 24 h	1.398	-2.937 to 5.732	No	ns	>0.9999
1000:Carboxyl 4 h vs. 5000:Amine 4 h	-8.718	-13.05 to -4.383	Yes	****	<0.0001
1000:Carboxyl 4 h vs. 5000:Carboxyl 4 h	-2.398	-6.732 to 1.937	No	ns	0.9610
1000:Carboxyl 4 h vs. 5000:NF 4 h	1.265	-3.070 to 5.600	No	ns	>0.9999
1000:Carboxyl 4 h vs. 5000:Amine 24 h	-5.118	-9.452 to -0.7829	Yes	**	0.0043
1000:Carboxyl 4 h vs. 5000:Carboxyl 24 h	-3.093	-7.427 to 1.242	No	ns	0.6230
1000:Carboxyl 4 h vs. 5000:NF 24 h	0.07750	-4.257 to 4.412	No	ns	>0.9999
1000:NF 4 h vs. 1000:Amine 24 h	-1.785	-6.120 to 2.550	No	ns	0.9996
1000:NF 4 h vs. 1000:Carboxyl 24 h	-0.6475	-4.982 to 3.687	No	ns	>0.9999
1000:NF 4 h vs. 1000:NF 24 h	-1.633	-5.967 to 2.702	No	ns	>0.9999
1000:NF 4 h vs. 5000:Amine 4 h	-11.75	-16.08 to -7.413	Yes	****	<0.0001
1000:NF 4 h vs. 5000:Carboxyl 4 h	-5.428	-9.762 to -1.093	Yes	**	0.0015
1000:NF 4 h vs. 5000:NF 4 h	-1.765	-6.100 to 2.570	No	ns	0.9997
1000:NF 4 h vs. 5000:Amine 24 h	-8.148	-12.48 to -3.813	Yes	****	<0.0001
1000:NF 4 h vs. 5000:Carboxyl 24 h	-6.123	-10.46 to -1.788	Yes	***	0.0001
1000:NF 4 h vs. 5000:NF 24 h	-2.953	-7.287 to 1.382	No	ns	0.7189
1000:Amine 24 h vs. 1000:Carboxyl 24 h	1.138	-3.197 to 5.472	No	ns	>0.9999
1000:Amine 24 h vs. 1000:NF 24 h	0.1525	-4.182 to 4.487	No	ns	>0.9999
1000:Amine 24 h vs. 5000:Amine 4 h	-9.963	-14.30 to -5.628	Yes	****	<0.0001
1000:Amine 24 h vs. 5000:Carboxyl 4 h	-3.643	-7.977 to 0.6921	No	ns	0.2638
1000:Amine 24 h vs. 5000:NF 4 h	0.02000	-4.315 to 4.355	No	ns	>0.9999
1000:Amine 24 h vs. 5000:Amine 24 h	-6.363	-10.70 to -2.028	Yes	****	<0.0001
1000:Amine 24 h vs. 5000:Carboxyl 24 h	-4.338	-8.672 to -0.002872	Yes	*	0.0496
1000:Amine 24 h vs. 5000:NF 24 h	-1.168	-5.502 to 3.167	No	ns	>0.9999
1000:Carboxyl 24 h vs. 1000:NF 24 h	-0.9850	-5.320 to 3.350	No	ns	>0.9999
1000:Carboxyl 24 h vs. 5000:Amine 4 h	-11.10	-15.43 to -6.765	Yes	****	<0.0001
1000:Carboxyl 24 h vs. 5000:Carboxyl 4 h	-4.780	-9.115 to -0.4454	Yes	*	0.0132
1000:Carboxyl 24 h vs. 5000:NF 4 h	-1.118	-5.452 to 3.217	No	ns	>0.9999
1000:Carboxyl 24 h vs. 5000:Amine 24 h	-7.500	-11.83 to -3.165	Yes	****	<0.0001
1000:Carboxyl 24 h vs. 5000:Carboxyl 24 h	-5.475	-9.810 to -1.140	Yes	**	0.0012
1000:Carboxyl 24 h vs. 5000:NF 24 h	-2.305	-6.640 to 2.030	No	ns	0.9763
1000:NF 24 h vs. 5000:Amine 4 h	-10.12	-14.45 to -5.780	Yes	****	<0.0001
1000:NF 24 h vs. 5000:Carboxyl 4 h	-3.795	-8.130 to 0.5396	No	ns	0.1923
1000:NF 24 h vs. 5000:NF 4 h	-0.1325	-4.467 to 4.202	No	ns	>0.9999
1000:NF 24 h vs. 5000:Amine 24 h	-6.515	-10.85 to -2.180	Yes	****	<0.0001
1000:NF 24 h vs. 5000:Carboxyl 24 h	-4.490	-8.825 to -0.1554	Yes	*	0.0321
1000:NF 24 h vs. 5000:NF 24 h	-1.320	-5.655 to 3.015	No	ns	>0.9999
5000:Amine 4 h vs. 5000:Carboxyl 4 h	6.320	1.985 to 10.65	Yes	****	<0.0001
5000:Amine 4 h vs. 5000:NF 4 h	9.983	5.648 to 14.32	Yes	****	<0.0001
5000:Amine 4 h vs. 5000:Amine 24 h	3.600	-0.7346 to 7.935	No	ns	0.2865
5000:Amine 4 h vs. 5000:Carboxyl 24 h	5.625	1.290 to 9.960	Yes	***	0.0007
5000:Amine 4 h vs. 5000:NF 24 h	8.795	4.460 to 13.13	Yes	****	<0.0001
5000:Carboxyl 4 h vs. 5000:NF 4 h	3.663	-0.6721 to 7.997	No	ns	0.2536
5000:Carboxyl 4 h vs. 5000:Amine 24 h	-2.720	-7.055 to 1.615	No	ns	0.8527
5000:Carboxyl 4 h vs. 5000:Carboxyl 24 h	-0.6950	-5.030 to 3.640	No	ns	>0.9999
5000:Carboxyl 4 h vs. 5000:NF 24 h	2.475	-1.860 to 6.810	No	ns	0.9434
5000:NF 4 h vs. 5000:Amine 24 h	-6.383	-10.72 to -2.048	Yes	****	<0.0001
5000:NF 4 h vs. 5000:Carboxyl 24 h	-4.358	-8.692 to -0.02287	Yes	*	0.0469
5000:NF 4 h vs. 5000:NF 24 h	-1.188	-5.522 to 3.147	No	ns	>0.9999
5000:Amine 24 h vs. 5000:Carboxyl 24 h	2.025	-2.310 to 6.360	No	ns	0.9965
5000:Amine 24 h vs. 5000:NF 24 h	5.195	0.8604 to 9.530	Yes	**	0.0033
5000:Carboxyl 24 h vs. 5000:NF 24 h	3.170	-1.165 to 7.505	No	ns	0.5679
